# Splicing analyses for variants in MMR genes: best practice recommendations from the European Mismatch Repair Working Group

**DOI:** 10.1038/s41431-022-01106-w

**Published:** 2022-06-09

**Authors:** Monika Morak, Marta Pineda, Alexandra Martins, Pascaline Gaildrat, Hélène Tubeuf, Aurélie Drouet, Carolina Gómez, Estela Dámaso, Kerstin Schaefer, Verena Steinke-Lange, Udo Koehler, Andreas Laner, Julie Hauchard, Karine Chauris, Elke Holinski-Feder, Gabriel Capellá

**Affiliations:** 1 Medizinische Klinik und Poliklinik IV, Campus Innenstadt, Klinikum der Universität München, Munich, Germany; 2 MGZ – Medizinisch Genetisches Zentrum, Munich, Germany; 3 Hereditary Cancer Program, Catalan Institute of Oncology-IDIBELL, ONCOBELL Program, L’Hospitalet, Barcelona, Spain; 4 Centro de Investigación Biomédica en Red de Cáncer (CIBERONC), Madrid, Spain; 5 Inserm U1245, UNIROUEN, Normandie Univ, F-76000 Rouen, France; 6 Interactive Biosoftware, Rouen, France

**Keywords:** Gene expression analysis, Clinical genetics

## Abstract

Over 20% of the DNA mismatch repair (MMR) germline variants in suspected Lynch syndrome patients are classified as variants of uncertain significance (VUS). Well-established functional assays are pivotal for assessing the biological impact of these variants and provide relevant evidence for clinical classification. In our collaborative European Mismatch Repair Working Group (EMMR-WG) we compared three different experimental approaches for evaluating the effect of seven variants on mRNA splicing in MMR genes: (i) RT-PCR of full-length transcripts (FLT), (ii) RT-PCR of targeted transcript sections (TTS), both from patient biological samples and (iii) minigene splicing assays. An overall good concordance was observed between splicing patterns in TTS, FLT and minigene analyses for all variants. The FLT analysis depicted a higher number of different isoforms and mitigated PCR-bias towards shorter isoforms. TTS analyses may miss aberrant isoforms and minigene assays may under/overestimate the severity of certain splicing defects. The interpretation of the experimental findings must be cautious to adequately discriminate abnormal events from physiological complex alternative splicing patterns. A consensus strategy for investigating the impact of MMR variants on splicing was defined. First, RNA should be obtained from patient’s cell cultures (such as fresh lymphocyte cultures) incubated with/without a nonsense-mediated decay inhibitor. Second, FLT RT-PCR analysis is recommended to oversee all generated isoforms. Third, TTS analysis and minigene assays are useful independent approaches for verifying and clarifying FLT results. The use of several methodologies is likely to increase the strength of the experimental evidence which contributes to improve variant interpretation.

## Introduction

Lynch syndrome (LS) is an autosomal dominantly inherited predisposition for early-onset colorectal cancer and other tumors, characterized by specific tumor features and the presence of a heterozygous germline pathogenic variant leading to loss of function of one of the major MMR genes (*MLH1*, *MSH2*, *MSH6* or *PMS2*) [1]. However, in over 20% of LS-suspected patients, a MMR variant of uncertain significance (VUS, class 3 variant) is detected, which does not inform clinical interpretation [2, 3].

An important proportion of MMR variants (up to 36%) affect RNA splicing [4–6]. Intronic or exonic variants can modify the strength of native splice sites, create new splice sites, affect branch point sites, or alter splicing regulatory elements [7, 8]. While variant-induced loss of native splice sites is generally well predicted bioinformatically, their exact consequences on mRNA splicing are difficult to anticipate [9–11]. Furthermore, in silico predictions were described as less reliable for variants mapping within the less conserved positions of consensus splice sites, for those affecting branchpoints or altering enhancer/silencer elements [4, 5]. Recently developed computational tools hold great promise for better pinpointing variants that alter reference splice sites, branchpoints or splicing regulatory elements thus helping to prioritize VUS for experimental splicing analyses [9–12].

Pathogenic assessment of MMR VUS takes into account functional data including those produced by splicing analyses [13], according to InSiGHT [14] and ACMG/AMP guidelines [15]. Also, the later emphasizes the need of well-established functional assays. However, recommended procedures for appropriate RNA splicing analyses in MMR genes are still lacking.

The aim of this collaborative study within the European Mismatch Repair Working Group (EMMR-WG) was to establish an improved practice strategy for splicing analyses of MMR variants. For this purpose, we compared three different approaches for assessing the effect on splicing of seven MMR variants, which allowed us to identify points critical for experimental design and data interpretation.

## Materials and methods

### Patients and samples

Patients provided their informed consent compliant with the respective national ethical standards of Spain or Germany. Biological specimens were obtained for six LS-suspected cases: three from Barcelona (BCN) and three from Munich (MUC) (Table 1). RNA samples and corresponding cDNA products were prepared from fresh lymphocytes followed by short-term culture in presence/absence of puromycin (cDNA+P/cDNA-P) and from blood samples collected into PAXgene tubes (PAX). Methods and reagents for sample processing, RNA isolation and cDNA synthesis are detailed in Supplementary Methods and Supplementary Table 1.

**Table 1:** List of the seven variants investigated in splicing analyses including the patients' phenotype (tumor type, age at diagnosis in years (y), family history of cancers), and tumor specification in terms of immunohistochemical protein staining (IHC) and high microsatellite instability (MSI-H).

Gene and variant	cDNA from	Patient phenotype, age at diagnosis, and family history	Tumor specification
*MSH2* c.211G>C p.(Gly71Arg)	BCN	Colon cancer of hepatic flexure at 45 y; sister with endometrial cancer at 58 y, mother with skin cancer at 68 y.	MSI-H, IHC loss of MSH2/MSH6 in colon cancer in this patient and in three additional tumors from unrelated variant carriers
*MSH2* c.1276G>A p.(Gly426Arg)	BCN	Ovarian cancer at 42 y and cecum colon cancer at 62 y; daughter with endometrial cancer at 30 y.	MSI-H, IHC loss of MSH2/MSH6 in colon cancer in this patient, her daughter, and one additional unrelated patient with this variant
*MSH2* c.2459-12A>G p.?	MUC	Right-sided colon cancer at 52 y; brother with CRC at 35 y, melanoma at 40 y, larynx cancer at 48 y, grandmother with CNS cancer at 55 y.	MSI-H, IHC loss of MSH2/MSH6 in colon cancer in this patient and in two additional unrelated patients with this variant
*MSH6* c.1894A>G p.(Lys632Glu)	BCN	Synchronous ovarian and endometrial cancers at 78 y.	IHC loss of MSH6 in ovarian and endometrial cancer in this patient
*MLH1* c.1039-2A>T p.?	MUC	Colon and prostate cancers at 57 y, colon and duodenal cancers at 66 y; father, brother and sister with CRC.	MSI-H, IHC loss of MLH1/PMS2 in duodenal cancer
*MLH1* c.1217G>A(;)1989+3dup p.(Ser406Asn)(;)p.?	MUC	Right-sided colon cancer at 44 y.	MSI-H, IHC loss of MLH1/PMS2 in colon cancer

*BCN* Barcelona, *MUC* Munich.

### Experimental splicing analyses

Different experimental approaches for splicing analysis were performed (Supplementary Fig. 1). cDNA products were shared between BCN and MUC. In BCN, targeted transcript sections (TTS) were amplified by RT-PCR; whereas in MUC, full-length transcripts (FLT) were amplified by long-range RT-PCR, and TTS analyses were performed as a complementary approach. Rouen (URO) performed cell-based minigene splicing assays by using genomic DNA as cloning source for minigene preparation. Experimental procedures for splicing analyses, results interpretation, RefSeq transcript sequences and nomenclature used for splicing events are detailed in Supplementary Methods and Supplementary Tables 1–3. In brief, levels of aberrant transcripts/isoforms were estimated from RT-PCR products on Sanger sequencing electropherograms in carrier samples and controls. The effect of a variants was assigned as “splice-defect” when aberrant splicing was found with high intensity (>30%) of the total amount of transcripts [16]. When the absence of normally-spliced transcript was proven for the variant allele, the effect was assigned as “complete splicing defect”. On the other hand, variants were designated as “splice-neutral” when no effect on splicing was observed. Results obtained with the different approaches were compared. Differences were regarded as major when the interpretation of the results lead to divergent variant classification based on splicing results.

MMR variants were classified according to the current version of the InSiGHT’s Mismatch Repair Gene Variant Classification documentation (Version 2.4, June 2018, https://www.insight-group.org/content/uploads/2018/08/2018-06_InSiGHT_VIC_v2.4.pdf). The obtained results were submitted to the “Global Variome shared LOVD” and can be accessed at https://databases.lovd.nl/shared/references/DOI:10.1038/s41431-022-01106-w.

### Bioinformatics predictions

In silico predictions of MMR variants using SpliceSiteFinder-like, MaxEntScan and SpliceAI are detailed in Supplementary Methods.

## Results

Experimental splicing analyses of seven MMR variants were conducted in three independent laboratories with different approaches: RT-PCR analyses of patients’ RNA (TTS and FLT) and cell-based minigene splicing assays (Table 2, Supplementary Table 4 and Supplementary Fig. 2).

**Table 2:** Summary of the results obtained in the experimental splicing analyses performed in this study.

*MSH2* c.211G>C p.(Gly71Arg)				
**Experimental approach**	**TTS MSH2 E1-4 (BCN)**	**FLT MSH2 E1-16 (MUC)**	**TTS MSH2 E1-3 (MUC)**	**pCAS2.MSH2.ex1 minigene (URO)**
Alternatively spliced transcript(s) detected at a higher proportion in carriers than controls (% in Sanger sequence)	r.195_211del, −P: 10%; +P: 20%	r.195_211del, −P: 5%; +P: 25% r.-16_211del, −P/+P: 5%	n.a. (r.-16_211del, −P/+P: 100%)	n.a.
Normally spliced transcript produced by variant allele (%)	0%	0%	0%	n.a.
Splicing effect	Complete	Complete	n.a.	n.a.
Consensus splicing effec	Complete splicing defect. Variant activates cryptic splice sites in exon 1: r.195_211del (p.Tyr66Serfs*10) and r.-16_211del (p.?)			
***MSH2*** **c.1276G>A p.(Gly426Arg)**				
**Experimental approach**	**TTS MSH2 E6-13 (BCN)**	**FLT MSH2 E1-16 (MUC)**	**TTS MSH2 E5-8 (MUC)**	**pCAS2.MSH2.ex7 minigene EA-B (URO)**
Alternatively spliced transcript(s) detected at a higher proportion in carriers than controls (% in Sanger sequence)	r.1229_1276del, −P: 30-50%; +P: 50%	r.1229_1276del, −P: ~0-5%; +P: ~25%	r.1229_1276del, −P: 30%; +P: 60%	r.1229_1276del, >99%
Normally spliced transcript produced by variant allele (%)	0%	0%	0%	<1%
Splicing effect	Complete	Complete	Complete	Complete
Consensus splicing effect	Complete splicing defect. Variant activates cryptic splice site in exon 7 resulting in an in-frame deletion affecting a functional protein domain: r.1229_1276del (p.Ile411_Gly426del)			
***MSH2*** **c.2459-12A>G p.?**				
**Experimental approach**	**TTS MSH2 E14-16 (BCN)**	**FLT MSH2 E1-16 (MUC)**	**TTS MSH2 E13-15 (MUC)**	**pCAS2.MSH2.ex15 minigene EA-B (URO)**
Alternatively spliced transcript(s) detected at a higher proportion in carriers than controls (% in Sanger sequence)	r.2458_2459insATTTCTTATAG, −P: 0-5%; +P: ~20%	r.2458_2459insATTTCTTATAG, −P: 0-5%; +P: 15–35%	r.2458_2459insATTTCTTATAG, −P: 0-5%; +P: 15–35%	r.2458_2459insATTTCTTATAG
Normally spliced transcript produced by variant allele (%)	n.a.	n.a.	n.a.	0%
Splicing effect	Yes, unknown strength	Yes, unknown strength	Yes, unknown strength	Complete
Consensus splicing effect	Complete splicing defect, creates a new 3’splice site inserting the last 11 nucleotides of intron 14 disrupting the reading frame: r.2458_2459insATTTCTTATAG (p.Gly820Aspfs*4)			
***MSH6*** **c. 1894A>G p.(Lys632Glu)**				
**Experimental approach**	**TTS MSH6 E3-5 (BCN)**	**FLT MSH6 E1-10 or long TTS E3-10 (MUC)**	**TTS MSH6 E3-5 (MUC)**	**pCAS2-MSH6-ex4 minigene EA-B (URO)**
Alternatively spliced transcript(s) detected at a higher proportion in carriers than controls (% in Sanger sequence)	n.a.	None	n.a.	n.a.
Normally spliced transcript produced by variant allele (%)	100%	100%	100%	n.a.
Splicing effect	n.a.	No	n.a.	n.a.
Consensus splicing effect	Splice-neutral missense variant			
***MLH1*** **c.1039-2A>T p.?**				
**Experimental approach**	**TTS MLH1 E11-15 (BCN)**	**FLT MLH1 E1-19 (MUC)**	**TTS MLH1 E10-14 (MUC)**	**pCAS2.MLH1.ex12 minigene EA-B (URO)**
Alternatively spliced transcript(s) detected at a higher proportion in carriers than controls (% in Sanger sequence)	r.1039_1409del, −P: ~40%; +P: ~65%	r.1039_1409del, −P: 0%; +P: 15% r.678_1409del, −P: 10%; +P: 15% r.885_1409del, −P: 0%; +P: 5% r.1039_1051del, −P: 0%; +P: <5%	r.1039_1409del, −P: ~10%; +P: ~30%	r.1039_1409del (39%) r.1039_1051del (61%)
Normally spliced transcript produced by variant allele (%)	n.a.	n.a.	n.a.	0%
Splicing effect	Yes, unknown strength	Yes, unknown strength	Yes, unknown strength	Complete
Consensus splicing effect	Complete splicing defect. Variant activates cryptic splice sites: r.678_1409del (p.Glu227_Arg470del), r.885_1409del (p.Ser295_Pro469del); r.1039_1409del (p.Thr347Lysfs*8) and r.1039_1051del p.(Thr347Aspfs*16)			
***MLH1*** **c.1217G>A p.(Ser406Asn) co-occurring in-trans with** ***MLH1*c.1989+3dup p.? (c. [1217G>A];[1989+3dup])**				
***MLH1*** **c.1217G>A p.(Ser406Asn)**				
**Experimental approach**	**TTS MLH1 E11-15 (BCN)**	**FLT MLH1 E1-19 (MUC)**	**TTS MLH1 E11-17 (MUC)**	**pCAS2.MLH1.ex12 minigene EA-B (URO)**
Alternatively spliced transcript(s) detected at a higher proportion in carriers than controls (% in Sanger sequence)	None	None	None	None [39]
Normally spliced transcript produced by variant allele (%)	100%	100%	100%	100%
Splicing effect	No	No	No	No
		No, in trans with a pathogenic variant (c.1989+3dup)		
Consensus splicing effect	Splice-neutral missense variant: r.1217G>A (p.Ser406Asn)			
***MLH1*** **c.1989+3dup p.?**				
**Experimental approach**	**TTS MLH1 E15-19 (BCN)**	**FLT MLH1 E1-19 (MUC)**	**TTS MLH1 E11-17 (MUC)**	**pCAS2.MLH1.E17-18 minigene E1A-1B (URO)**
Alternatively spliced transcript(s) detected at a higher proportion in carriers than controls (% in Sanger sequence)	r.1897_1989del, −P/+P: ~75%	r.1897_1989del, −P/+P: 50%	None, but allelic loss of co-occurring exonic variant	r.1897_1989del, 85% r.1989_1990ins[1989+1_1989+31; 1989+3dup], ins IVS17p = 15%
Normally spliced transcript produced by variant allele (%)	n.a.	n.a.	n.a.	0%
Splicing effect	Yes, unknown strength	Yes, unknown strength	Absence of r.1217G	Complete
		Complete		
Consensus splicing effect	Complete splicing defect, activates cryptic splice site in exon 17 resulting in an in-frame deletion affecting a functional protein domain: r.1897_1989del (p.Glu633_Glu663del)			

Underlined isoforms indicate “aberrant” transcripts that were under-represented/undetected in controls. See further details and results obtained with PAXgene samples in Supplementary Table 4. +P: cDNA from lymphocyte culture incubated with puromycin, −P: cDNA from lymphocyte culture not incubated with puromycin; BCN Barcelona, C controls, FLT full-length transcript analysis, ns transcripts normally-spliced transcripts, MUC Munich, n.a. not analyzable, TTS targeted transcript section analysis, URO Rouen.

### *MSH2* exon 1 c.211 G>C p.(Gly71Arg)

*MSH2* c.211G>C, which affects the last nucleotide of *MSH2* exon 1, is predicted to decrease the strength of the 5’ss (Supplementary Table 5). TTS RT-PCR analysis from exon 1 (c.83) to exon 4 identified an aberrant *MSH2* transcript lacking the last 17 nucleotides of exon 1 (Δ1q(17), r.195_211del (p.Tyr66Serfs*10)), with an estimated intensity of 20% and 10% of the total amount of transcripts in cDNA+P/−P samples, respectively, not seen in controls. Sequencing analysis of RT-PCR products corresponding to normally-spliced transcripts (ns) transcripts revealed monoallelic expression of WT allele (no detection of r.211G>C) consistent with a complete splicing defect. In contrast, TTS RT-PCR analysis from exon 1 (c.-41) to exon 3 presented an isoform lacking the last 227 nucleotides of exon 1 (Δ1q(227), r.-16_211del) with an estimated intensity of 20% and 100% in controls and in patient samples, respectively. In the FLT analysis the major aberrant isoform Δ1q(17) was present with 25% and 5% intensity in cDNA+P/–P samples, and an additional isoform Δ1q(227) was observed with 5% intensity in the cDNA+P sample. Sequencing of RT-PCR products revealed monoallelic expression of WT, indicating complete splicing defect. Aberrant transcripts were not present in controls.

*MSH2* c.211G>C could not be tested in the minigene assay as the pCAS2 minigene vector is not suited to test the impact of variants mapping to/near terminal exons [17].

### *MSH2* exon 7 c.1276 G>A p.(Gly426Arg)

*MSH2* c.1276G>A is located at the last position of exon 7 and is predicted to decrease the strength of the 5’ss of this exon (Supplementary Table 5). TTS RT-PCR analysis from *MSH2* exons 6 to 13 and exons 5 to 8 identified an aberrant isoform lacking the last 48 nucleotides of exon 7 (Δ7q(48), r.1229_1276del (p.Ile411_Gly426del)), reaching 50% and 30–50% in cDNA+P/−P samples, and not observed in control samples. FLT amplification also detected Δ7q(48) with intensities of 20–30% and 10–20% only in cDNA+P/-P samples. Sequencing analysis of the RT-PCR products indicated a complete splicing defect (no detection of r.1276G>A). The pCAS2.MSH2.ex7.c.1276G>A minigene construct produced >99% aberrant Δ7q(48) transcripts, concordant with the complete splicing defect observed in both TTS and FLT analyses.

### *MSH2* intron 14 c.2459–12A>G p.?

Bioinformatics analysis of *MSH2* c.2459-12A>G predicted weakening of the natural 3’splice site and creation of a *de novo* 3’ splice site at the end of intron 14 (Supplementary Table 5). TTS RT-PCR analysis of *MSH2* exons 14 to 16, detected an aberrant transcript that retained the last 11 nucleotides of intron 14 (▾15p(11), r.2458_2459insATTTCTTATAG (p.Gly820Aspfs*4)) with an intensity of ~20% in cDNA+P, and 0–5% in cDNA–P. The presence of ▾15p(11) was also detected in two PAXgene samples (PAX1 and PAX2) obtained from the same patient. TTS RT-PCR of *MSH2* exons 13 to 15 also detected ▾15p(11) with similar intensities of 15–35% and 0–5% in cDNA+P and cDNA-P, respectively. The aberrant product was not observed in control samples. Previously reported FLT RT-PCR analysis revealed ▾15p(11) with 15–35% in cDNA+P, and 0–5% in cDNA–P and 0% in PAX2, reaching the 30% splicing defect threshold in cDNA+P [16]. FLT was not amplifiable in PAX1. Results obtained from cDNA+P/−P strongly suggested that the out-of-frame ▾15p(11) transcripts were targeted for degradation by the NMD system. Minigene data obtained with pCAS2.MSH2.ex15.c.2459–12A>G revealed that c.2459-12A>G causes total loss of normally spliced transcripts as it exclusively produces ▾15p(11).

### *MSH6* exon 4 c.1894A>G p.(Lys632Glu)

*MSH6* c.1894A>G is located in the center of exon 4 and was not predicted to impair splicing (Supplementary Table 5). In patient samples, the analysis of *MSH6* exons 3–5 by TTS RT-PCR revealed partial skipping of exon 4 (Δ4, r.628_3172, p.(Val210Metfs*21)), with intensities of 40–50% and 10–20% in cDNA+P/−P, similar to controls). Therefore, high levels of alternative splicing observed in controls precluded interpretation. The amplification of the 4.2 kb *MSH6* FLT failed in the cDNA+P/−P samples. Thus, we performed a long TTS RT-PCR analysis focused on *MSH6* exons 3–10 (3.6 kb) detecting Δ4 with 20–25% and 10% intensities in cDNA+P/−P samples, equivalent to control samples. Sequencing analysis of the RT-PCR products revealed biallelic expression suggesting the absence of a significant splicing defect associated to c.1894A>G. The large exon 4 was not properly spliced in the pCAS2.MSH6.ex4.WT minigene construct, which precluded the analysis of the c.1894A>G variant in the minigene splicing assay (Supplementary Table 4).

### *MLH1* intron 11 c.1039-2A>T p.?

Bioinformatics analysis of *MLH1* c.1039-2A>T predicted the destruction of the 3’splice site of exon 12 and the potential creation of a de novo 3’splice site within the exon (Supplementary Table 5). TTS RT-PCR analysis of *MLH1* exons 11–15 detected an isoform lacking exon 12 (Δ12, r.1039_1409del, p.(Thr347Lysfs*8)) with an intensity of 60–70% in cDNA+P, and 30–50% in cDNA–P. In the PAXgene sample (PAX) from the same patient the aberrant transcript represented 90% of all transcripts compared to the 5% detected in controls. This over-representation was regarded as not interpretable. Subsequently, TTS analysis of *MLH1* exons 10–14 was set up detecting 30% and 10% Δ12 in cDNA+P/−P products, and 0–10% in controls. Previously reported FLT analysis revealed four aberrant transcripts affecting exon 12 in cDNA+P with a 35% total intensity including 15% Δ12, 15% Δ9–12 (r.678_1409del, p.Glu227_Arg470del), 5% Δ11–12 (r.885_1409del, p.Ser295_Pro469del) and <5% Δ12p(13) (r.1039_1051del, p.Thr347Aspfs*16) [16]. In cDNA-P 10% Δ9–12 was found, and in PAX 5% Δ9–12, and 5% Δ12. In controls, Δ12 was present with 5–10% in cDNA+P, whereas Δ9–12, Δ11–12 and Δ12p(13) were absent. Though the threshold for splicing defect (>30%) was reached, we could not rule out the production of ns transcripts from the variant allele given the absence of an exonic tracer (co-occurring exonic variant) in this sample. The pCAS2.MLH1.ex12.c.1039-2A>T minigene assay indicated that c.1039-2A>T causes total loss of ns transcripts due to the production of two aberrant isoforms lacking either the first 13 nucleotides of exon 12 (61% Δ12p(13), r.1039_1051del p.(Thr347Aspfs*16)), or the entire exon 12 (39% Δ12), both consistent with the in silico predictions for c.1039-2A>T. The corresponding WT minigene produced only very low amounts of the aberrant isoforms (1.8% Δ12, and 4% Δ12q(260) r.1150_1409del p.(Val384Lysfs*8)).

### *MLH1* exon 12 c.1217 G>A p.(Ser406Asn) co-occurring with c.1989+3dup p.?

Bioinformatics analyses did not predict a defect for c.1217G>A located within exon 12 but anticipated a splicing anomaly for c.1989+3dup due to destruction of the 5’ splice site of exon 17 (Supplementary Table 5). TTS RT-PCR analysis of *MLH1* exons 11 to 15 (to interrogate c.1217G>A) did not detect aberrant transcripts in cDNA+P/-P, whereas a PAX sample from the same patient was not amplifiable. Expression was biallelic as determined by RT-PCR product sequencing. The FLT analysis [16] and pCAS2.MLH1.ex12 minigene assay also revealed the same splicing pattern for c.1217G>A and controls. TTS analysis of *MLH1* exons 15 to 19 (to interrogate c.1989+3dup) detected high levels of aberrant transcripts lacking exon 17 (Δ17, r.1897_1989del, p.Glu633_Glu663del) in all available samples (cDNA+P/−P and PAX) ranging from 50 to 80% intensity, whereas controls displayed 5–10% Δ17. Previously reported FLT analysis also revealed Δ17 with 50% intensity in cDNA+P/−P, but PAX was not amplifiable in identical FLT RT-PCR conditions [16]. This in-frame splicing defect (no NMD expected) met the threshold (>30%) for the FLT. The targeted analysis of Δ17 transcripts revealed monoallelic expression of r.1217G>A, proving 100% splicing defect for c.1989+3dup consistent with an in-trans phasing of these co-occurring variants (c. [1217G>A];[1989+3dup]). Results from the pCAS2.MLH1.ex17-18 c.1989+3dup minigene assay showed that c.1989+3dup causes total loss of normally-spliced transcripts due to the production of two aberrant transcripts, one lacking exon 17 (85% Δ17) and the other retaining of the first 32 nucleotides of intron 17 (15% ▾17q(32) r.1989_1990ins[1989+1_1989+31; 1989+3dup] p.(Asn665Glnfs*).

### Comparison between in silico predictions and experimental splicing results

Of the seven selected variants, experimental splicing analyses showed splicing defect in the five variants bioinformatically predicted to cause splicing defects (Supplementary Table 5 and Supplementary Fig. 3). Two variants not predicted to affect splicing were experimentally ascertained as “splice-neutral”.

### Differences between results obtained in TTS, FLT and minigene analyses

Regarding TTS and FLT cDNA analyses (Supplementary Tables 3 and  4), samples from short-term lymphocyte cultures yielded the best results as compared with PAXgene specimens, probably due to poor cDNA quality in the later as previously reported [16]. For lymphocyte cultures, incubation with an NMD inhibitor is often needed to detect transcripts undergoing NMD. The cDNA synthesis is also a crucial step, as enzymes and primers influence cDNA amplification performance. In the TTS analysis primer choice potentially selects against certain isoforms, so that in the FLT a higher number of different isoforms is detectable (notably in the study of *MSH2* c.211G>C and *MLH1* c.1039-2A>T). On the other hand, the levels of alternative and aberrant transcripts may be abnormally elevated in TTS analyses, attributed to a PCR-bias preferentially amplifying shorter isoforms [18]. The PCR-bias is lower for the longer FLT products better allowing the definition of thresholds for splicing defect and for alternative splicing [16]. Another advantage of FLT analysis is the possibility of assessing the relative expression level of a co-occurring informative variant (as in the analysis of *MLH1* c.1217G>A and c.1989+3dup) via specific nested TTS PCR amplifications.

Minigene splicing assays allow analysis of VUS regardless of patient’s RNA availability. They also facilitate interpretation of the results given the monoallelic character of the approach. Overall, the minigene results obtained in this study agreed with those obtained with patient’s RNA thereby confirming variants’ effects on RNA splicing. Still, minigene testing was not possible for the VUS located in *MSH2* exon 1 and *MSH6* exon 4 for technical reasons (Supplementary Table 4). In addition, some isoforms were detected in the minigene assays but not in patient’s RNA (as seen for *MLH1* c.1039-2A>T and c.1989+3dup), likely reflecting either the minigene’s NMD-resistance and high sensitivity, or a minigene-specific artificial splicing pattern. Also, the minigenes used were unable to capture complex splicing defects implicating multiple exons (e.g. Δ9–12 and Δ11-12 associated to *MLH1* c.1039-2A>T in FLT analyses of patient’s RNA).

### Classification of MMR variants

The ultimate usefulness of a functional assay is to refine variant classification. The current version of the InSiGHT’s MMR Gene Variant Classification documentation (version 2.4) highlights the need of confirming splicing aberration in a minigene assay or an additional RNA assay from an independent laboratory if it is not a predicted splice site mutation. Following these rules and considering all available evidence, five variants (*MSH2* c.211G>C, c.1276G>C and c.2459-12A>G, *MLH1* c.1039-2A>T and c.1989+3dup) affected RNA splicing and were classified as pathogenic (class 5) (Table 3). Of note, the complete splicing defects confirmed in the minigene assay for *MLH1* c.1039-2A>T and *MSH2* c.2459-12A>G contributed to their robust interpretation as class 5. Finally, the splice-neutral *MSH6* c.1894A>G (p.Lys632Glu) remained a class 3 missense variant (unknown effect at protein level) while the *MLH1* c.1217G>A (p.Ser406Asn) was splice-neutral and classified as class 1 given its co-occurrence in trans with a splicing defect variant in a patient analyzed in this study, and its proficiency in previously reported in vitro MMR assays (Table 3).

**Table 3:** Relevant evidence for the pathogenicity assessment of the MMR variants analyzed in this study.

Gene	variant	Predicted proteinchange	Clinical characteristics of carriers	Population frequency (GnomAD all non-cancer)	In silico predictions^a^		Functional analyses		Posterior probability of pathogenicity	Other relevant information	ClinVar interpretation (*n*) (Review status)^c^	Final classification^d^
					Splicing	Protein function	cDNA splicing analysis^b^	Protein analysis				
*MSH2*	c.211G>C	p.(Gly71Arg)	4 carriers, MSH2/MSH6- tumors [37] (Table 1)	NR	Splicing defect	Weak/null impact	*Complete splicing defect:* r.195_211del, p.Tyr66Serfs*10, r.-16_211del, p.?	Normal function (p.Gly71Arg) LOF score: −3.71 [41]	0.65 [37]	–	Class 4 (5)/Class 5 (1) (Class 4–5, **)	Class5
*MSH2*	c.1276G>A	p.(Gly426Arg)	2 carriers, MSH2/MSH6- tumors [37] (Table 1)	NR	Splicing defect	Moderate impact	*Complete splicing defect:* r.1230_1277del, p.Ile411_Gly426del	Normal function (p.Gly426Arg) LOF score: −4.24 [41]	0.66 [37]	In-frame deletion of lever domain. c.1276+1G>A, leading to the same splicing defect as c.1276G>A, was classified as pathogenic by multifactorial analysis.	Class 3 (1)/Class 4 (1) (Conflicting, *)	Class5
*MSH2*	c.2459-12A>G	p.?	3 carriers, MSH2/MSH6- tumors [16] (InSiGHT classifications (Table 1)	NR	Splicing defect	_	*Complete splicing defect:* r.2458_2459insATTTCTTATAG, p.Gly820Aspfs*4	–	–	–	Class 3 (3)/Class 4 (2) (Conflicting, *)	Class5
*MSH6*	c.1894A>G	p.(Lys632Glu)	1 carrier, MSH6-tumors (Table 1)	1.9e-05	Splice-neutral	Weak/null impact	*Splice-neutral missense variant:* r.1894A>G p.Lys632Glu	_	_	_	Class 3 (8) (Class 3, **)	Class3
*MLH1*	c.1039-2A>T	p.?	2 carriers, MLH1/PMS2- tumors [16] (InSiGHT classifications and Table 1)	NR	Splicing defect	_	*Complete splicing defect:* r.678_1409del, p.Glu227_Arg470del; r.885_1409del, p.Ser295_Pro469del; r.1039_1409del, p.Thr347Lysfs*8	_	_	_	Class 4 (1) (Class 4, ***)	Class5
*MLH1*	c.1217G>A	p.(Ser406Asn)	1 carrier, MLH1/PMS2- tumor [16] (Table 1)	8.7e-04 (1.3e-3 NFE, Latino)	Splice-neutral	Weak/null impact	*Splice-neutral missense variant:* r.1217G>A, p.Ser406Asn	MMR proficient (InSiGHT classifications)	<0.001 (InSiGHT classifications)	Variant in trans to a pathogenic c.1989+3dup variant, no indication for CMMRD in the patient, with colon cancer at 44 y	Class 1 (11) / Class 2 (7) / Class 3 (5) (Benign, ***)	Class1
	c.1039-2A>T	p.?		NR	Splicing defect	_	*Complete splicing defect:* r.1897_1989del, p.Glu633_Glu663del	_	_	Variant c.1989+1G>T leading to the same splicing defect as c.1989+3dup was classified as pathogenic by multifactorial analysis	Class 4 (Class 4, ***)	Class5

*NR* not reported.

^a^Splicing predictions according to Supplementary Table 5; protein function prediction according to HCI Cancer Susceptibility Genes Prior Probabilities of Pathogenicity.

^b^See Supplementary Table 4 for further details [40].

^c^Variant classification reported in ClinVar database (https://www.ncbi.nlm.nih.gov/clinvar/, accessed on 1st March 2022).

^d^MMR variant classification according to InSiGHT guidelines v.2.4 (June 2018). InSiGHT classifications: http://www.insight-database.org/classifications.

## Discussion

Experimental splicing analyses are needed not only to determine the exact nature of splicing defects generated by splice site variants but also essential to the biological interpretation of VUS located outside the consensus splice sites. Given the high number of VUS, newly developed in silico tools can be used for helping in functional testing stratification [9–12, 19]. Here, we compared different experimental approaches for evaluating the effect of seven MMR gene variants on mRNA splicing. Based on the obtained results we propose an experimental strategy for VUS splicing analysis in MMR genes.

The interpretation of the splicing defect observed by TTS, FLT and minigene splicing analyses did not significantly differ for all seven variants. Regarding the comparison of TTS and FLT approaches some differences emerged. The amplifiability of cDNA was better in TTS and the relative levels of alternative and aberrant transcripts seemed to be also higher probably due to PCR bias. On the other hand, and as expected, FLT analyses were able to detect more isoforms (Supplementary Table 6). In this context, the set-up of ad hoc TTS analyses for interrogating the allelic balance of informative co-occurring exonic variants proved useful.

However, both TTS and FLT share some limitations (Supplementary Table 6). The use of RNA extracted from peripheral blood (fresh lymphocytes or PAXgene samples) may not reflect disease-relevant tissue-specific splicing alterations potentially produced in other tissues. Of note, LS predisposes to cancer in a variety of organs, mainly colon and endometrium that are mostly not available for analysis. Also, TTS and FLT splicing analyses yield approximate semi-quantitative values from visualization of RT-PCR products in agarose gels and Sanger sequences. In spite of being regarded as sufficient in this context, other technologies such allele-specific expression by SNaPshot [11, 20] or other semiquantitative approaches using high resolution capillary electrophoresis of fragments amplified [21–23] might be used to refine the relative abundance of the transcripts observed.

In agreement with previous work, an overall good concordance was observed between splicing patterns of patient samples and those seen in pCAS-derived minigenes [20, 24, 25]. It is important to note that minigene approaches represent surrogate analytical systems allowing to circumvent the lack of samples from disease-relevant tissues in routine diagnostic practice. However, results derived from artificial constructs should be individually validated with patient RNA data when available. Besides being independent from patient material availability, minigene assays offer straightforward analysis and quantification given its monoallelic nature helping in the establishment of a direct cause–effect relationship between a variant of interest and the observed splicing defect [11, 17, 20, 25] However, its implementation is more laborious and time-consuming, and may not be applicable to all exons and/or genes (Supplementary Table 6). Also, it must be taken into account that the structure of the minigene constructs as well as the cell line selected for transfection may have an impact in the observed splicing patterns [5, 20, 25], The inability of small minigenes, such as the ones used in our study, to identify splicing defect involving multiple exons can be potentially overcome for instance by preparing midigenes (large-size minigenes carrying multiple exons) [26].

All three splicing analyses require hands-on-time for analysis and interpretation. In the future, automated high-throughput strategies suitable for massive parallel sequencing such as targeted short-read RNA-Seq [27] or long-read RNA-Seq [28] will likely replace classical RT-PCR approaches for analyzing RNA harboring a multitude of VUS within different genes. The lessons learnt with the current low-throughput approaches will certainly help in the interpretation of the results obtained.

The majority of variant-induced splicing defects detected thus far for MMR genes were identified by performing TTS analyses and minigene assays [4, 5, 11–13, 20, 25, 29–31]. Physiological alternative splicing of MMR transcripts was reported in patients and controls with variable intensity levels and affecting both single exons and multiple consecutive exons [32–34]. It is thus critical to discriminate variant-induced splicing defects from physiological complex alternative splicing patterns [16, 35, 36]. Only for few MMR variants, different experimental approaches were carried out in parallel [4, 11, 20, 37]. A concerted comparison of splicing methods and results obtained in independent laboratories has, to our knowledge, so far only been performed for *BRCA1* and *BRCA2* variants [23]. The comparison revealed that divergent results for some variants were mainly due to primer choice. Interestingly, the ENIGMA consortium observed a benefit in analytical sensitivity and data consistency when different laboratories used a standardized, universal protocol.

**Fig. 1:**
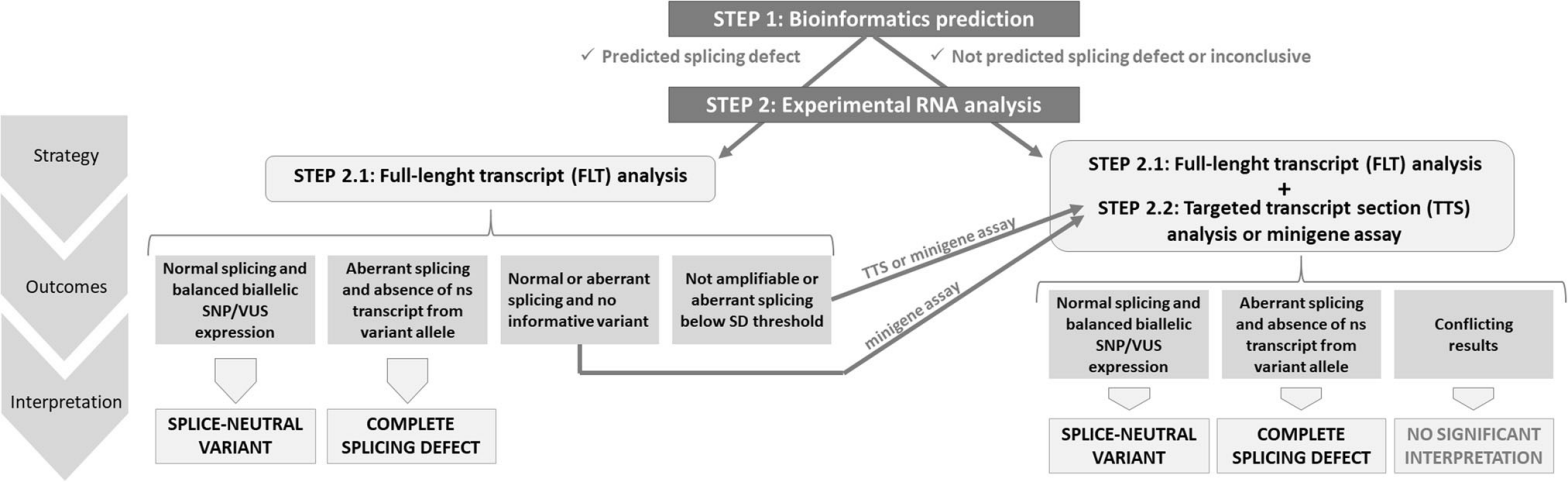
Flowchart of consecutive splicing analyses recommended by the EMMR-WG to investigate MMR variants. Bioinformatic predictions are included as a first step because not predicted splicing aberrations must be confirmed in an additional RNA assay, according to InSiGHT MMR variant classification guidelines (v2.4). The full-length transcript (FLT) analysis is suggested as the second step, with the exception that no patient material is available, or the cDNA is not amplifiable as FLT. In these cases, a target transcript section (TTS) analysis or a minigene assay should be performed in the next step. According to InSiGHT MMR variant classification rules, normal splicing and balanced biallelic expression of a variant in the FLT analysis assign variants as splice-neutral. In absence of an informative variant, the effect of intronic variants on RNA splicing has to be further investigated in a minigene assay. This also applies for inconclusive results from FLT analyses. In contrast, if the FLT analysis demonstrates aberrant splicing and excludes the generation of full-length transcript derived from the variant allele, then the variant can be assigned as complete splice defect, especially if bioinformatics also predicted a splicing defect. Otherwise, its presence has to be verified with an independent method, which can be a TTS analysis in another laboratory, or a minigene assay. Additional analyses are also needed when FLT analyses yield inconclusive results.

Here we define an *EMMR-WG* consensus strategy for investigating the impact of MMR variants on splicing. Blood-derived RNA samples, ideally from patient-derived cell lines (such as the short-term lymphocyte cultures used in this study) are preferred over PAXgene samples as they allow to inhibit NMD. As a first step, we recommend performing FLT analysis for exonic variants or for intronic changes co-occurring with an informative exonic variant of the four MMR genes (Fig. 1) [16]. A normal splicing pattern of a VUS-bearing intron/exon and an apparently balanced biallelic expression of an informative exonic variant allows to assign a VUS as “splice-neutral”. In case that cDNA is not amplifiable in the FLT protocol, or the latter produces inconclusive results, TTS analysis should be performed. If RNA is not available or RNA-data are inconclusive, minigene assays are advised [38].

The good correlation observed between the experimental splicing analysis and the bioinformatics predictions in our limited set of samples supports the combination of this evidence to upgrade pathogenicity classification of this type of variants. When utilizing the current InSiGHT-VIC MMR variant classification criteria, the use of several methodologies is likely to increase the strength of the experimental evidence leading to more clinically actionable variants. The EMMR WG consortium is currently testing the feasibility and potential validity of this approach in an extended series of MMR variants contributed by other centers.

## Conclusion

We recommend FLT analysis as the first step to investigate the effect of MMR VUS likely to impact splicing to get a panoramic view of all generated isoforms. Then, given the complexity of MMR splicing patterns we advise to use TTS and minigene splicing assays as independent strategies for the verification of results or for the clarification of inconclusive observations. The evidence provided here will help in the definition of well-established gene-specific assays to be used in routine practice and the relative weight of the evidence to be considered in the ongoing transition from InSiGHT-VIC to InSiGHT ACMG MMR gene variant classification rules.

## Supplementary information

Supplemental Methods

Supplemental figures' legends

Supplemental Figure 1_Schematic representation of the experimental approaches for evaluating the effect of MMR VUS on mRNA splicing used in this study

Supplemental Figure 2_Sanger sequencing electropherograms from carriers and controls obtained from the experimental approaches used in this work.

Supplemental Figure 3_Schematic representation of the SpliceSiteFinder-like and MaxEntScan algorithms splicing predictions

Supplemental Table 1_Comparative overview of the experimental approaches used in this study

Supplemental Table 2_Specific primer sequences used in this study.

Supplemental Table 3_Cloning and RT-PCR primers for minigene splicing assays

Supplemental Table 4_Overview and comparative analysis of the RT-PCR splicing results

Supplemental Table 5_Summary of in silico splicing predictions

Supplemental Table 6_List of advantages and disadvantages of the different experimental and bioinformatical splicing analyses

## Data Availability

The datasets generated and/or analyzed during the current study are available from the corresponding author on reasonable request.

## References

[1] Lynch HT, de la Chapelle A (1999). Genetic susceptibility to non-polyposis colorectal cancer. J Med Genet.

[2] Müller-Koch Y, Kopp R, Lohse P, Baretton G, Stoetzer A, Aust D (2001). Sixteen rare sequence variants of the hMLH1 and hMSH2 genes found in a cohort of 254 suspected HNPCC (hereditary non-polyposis colorectal cancer) patients: mutations or polymorphisms?. Eur J Med Res.

[3] Peltomäki P, Vasen H (2004). Mutations associated with HNPCC predisposition—update of ICG-HNPCC/INSiGHT mutation database. Dis Markers.

[4] Auclair J, Busine MP, Navarro C, Ruano E, Montmain G, Desseigne F (2006). Systematic mRNA analysis for the effect of MLH1 and MSH2 missense and silent mutations on aberrant splicing. Hum Mutat.

[5] Lastella P, Surdo NC, Resta N, Guanti G, Stella A (2006). In silico and in vivo splicing analysis of MLH1 and MSH2 missense mutations shows exon- and tissue-specific effects. BMC Genom.

[6] Thompson BA, Walters R, Parsons MT, Dumenil T, Drost M, Tiersma Y (2020). Contribution of mRNA splicing to mismatch repair gene sequence variant interpretation. Front Genet.

[7] Cartegni L, Wang J, Zhu Z, Zhang MQ, Krainer AR (2003). ESEfinder: a web resource to identify exonic splicing enhancers. Nucleic Acids Res.

[8] Soret J, Gabut M, Tazi J (2006). SR proteins as potential targets for therapy. Prog Mol Subcell Biol.

[9] Leman R, Gaildrat P, Le Gac G, Ka C, Fichou Y, Audrezet M-P (2018). Novel diagnostic tool for prediction of variant spliceogenicity derived from a set of 395 combined in silico/in vitro studies: an international collaborative effort. Nucleic Acids Res.

[10] Moles-Fernández A, Duran-Lozano L, Montalban G, Bonache S, López-Perolio I, Menéndez M (2018). Computational tools for splicing defect prediction in breast/ovarian cancer genes: how efficient are they at predicting RNA alterations?. Front Genet.

[11] Soukarieh O, Gaildrat P, Hamieh M, Drouet A, Baert-Desurmont S, Frébourg T (2016). Exonic splicing mutations are more prevalent than currently estimated and can be predicted by using in silico tools. PLoS Genet.

[12] Tubeuf H, Charbonnier C, Soukarieh O, Blavier A, Lefebvre A, Dauchel H (2020). Large-scale comparative evaluation of user-friendly tools for predicting variant-induced alterations of splicing regulatory elements. Hum Mutat..

[13] Rhine CL, Cygan KJ, Soemedi R, Maguire S, Murray MF, Monaghan SF (2018). Hereditary cancer genes are highly susceptible to splicing mutations. PLoS Genet.

[14] Thompson BA, Spurdle AB, Plazzer J-P, Greenblatt MS, Akagi K, Al-Mulla F (2014). Application of a 5-tiered scheme for standardized classification of 2,360 unique mismatch repair gene variants in the InSiGHT locus-specific database. Nat Genet.

[15] Richards S, Aziz N, Bale S, Bick D, Das S, Gastier-Foster J (2015). Standards and guidelines for the interpretation of sequence variants: a joint consensus recommendation of the American College of Medical Genetics and Genomics and the Association for Molecular Pathology. Genet Med.

[16] Morak M, Schaefer K, Steinke-Lange V, Koehler U, Keinath S, Massdorf T (2019). Full-length transcript amplification and sequencing as universal method to test mRNA integrity and biallelic expression in mismatch repair genes. Eur J Hum Genet.

[17] Gaildrat P, Killian A, Martins A, Tournier I, Frébourg T, Tosi M (2010). Use of splicing reporter minigene assay to evaluate the effect on splicing of unclassified genetic variants. Methods Mol Biol.

[18] Shagin DA, Lukyanov KA, Vagner LL, Matz MV (1999). Regulation of average length of complex PCR product. Nucleic Acids Res.

[19] Houdayer C, Caux-Moncoutier V, Krieger S, Barrois M, Bonnet F, Bourdon V (2012). Guidelines for splicing analysis in molecular diagnosis derived from a set of 327 combined in silico/in vitro studies on BRCA1 and BRCA2 variants. Hum Mutat.

[20] Tournier I, Vezain M, Martins A, Charbonnier F, Baert-Desurmont S, Olschwang S (2008). A large fraction of unclassified variants of the mismatch repair genes MLH1 and MSH2 is associated with splicing defects. Hum Mutat.

[21] de Garibay GR, Acedo A, Garcia-Casado Z, Gutierrez-Enriquez S, Tosar A, Romero A (2014). Capillary electrophoresis analysis of conventional splicing assays: IARC analytical and clinical classification of 31 BRCA2 genetic variants. Hum Mutat.

[22] Montalban G, Bonache S, Moles-Fernandez A, Gadea N, Tenes A, Torres-Esquius S (2019). Incorporation of semi-quantitative analysis of splicing alterations for the clinical interpretation of variants in BRCA1 and BRCA2 genes. Hum Mutat.

[23] Whiley PJ, de la Hoya M, Thomassen M, Becker A, Brandão R, Pedersen IS (2014). Comparison of mRNA splicing assay protocols across multiple laboratories: recommendations for best practice in standardized clinical testing. Clin Chem.

[24] Gaildrat P, Krieger S, Di Giacomo D, Abdat J, Révillion F, Caputo S (2012). Multiple sequence variants of BRCA2 exon 7 alter splicing regulation. J Med Genet.

[25] van der Klift HM, Jansen AML, van der Steenstraten N, Bik EC, Tops CMJ, Devilee P (2015). Splicing analysis for exonic and intronic mismatch repair gene variants associated with Lynch syndrome confirms high concordance between minigene assays and patient RNA analyses. Mol Genet Genom Med.

[26] Khan M, Cornelis SS, Pozo-Valero MD, Whelan L, Runhart EH, Mishra K (2020). Resolving the dark matter of ABCA4 for 1054 Stargardt disease probands through integrated genomics and transcriptomics. Genet Med.

[27] Yamamoto G, Miyabe I, Tanaka K, Kakuta M, Watanabe M, Kawakami S (2021). SVA retrotransposon insertion in exon of MMR genes results in aberrant RNA splicing and causes Lynch syndrome. Eur J Hum Genet.

[28] Hu Y, Shu X-S, Yu J, Sun M-A, Chen Z, Liu X (2020). Improving the diversity of captured full-length isoforms using a normalized single-molecule RNA-sequencing method. Commun Biol.

[29] Baehring J, Sutter C, Kadmon M, Doeberitz MVK, Gebert J (2006). A ‘nonsense’ mutation leads to aberrant splicing of hMLH1 in a German hereditary non-polyposis colorectal cancer family. Fam Cancer.

[30] Borràs E, Pineda M, Brieger A, Hinrichsen I, Gómez C, Navarro M (2012). Comprehensive functional assessment of MLH1 variants of unknown significance. Hum Mutat.

[31] Pagenstecher C, Wehner M, Friedl W, Rahner N, Aretz S, Friedrichs N (2006). Aberrant splicing in MLH1 and MSH2 due to exonic and intronic variants. Hum Genet.

[32] Charbonnier F, Martin C, Scotte M, Sibert L, Moreau V, Frebourg T (1995). Alternative splicing of MLH1 messenger RNA in human normal cells. Cancer Res.

[33] Clarke LA, Jordan P, Boavida MG (2000). Cell type specificity in alternative splicing of the human mismatch repair gene hMSH2. Eur J Hum Genet.

[34] Genuardi M, Viel A, Bonora D, Capozzi E, Bellacosa A, Leonardi F (1998). Characterization of MLH1 and MSH2 alternative splicing and its relevance to molecular testing of colorectal cancer susceptibility. Hum Genet.

[35] Spurdle AB, Couch FJ, Hogervorst FBL, Radice P, Sinilnikova OM, Group IUGVW. (2008). Prediction and assessment of splicing alterations: implications for clinical testing. Hum Mutat.

[36] Vreeswijk MPG, van der Klift HM (2012). Analysis and interpretation of RNA splicing alterations in genes involved in genetic disorders. Methods Mol Biol.

[37] Vargas-Parra GM, González-Acosta M, Thompson BA, Gómez C, Fernández A, Dámaso E (2017). Elucidating the molecular basis of MSH2-deficient tumors by combined germline and somatic analysis. Int J Cancer.

[38] de la Hoya M, Soukarieh O, López-Perolio I, Vega A, Walker LC, van Ierland Y (2016). Combined genetic and splicing analysis of BRCA1 c.[594-2A>C; 641A>G] highlights the relevance of naturally occurring in-frame transcripts for developing disease gene variant classification algorithm. Hum Mol Genet.

[39] Tricarico R, Kasela M, Mareni C, Thompson BA, Drouet A, Staderini L (2017). Assessment of the InSiGHT interpretation criteria for the clinical classification of 24 MLH1 and MSH2 gene variants. Hum Mutat.

[40] Thompson BA, Martins A, Spurdle AB (2015). A review of mismatch repair gene transcripts: issues for interpretation of mRNA splicing assays. Clin Genet.

[41] Jia X, Burugula BB, Chen V, Lemons RM, Jayakody S, Maksutova M (2021). Massively parallel functional testing of MSH2 missense variants conferring Lynch syndrome risk. Am J Hum Genet.

