# Supplementary material for: Splicing analyses for variants in MMR genes: best practice recommendations from the European Mismatch Repair Working Group
Source: Eur J Hum Genet. 2022 Jun 9;30(9):1051–9. doi: 10.1038/s41431-022-01106-w (PMC9437034; doi:10.1038/s41431-022-01106-w)
Supplement: Supplementary file 1 — Supplemental Methods [file 41431_2022_1106_MOESM1_ESM.docx]

**SUPPLEMENTAL METHODS**

**Selection of MMR variants included in the study**

The selection of MMR variants for *in vitro* studies was based on the followed criteria: identified in LS-suspected individuals, VUS classification at the beginning of this collaborative work and availability of requested biological material.

**Nomenclature**

This study considered the following RefSeq transcript sequences: NM_000251.2 NG_007110.2 for *MSH2*, NM_000179.2 and NG_007111.1 for *MSH6,* and NM_000249.3, NG_007109.2 for *MLH1.* Variant annotation followed HGVS nomenclature by considering the GRCh37 hg19 genome version (http://varnomen.hgvs.org/recommendations). The nomenclature used for splicing events/defects was as follows: (i) the symbol Δ followed by the exon numbers(s) denotes complete deletion (skipping) of the corresponding exon(s), a partial exonic deletion being further indicated either by the letter p for a deletion at the exon’s 5`end, or q for the 3`end, and (ii) ▼ indicates an intronic insertion/retention, this symbol being followed by the number of the adjacent exon [1].

## cDNA analysis protocol by RT-PCR of TTS (targeted transcript sections) in BCN

cDNA generation

Lymphocytes were isolated by Ficoll gradient centrifugation of peripheral blood samples from carriers and controls. Immediately, cells were cultured in PB-MAX™ Karyotyping Medium (Gibco) at 37°C and 5% CO2. After 3-5 days of incubation, the cultures were divided in two, and puromycin was added to a final concentration of 0.25 mg/ml in one of them for 4 to 6 hours before RNA extraction. Total RNA was extracted using Trizol reagent (Invitrogen) according to the manufacturer’s instructions. RNA quality was assessed by electrophoresis using 0.8% agarose gels and RNA integrity number (RIN) was determined (Agilent Bioanalyzer). One microgram or 250 ng of RNA was retrotranscribed using I-script cDNA synthesis kit and random primers or SuperScript II and a mix of random and Oligo-(dT)_18_ primers, according to the manufacturer’s instructions (**Supplemental Table 1**).

cDNA analysis

cDNA amplification of the exon containing the variant of interest and at least one contiguous exon was performed using specific primers and Megamix double polymerase (Microzone) or LA Taq DNA polymerase (Takara Clontech) provided in **Supplemental Table 2**. Aliquots of PCR products were separated by electrophoresis in a 1% agarose gel. The amplified fragments were purified with Exonuclease I plus Shrimp Alkaline Phosphatase (ExoSAP). Sanger sequencing was performed using BigDye Terminator v.3.1 Sequencing Kit (Applied Biosystems, Carlsbad, CA), 1-2 µl of purified PCR product and amplification primers. Sequences were run on an ABI 3730 DNA sequencer and Mutation Surveyor V3.10 (SoftGenetics) was used for sequence analysis. Sequence analysis was performed by manually inspecting known variants and exon-exon-junctions in samples from MMR variant carriers and control individuals (2-3 control samples were run in the same experiment and at least 10 controls samples were amplified in previous analyses). The percentage of alternative/aberrant isoforms apart from the normally-spliced reference transcript (ns transcript) was evaluated in % using the “dropping factor” parameter provided by the Mutation Surveyor software, by comparing the results obtained with samples from patients carrying MMR VUS to those from control individuals devoid of germline MMR variants. The expression of a coding variant allele in the reference transcript was estimated as percentage regarding the “dropping factor” of the two nucleotides at the same position (variant versus WT) in the Sanger sequencing electropherogram. In the case of intronic spliceogenic variants, the presence of heterozygous exonic SNPs within the same amplicon might be used instead.

##

## cDNA analysis protocol with RT-PCR of FLT and TTS in MUC

cDNA generation

Lymphocytes were separated from human whole blood using LeucoSep tubes (Greiner Bio-One), cultured for 72-96 h at 37°C. RNA from PAXgene blood tubes was isolated using the manual PAXgene RNA extraction kit (PreAnalytics) following the manufacturers` protocol. RNA extraction from short term lymphocyte cell cultures in presence/absence of puromycin for 6 hours (+P at final concentration of 0.2 mg/ml) was performed following the instructions of the RNeasy RNA blood mini kit (QIAGEN), including DNase digestion [2]. RNA abundance was measured photometrically (Xpose), quality was monitored on a 1% agarose gel, and RNA integrity number (RIN) was determined (Agilent Bioanalyzer). From 1 µg total RNA cDNA was generated with the iScript Select cDNA synthesis kit (Bio-Rad) and Oligo-(dT) primer in a modified protocol [3]. Absence of genomic contamination and capability of cDNA amplification of a short fragment was tested in a PCR approach [2] (**Supplemental Table 1**).

cDNA analysis

From 1-2 µl cDNA we carried out Long-Range RT-PCR amplifications for the FLT of *MLH1*, *MSH2*, or *MSH6* with a forward primer located in the 5´ untranslated region (UTR) of the first exon, and the reverse primer in the 3`UTR of the last exon of the reference transcript [3]. The IProof High-Fidelity DNA Polymerase (Bio-Rad) was used to amplify 2.452 bp of the *MLH1* transcript (c.-148_*33 amplicon), or 2.965 bp of *MSH2* (c.-41_*121 amplicon), and PrimeSTAR GXL DNA Polymerase (TAKARA Clontech) was applied for amplification of 4.163 bp of the *MSH6* transcript (c.-37_*43 amplicon).

We additionally performed TTS analyses amplifying transcripts within specific regions of interest (**Supplemental Table 2**). In *MSH2* we utilized primers in exon 1 and 3 to obtain a c.-41_615 amplicon, in exon 5 and 8 for amplicon c.906_1357, and in exon 13 and 15 for amplicon c.2172_2591. Primers in *MLH1* exon 10 and 14 amplified c.805_1620, in exon 11 and 17 amplified c.981_1949 using Ampli-Taq Gold (Thermofisher) [2]. The *MSH6* transcript was amplified with primers in exon 3 and 5 to obtain c.516_3252, primers in exon 1 and 4 amplified c.-37_728, and primers in exon 3 and 10 amplified c.516_4128 using PrimeSTAR GXL DNA Polymerase (TAKARA Clontech).

The PCR products were visualized on a 1% agarose gel electrophoresis and purified with Exo-SAP kit (USB). For direct Sanger sequencing of the amplified PCR fragments, we used Big Dye v1.1 (Applied Biosystems) and several internal primers as described [3]. Sequences were run on an ABI PRISM 3100-Avant or ABI 3730 Genetic Analyzer. Sequence analysis was performed on the FLT by manually inspecting known variants and exon-exon-junctions in samples from MMR variant carriers and control individuals (2 control samples were run in the same experiment and at least 10 controls samples were amplified in previous analyses). Sequences different from the wild-type (WT) were identified and measured in peak heights using Sequence Scanner v2.0 (Applied Biosystems)/Mutation Surveyor 3.1 (SoftGenetics) to calculate their intensity in % in reference to the level of ns transcripts rounded to intervals of 5, and reporting start set at 5%. For an automated analysis of the sequences the Mutation Surveyor V3.10 (SoftGenetics) software was additionally used.

**Interpretation of RT-PCR splicing analyses**

The percentages of the allelic expression of variant versus WT sequences in the normally-spliced (ns) reference transcript and levels of aberrant splicing (termed aberrant transcripts/isoforms) were assessed as detailed above. Of note, all transcripts deviating from the normally spliced reference transcript (ns transcript) in regards of splicing were designated as “aberrant transcripts”. Since the underlying reason for presence of aberrant transcripts can either be a variant-induced splicing defect or alternative splicing, these two scenarios must be discriminated by comparing patient-derived data with that from control samples. The effect of the variants investigated was assigned as “splicing defect” when aberrant splicing was found with high intensity. A cut-off for splicing defect in cDNA+P was defined as >30% based on the result of the FLT cDNA analysis of class 4/5 variants affecting splicing, showing aberrant transcripts at 30–50% intensity in cDNA+P [3]. In some cases, in order to evaluate whether the splicing effect was total or partial, bands corresponding to the ns or aberrant transcripts were excised from agarose gels. DNA was extracted using GFX PCR DNA and Gel Band Purification kit (GE Healthcare, Chicago, IL, USA) and sequenced to assess mono or biallelic expression. The relative expression of the variant-carrying allele (peak detected in cDNA Sanger sequencing) was compared to Sanger sequencing of genomic DNA. When the absence of ns transcript was proven for the variant allele, the effect was assigned as “complete splicing defect”. On the other hand, variants were designated as “splice-neutral” when no effect on splicing was observed in terms of aberrant transcripts in association to the variant allele.

## Minigene reporter splicing assay in URO

The cell-based minigene splicing assay compared the splicing patterns of WT and mutant reporter minigenes, as previously described [4, 5]. Briefly, genomic fragments containing the exon of interest and ~100-150 nucleotides of flanking intronic sequences, were PCR–amplified by using patient gDNA as template and the primers described in **Supplemental Table 3**. Then, the amplicons were introduced into the intron of the pCAS2 minigene vector [6] previously digested with BamHI and MluI in order to prepare three-exon or four-exon pCAS2-MMR minigenes. All constructs were sequenced to exclude unwanted mutations in the inserted fragment. WT and mutant minigenes were transfected in parallel into HeLa cells grown at ~70% confluence by using the FuGENE 6 transfection reagent (Roche Applied Science). HeLa cells were cultivated in Dulbecco’s modified Eagle medium (Life Technologies) supplemented with 10% fetal calf serum in a 5% CO2 atmosphere at 37°C. Twenty-four hours later, total RNA was extracted using the NucleoSpin RNA II kit (Macherey Nagel) according to the manufacturers’ instructions, except for the study of *MSH2* c.2459-12A>G, where RNA was extracted with the TriPure Isolation Reagent (Roche) and treated with DNAse I (AMP D1, Roche). In most cases, minigenes’ transcripts were analysed by RT-PCR in a 25 μl reaction volume by using 200 ng total RNA, the OneStep RT-PCR kit (Qiagen) and minigene specific primers (**Supplemental Table 3**). For *MSH2* c.2459-12A>G, 1 µg RNA was reversed transcribed with Superscript II Reverse Transcriptase (Invitrogen) in the presence of 0.5 µg oligo d(T)_18_. Then, cDNA was amplified using Taq Polymerase (ABgene) and minigene specific primers (**Supplemental Table 3**). RT-PCR products were resolved by electrophoresis on 2.0-2.5 % agarose gels and visualised using the Gel Doc XR image acquisition system (Bio-RAD). RT-PCR products were gel-excised, purified and Sanger sequenced with the RT-PCR primers.

When indicated, splicing efficiency was calculated by computing the fraction of spliced product signal over the sum of spliced and unspliced signals, either by quantitating the RT-PCR products separated on ethidium bromide-stained gels or on an automated sequencer after performing the RT-PCR reactions with a fluorescent forward primer.

## Bioinformatic predictions

Splicing defect predictions of MMR variants were obtained by using SpliceSiteFinder-like (SSF-L, http://www.interactive-biosoftware.com) and MaxEntScan (MES, http://genes.mit.edu/burgelab/maxent/Xmaxentscan_scoreseq.html; Maximum Entropy Model) algorithms, via the integrated software tool Alamut Visual software 2.10 (<http://www.interactive-biosoftware.com>) and according to recommended thresholds [7, 8], and SpliceAI ([https://spliceailookup.broadinstitute.org/](https://eur03.safelinks.protection.outlook.com/?url=https%3A%2F%2Fspliceailookup.broadinstitute.org%2F&data=04%7C01%7Cmpineda%40iconcologia.net%7Cc90e8d2d274142942f5108d9ec04fb3a%7C3b9427dcd30e43bc8c06ff7253676fec%7C1%7C0%7C637800329966432277%7CUnknown%7CTWFpbGZsb3d8eyJWIjoiMC4wLjAwMDAiLCJQIjoiV2luMzIiLCJBTiI6Ik1haWwiLCJXVCI6Mn0%3D%7C3000&sdata=qfoRoriiI1ppNNkB2kDlbzo6WG7F6D1C4cUWZCVG8rg%3D&reserved=0)) according to default thresholds. Protein function predictions were obtained from HCI Cancer Susceptibility Genes Prior Probabilities of Pathogenicity (http://priors.hci.utah.edu/PRIORS/index.php).

**SUPPLEMENTAL REFERENCES**

1. Colombo M, Blok MJ, Whiley P, Santamarina M, Gutierrez-Enriquez S, Romero A et al. Comprehensive annotation of splice junctions supports pervasive alternative splicing at the BRCA1 locus: a report from the ENIGMA consortium. Hum Mol Genet. 2014;23(14):3666-80.

2. Morak M, Koehler U, Schackert HK, Steinke V, Royer-Pokora B, Schulmann K et al. Biallelic MLH1 SNP cDNA expression or constitutional promoter methylation can hide genomic rearrangements causing Lynch syndrome. J Med Genet. 2011;48(8):513-9.

3. Morak M, Schaefer K, Steinke-Lange V, Koehler U, Keinath S, Massdorf T et al. Full-length transcript amplification and sequencing as universal method to test mRNA integrity and biallelic expression in mismatch repair genes. Eur J Hum Genet. 2019;27(12):1808-20.

4. Gaildrat P, Killian A, Martins A, Tournier I, Frebourg T, Tosi M. Use of splicing reporter minigene assay to evaluate the effect on splicing of unclassified genetic variants. Methods Mol Biol. 2010;653:249-57.

5. Tournier I, Vezain M, Martins A, Charbonnier F, Baert-Desurmont S, Olschwang S et al. A large fraction of unclassified variants of the mismatch repair genes MLH1 and MSH2 is associated with splicing defects. Hum Mutat. 2008;29(12):1412-24.

6. Soukarieh O, Gaildrat P, Hamieh M, Drouet A, Baert-Desurmont S, Frebourg T et al. Exonic Splicing Mutations Are More Prevalent than Currently Estimated and Can Be Predicted by Using In Silico Tools. PLoS Genet. 2016;12(1):e1005756.

7. Houdayer C. In silico prediction of splice-affecting nucleotide variants. Methods in Molecular Biology (Clifton, NJ). 2011;760:269-81.

8. Houdayer C, Caux-Moncoutier V, Krieger S, Barrois M, Bonnet F, Bourdon V et al. Guidelines for splicing analysis in molecular diagnosis derived from a set of 327 combined in silico/in vitro studies on BRCA1 and BRCA2 variants. Human mutation. 2012;33(8):1228-38.
