# Supplementary material for: Splicing analyses for variants in MMR genes: best practice recommendations from the European Mismatch Repair Working Group
Source: Eur J Hum Genet. 2022 Jun 9;30(9):1051–9. doi: 10.1038/s41431-022-01106-w (PMC9437034; doi:10.1038/s41431-022-01106-w)
Supplement: Supplementary file 2 — Supplemental figures' legends [file 41431_2022_1106_MOESM2_ESM.docx]

**SUPPLEMENTAL FIGURES’ LEGENDS**

**Supplemental Figure S1:** Schematic representation of the experimental approaches for evaluating the effect of MMR variants on mRNA splicing used in this study.

**Supplemental Figure S2:** Sanger sequencing electropherograms from carriers and controls obtained from the experimental approaches used in this work.

**Supplemental Figure S3:** Schematic representation of the SpliceSiteFinder-like and MaxEntScan algorithms splicing predictions, obtained via the integrated software tool Alamut Visual software 2.10.
