# Supplementary figures and images for: Splicing analyses for variants in MMR genes: best practice recommendations from the European Mismatch Repair Working Group
Source: Eur J Hum Genet. 2022 Jun 9;30(9):1051–9. doi: 10.1038/s41431-022-01106-w (PMC9437034; doi:10.1038/s41431-022-01106-w)

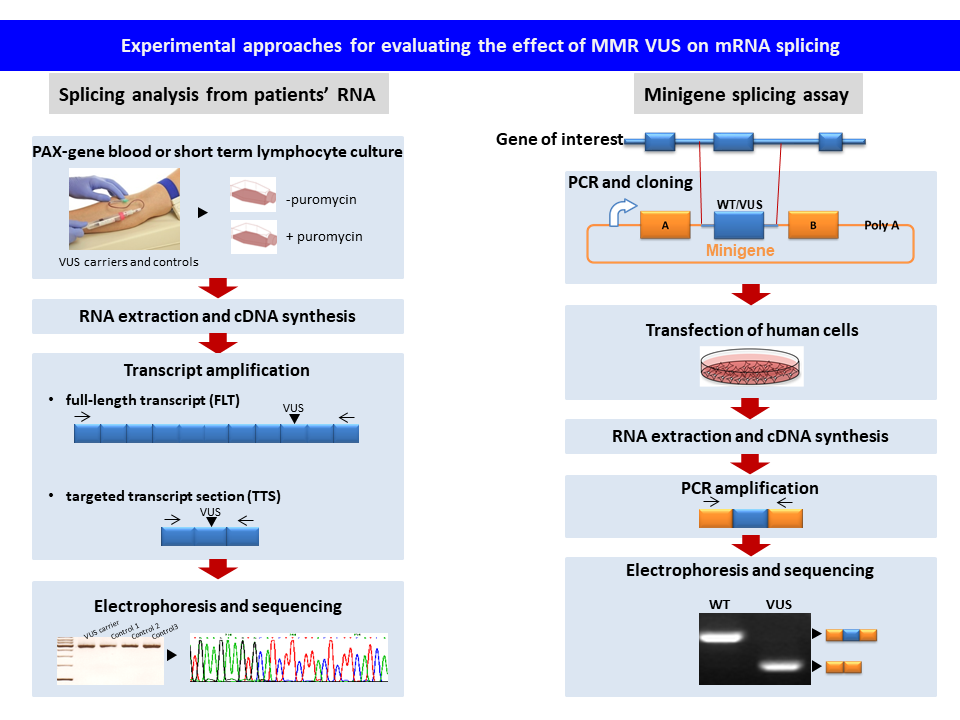

Supplement: Supplementary file 3 — Supplemental Figure 1_Schematic representation of the experimental approaches for evaluating the effect of MMR VUS on mRNA splicing used in this study [file 41431_2022_1106_MOESM3_ESM.tif]
