# Supplementary material for: Splicing analyses for variants in MMR genes: best practice recommendations from the European Mismatch Repair Working Group
Source: Eur J Hum Genet. 2022 Jun 9;30(9):1051–9. doi: 10.1038/s41431-022-01106-w (PMC9437034; doi:10.1038/s41431-022-01106-w)
Supplement: Supplementary file 4 — Supplemental Figure 2_Sanger sequencing electropherograms from carriers and controls obtained from the experimental approaches used in this work. [file 41431_2022_1106_MOESM4_ESM.pdf]

MSH2 c.211G>C

Gene and variant	*MSH2* c.211G>C (exon 1)
Experimental approach	TTS
Laboratory	BCN
Design	E1-E4
Result of the splicing analysis	r.195_211del (p.Tyr66Serfs*10) = Δ1q(17)

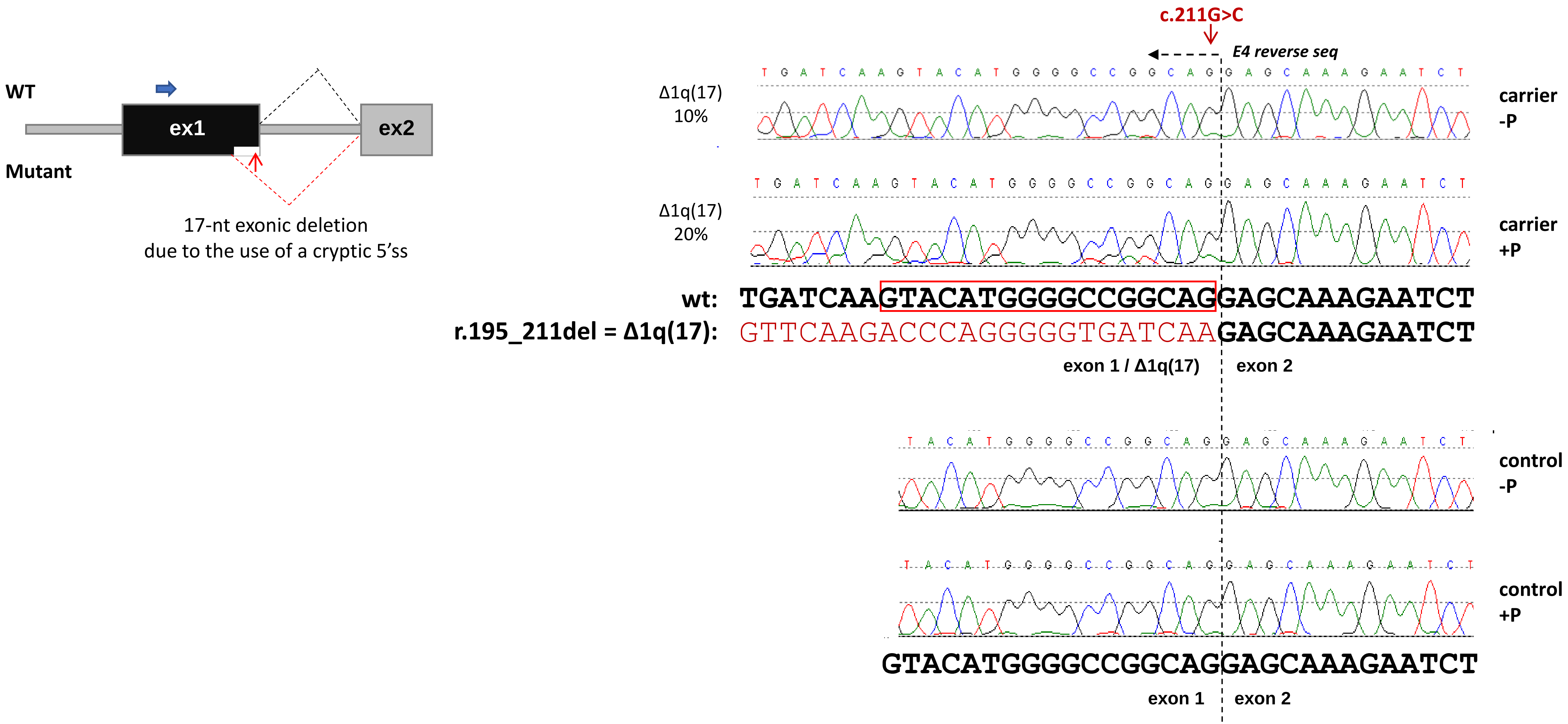

Gene and variant	*MSH2* c.211G>C (exon 1)
Experimental approach	FLT
Laboratory	MUC
Design	E1-E16
Result of the splicing analysis	r.195_211del (p.Tyr66Serfs*10) = Δ1q(17)<br>r.-16_211del (p.?) = Δ1q(227)

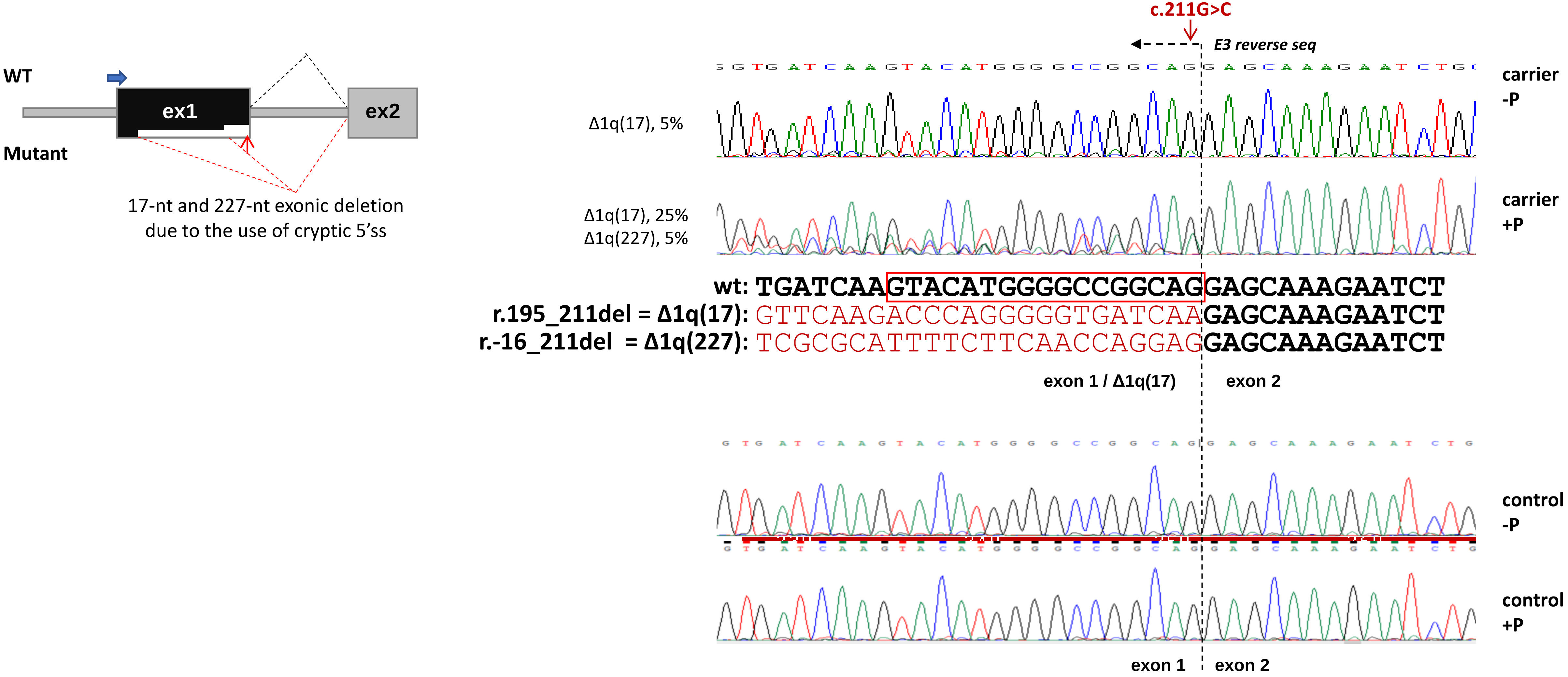

Gene and variant	*MSH2* c.211G>C (exon 1)
Experimental approach	TTS
Laboratory	MUC
Design	E1-E3
Result of the splicing analysis	Not analysable, PCR-bias: preferential amplification of Δ1q(227) aberrant transcript in sample (100%) and controls (10-20%)

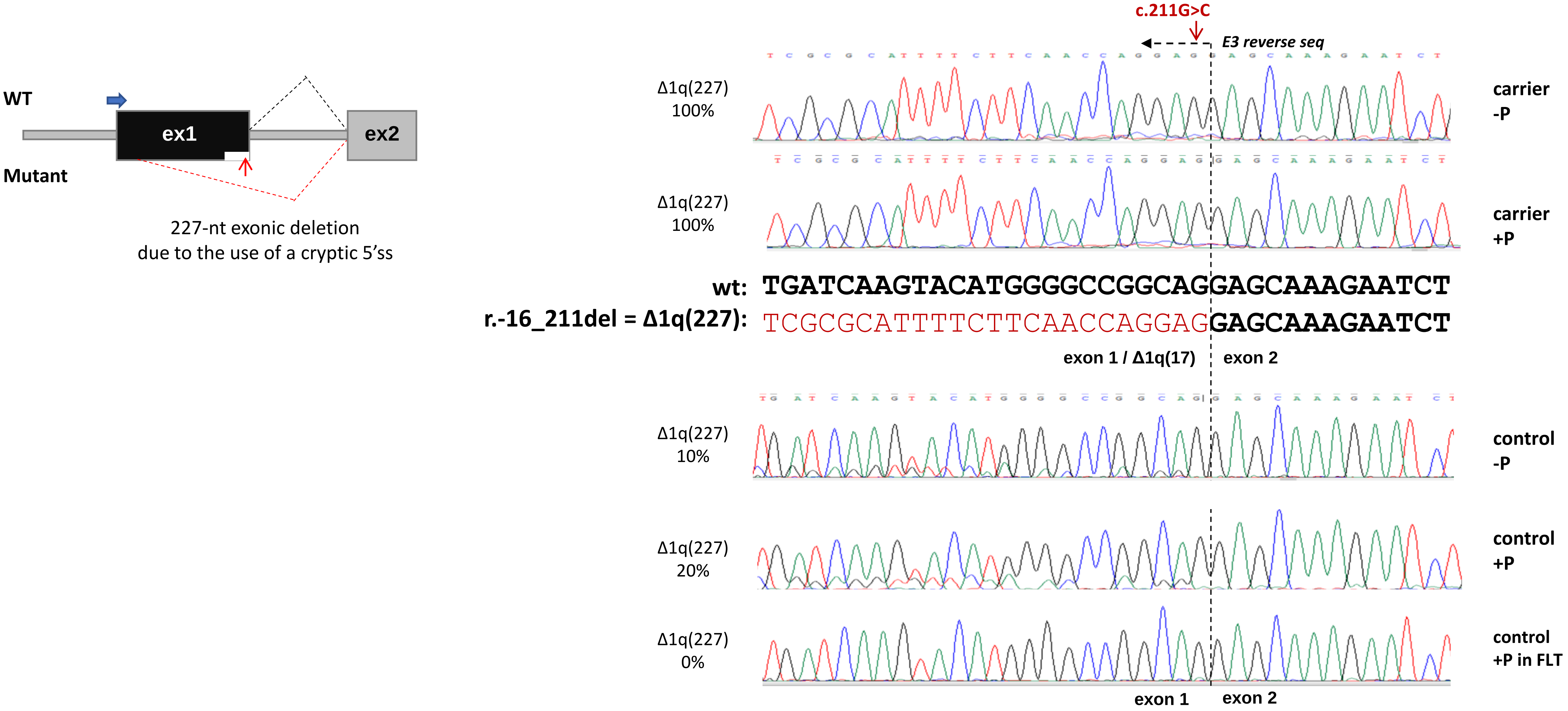

MSH2 c.1276G>A

Gene and variant	*MSH2* c.1276G>A (exon 7)
Experimental approach	TTS
Laboratory	BCN
Design	E6-E13
Result of the splicing analysis	r.1229_1276del48 (p.Ile411_Gly426del) = Δ7q(48)

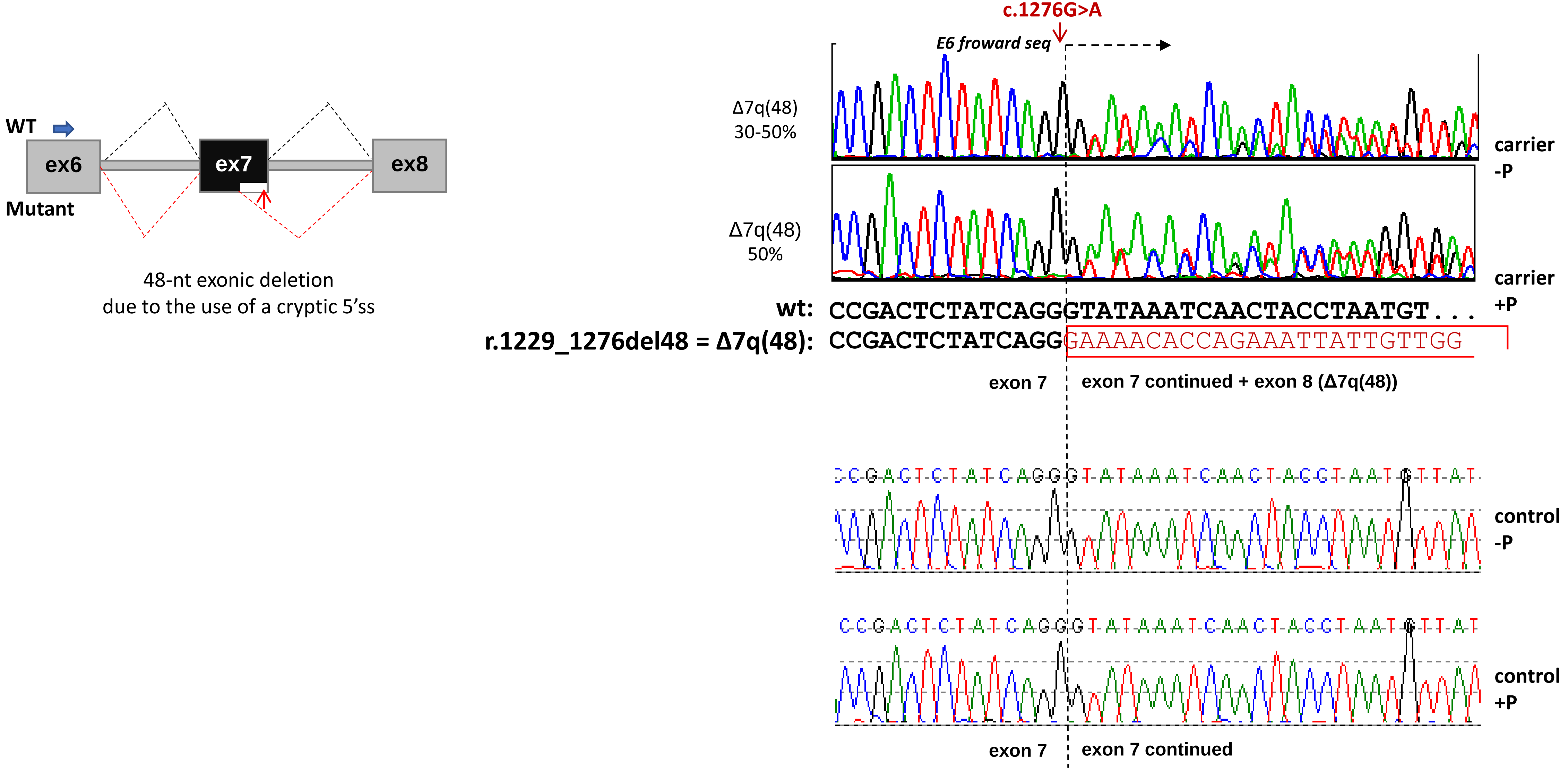

Gene and variant	*MSH2* c.1276G>A (exon 7)
Experimental approach	FLT
Laboratory	MUC
Design	E1-E16
Result of the splicing analysis	r.1229_1276del48 (p.Ile411_Gly426del) = Δ7q(48)

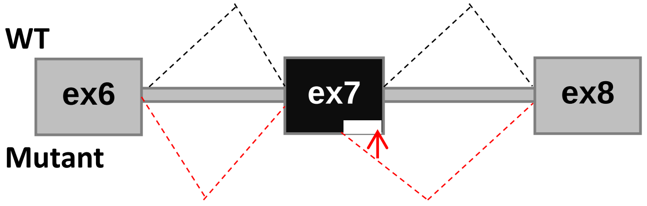

48-nt exonic deletion
due to the use of a cryptic 5’ss

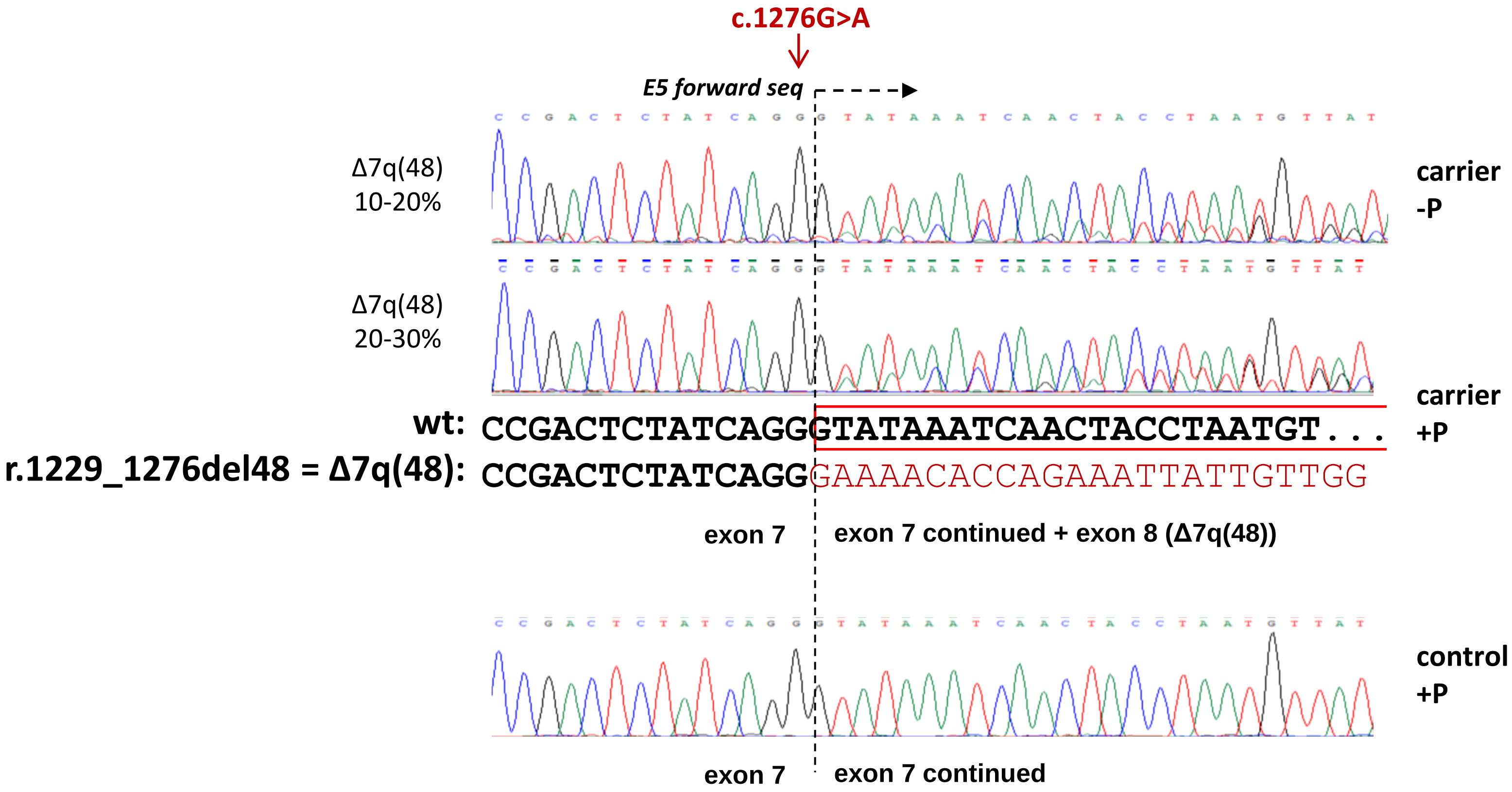

Gene and variant	*MSH2* c.1276G>A (exon 7)
Experimental approach	TTS
Laboratory	MUC
Design	E5-E8
Result of the splicing analysis	r.1229_1276del48 (p.Ile411_Gly426del) = Δ7q(48)

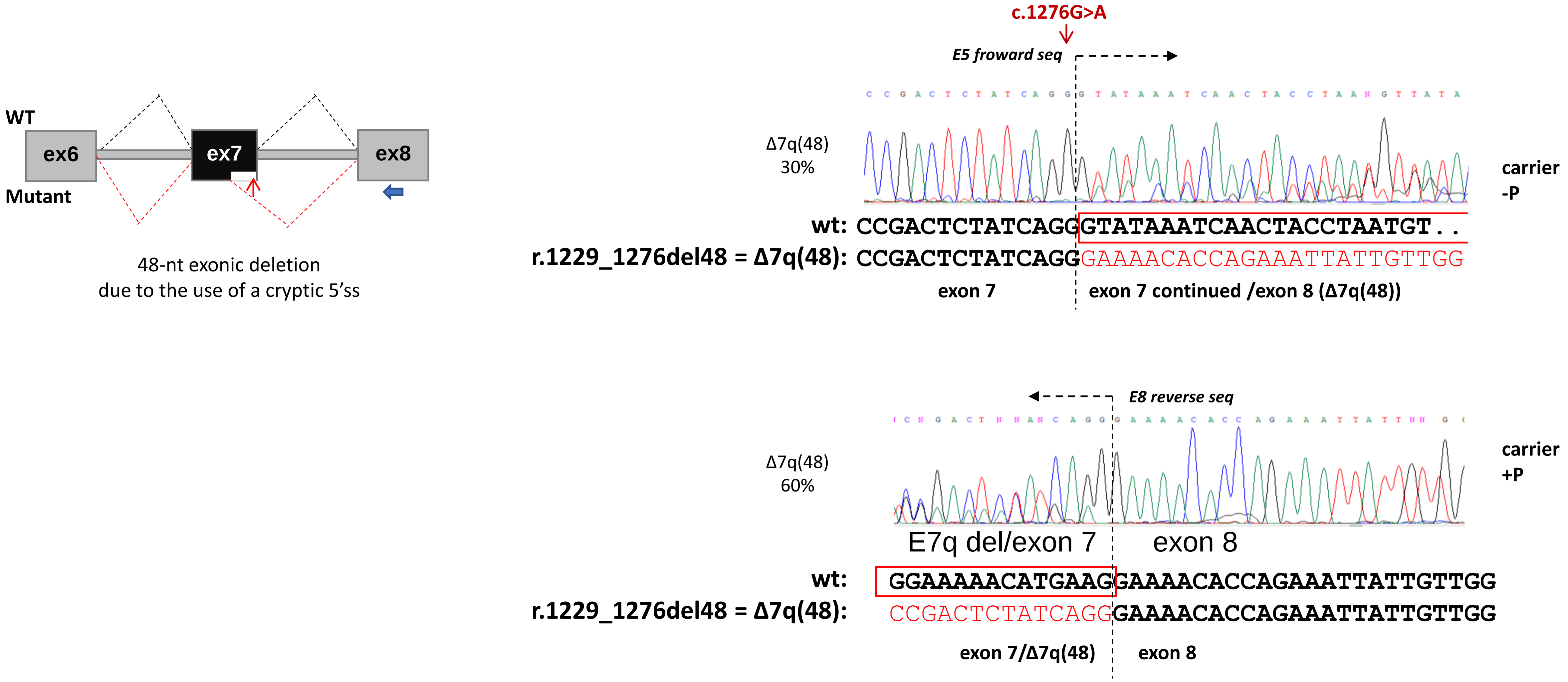

Gene and variant	*MSH2* c.1276G>A (exon 7)
Experimental approach	Minigene
Laboratory	URO
Result of the splicing analysis	r.1229_1277del (p.Ile411_Gly426del) = Δ7q(48)

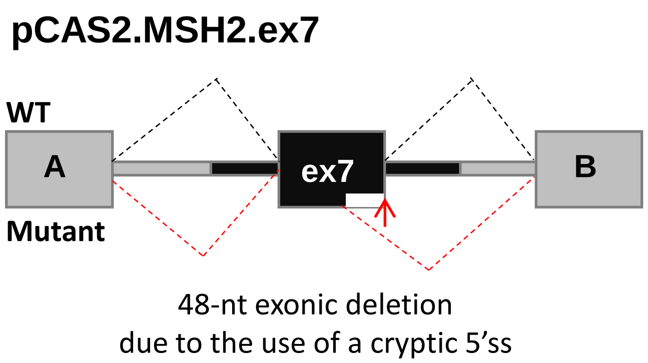

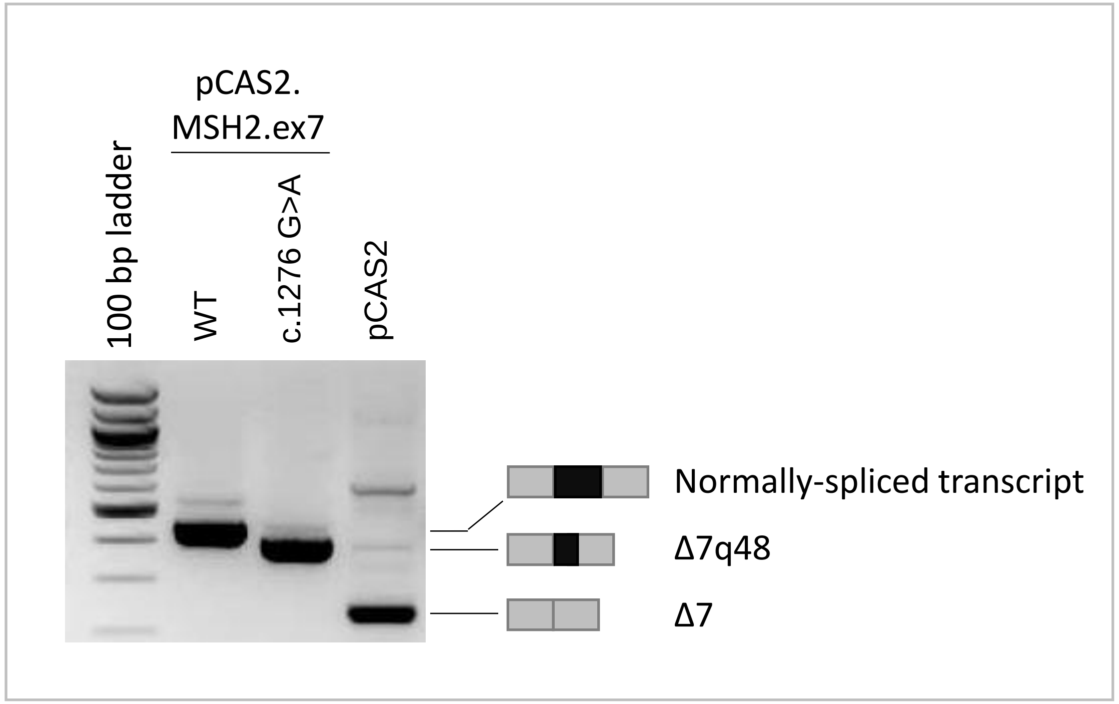

MSH2 c.2459-12A>G

Gene and variant	*MSH2* c.2459-12A>G
Experimental approach	TTS
Laboratory	BCN
Design	E14-E16
Result of the splicing analysis	r.2458_2459insATTTCTTATAG (p.Gly820Aspfs*4) = ▼15p(11)

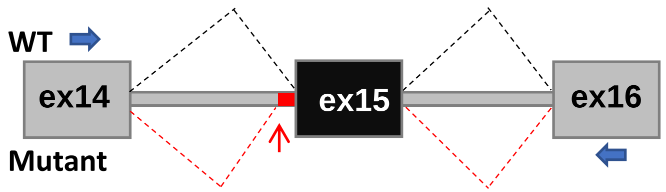

11-nt IVS14 retention
due to the use of a *de novo* 3'ss

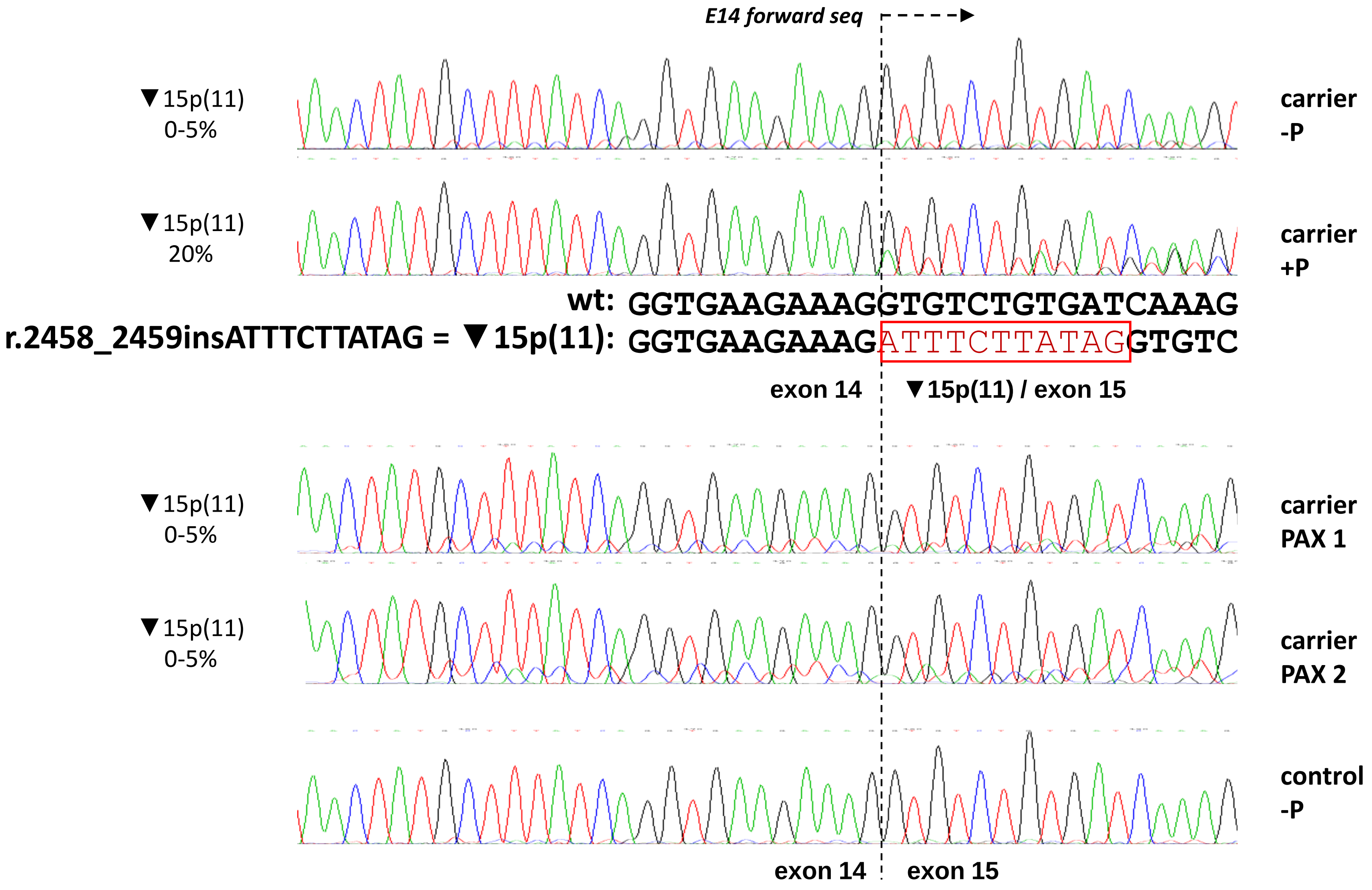

Gene and variant	*MSH2* c.2459-12A>G
Experimental approach	FLT
Laboratory	MUC
Design	E1-E16
Result of the splicing analysis	r.2458_2459insATTTCTTATAG (p.Gly820Aspfs*4) = ▼15p(11)

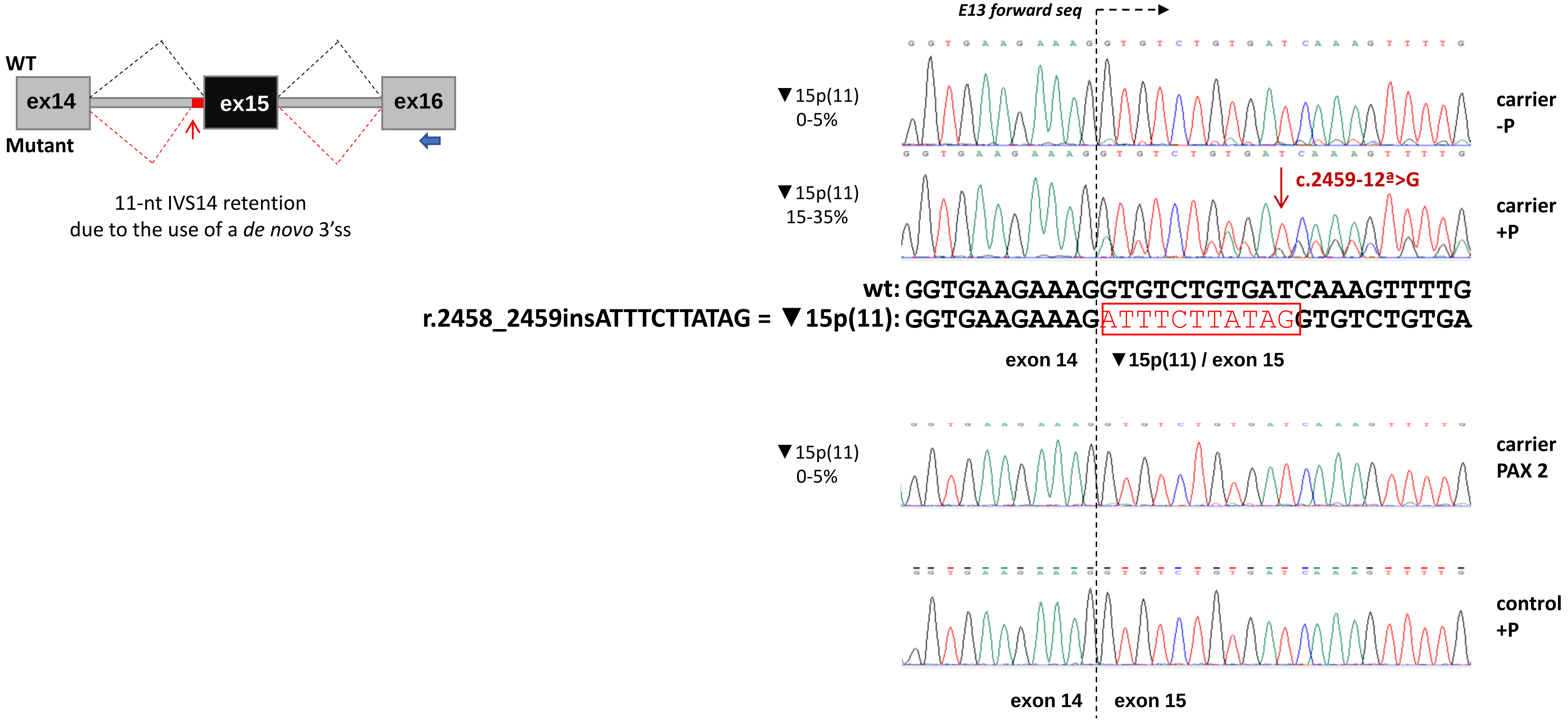

Gene and variant	*MSH2* c.2459-12A>G
Experimental approach	TTS
Laboratory	MUC
Design	E13-E15
Result of the splicing analysis	r.2458_2459insATTTCTTATAG (p.Gly820Aspfs*4) = ▼15p(11)

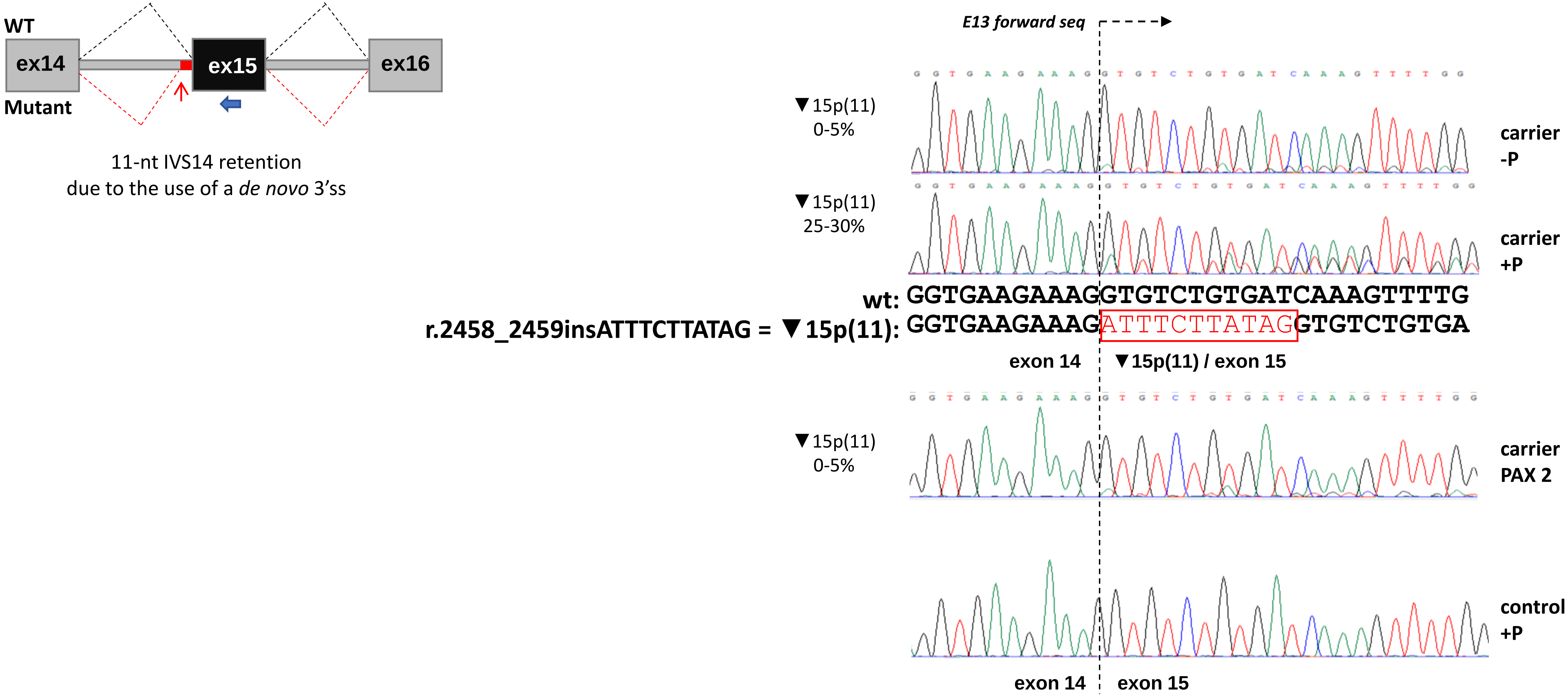

Gene and variant	*MSH2* c.2459-12A>G
Experimental approach	Minigene
Laboratory	URO
Result of the splicing analysis	▼15p(11), r.2458_2459insATTTCTTATAG (p.Gly820Aspfs*4)

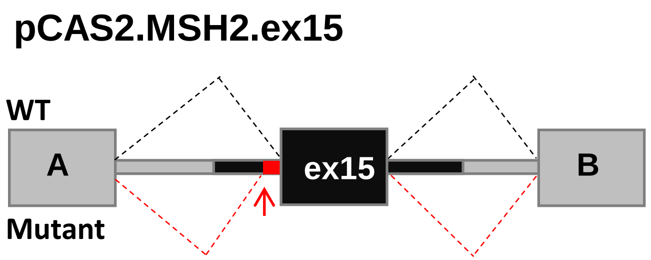

11-nt IVS14 retention
due to the use of a *de novo* 3'ss

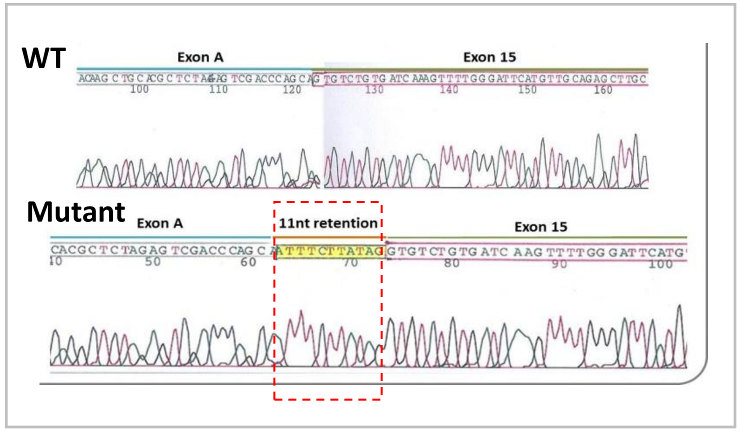

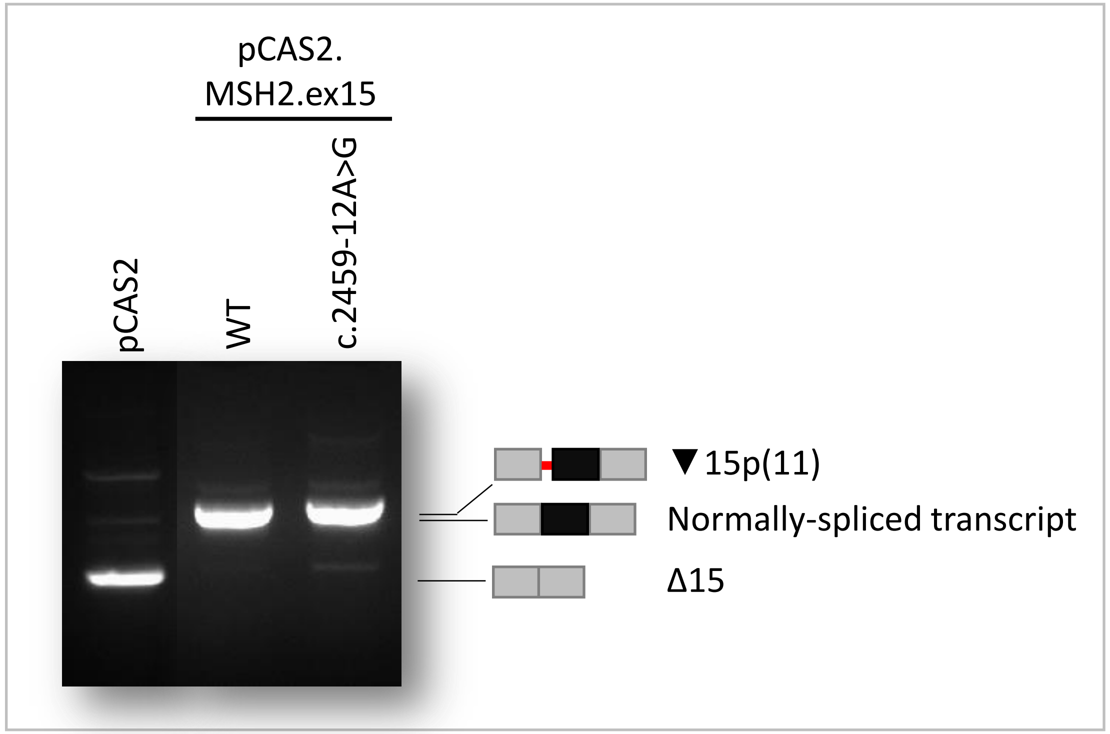

MSH6 c.1894A>G

Gene and variant	*MSH6* c.1894A>G (exon 4)
Experimental approach	TTS
Laboratory	BCN
Design	E3-E5
Result of the splicing analysis	Not analyzable due to high level of alternative splicing of Δ4 in controls

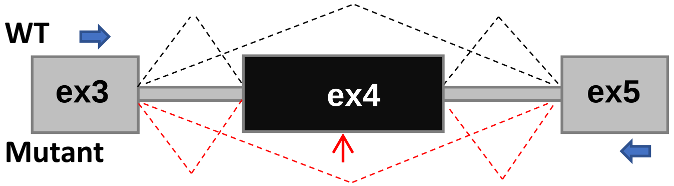

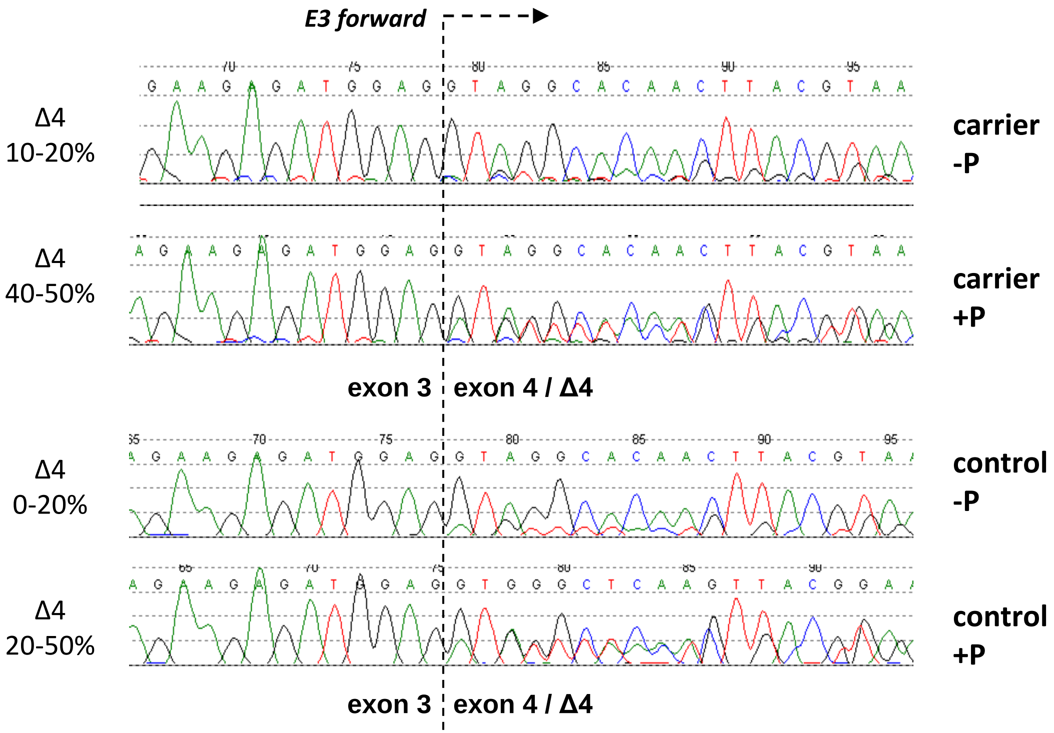

Gene and variant	*MSH6* c.1894A>G (exon 4)
Experimental approach	TTS covering the whole gene
Laboratory	MUC
Design	E1-E4 and E3-E10
Result of the splicing analysis	No effect on splicing; biallelic expression of the variant

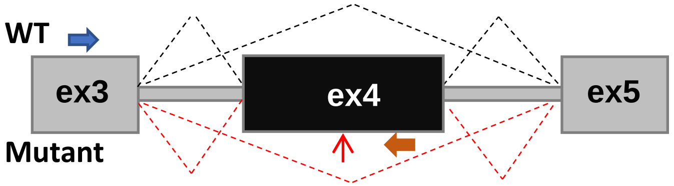

TTS E1-4

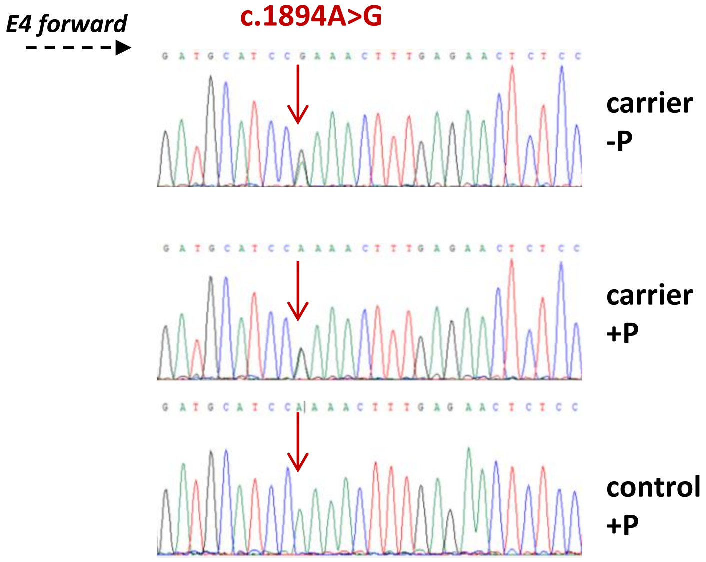

TTS E3-10

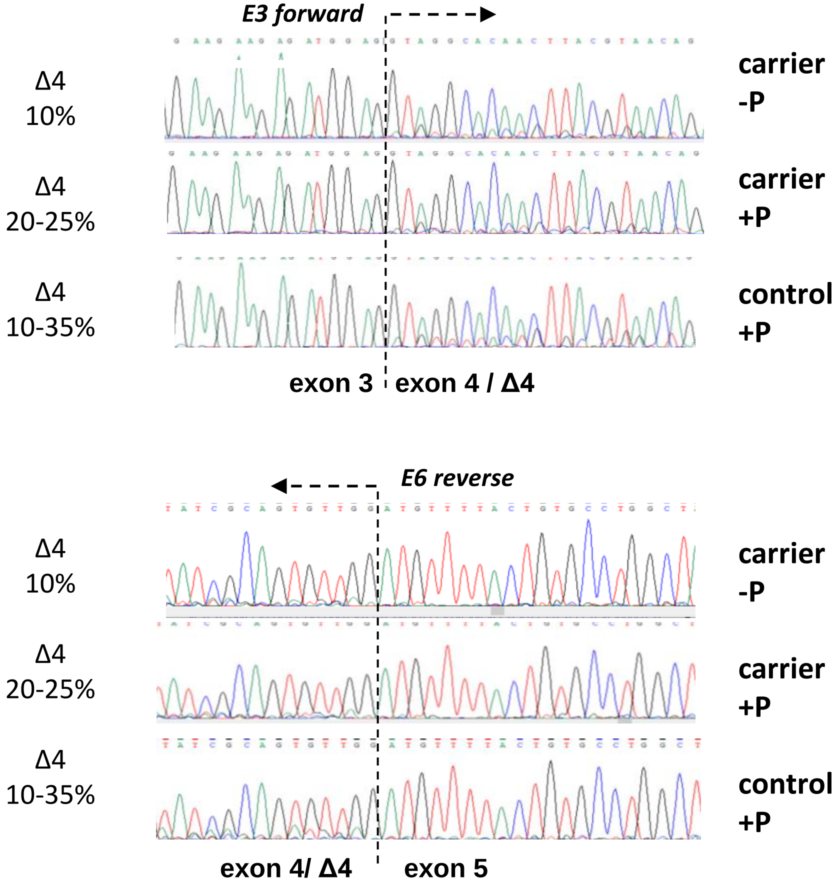

Gene and variant	*MSH6* c.1894A>G (exon 4)
Experimental approach	TTS
Laboratory	MUC
Design	E3-E5
Result of the splicing analysis	Not analyzable due to high level of alternative splicing of Δ4 in controls

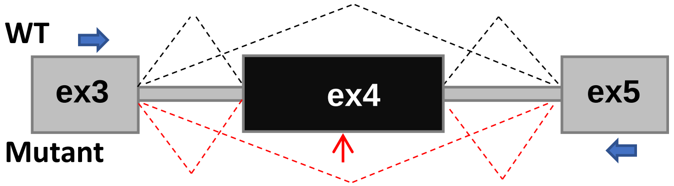

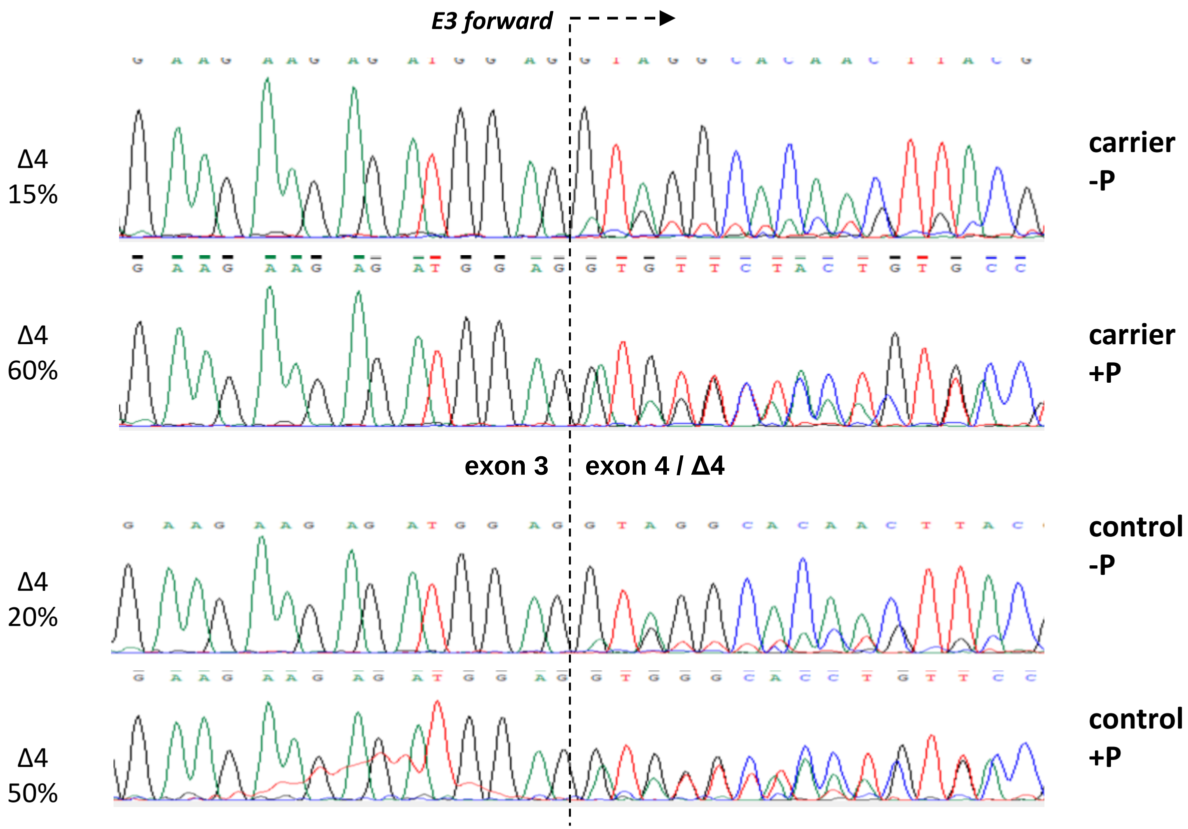

MLH1 c.1039-2A>T

Gene and variant	*MLH1* c.1039-2A>T (IVS11)
Experimental approach	TTS
Laboratory	BCN
Design	E11-E15
Result of the splicing analysis	r.1039_1409del (p.Thr347Lysfs*8) = Δ12

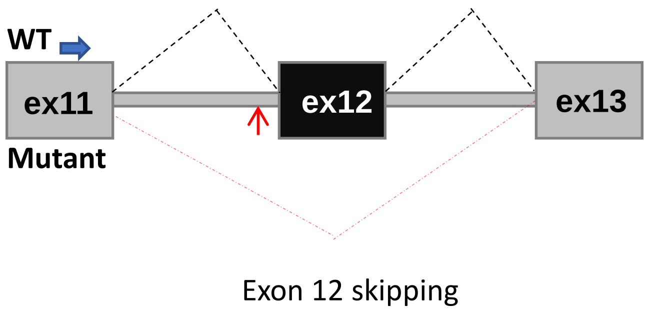

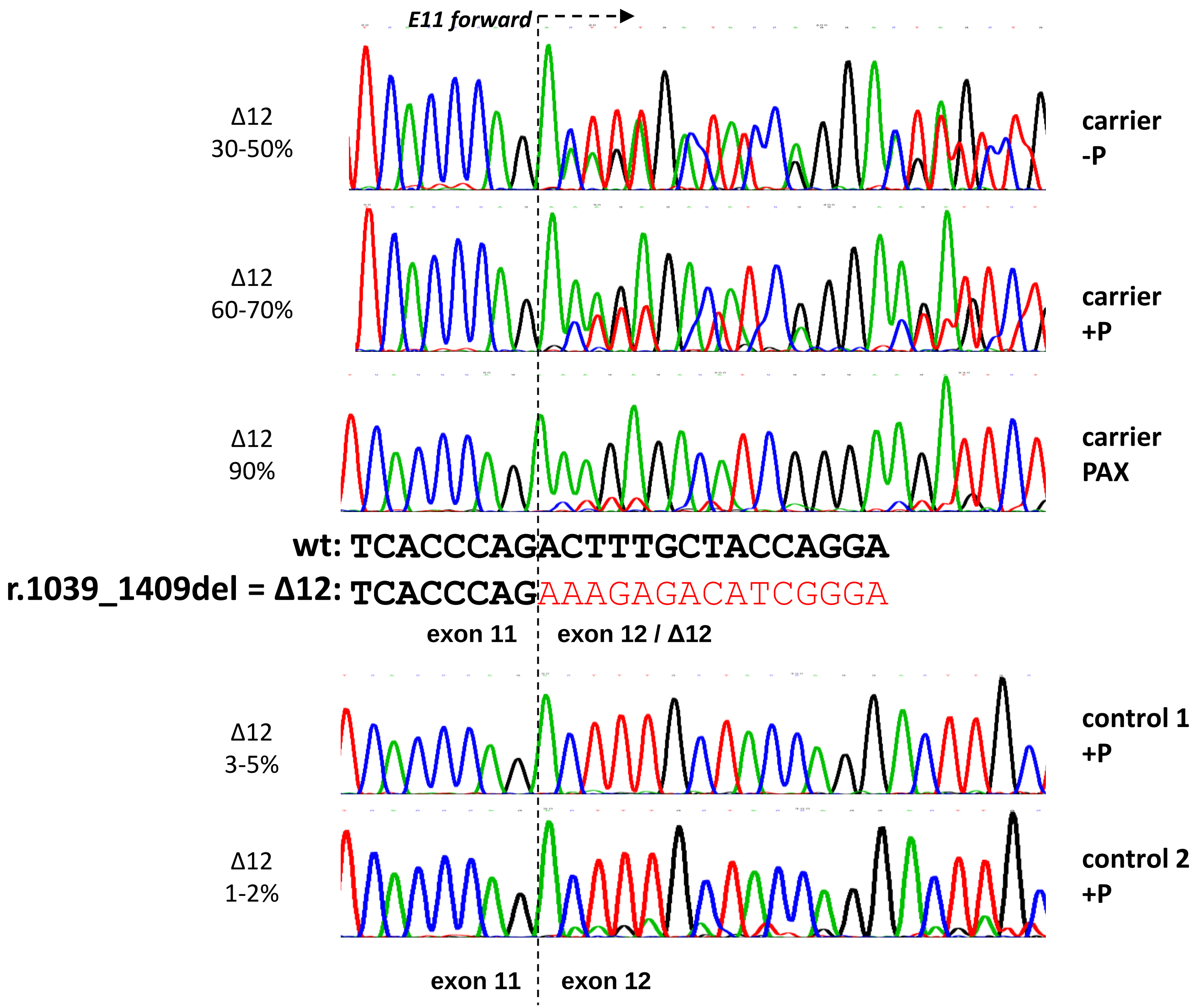

Gene and variant	*MLH1* c.1039-2A>T (IVS11)
Experimental approach	FLT
Laboratory	MUC
Design	E1-E19
Result of the splicing analysis	r.1039_1409del (p.Thr347Lysfs*8) = Δ12<br>r.678_1409del (p.Glu227_Arg470del) = Δ9-12<br>r.885_1409del (p.Ser295_Pro469del) = Δ11-12<br>r.1039_1051del (p.Thr347Aspfs*16) = Δ12p(13)

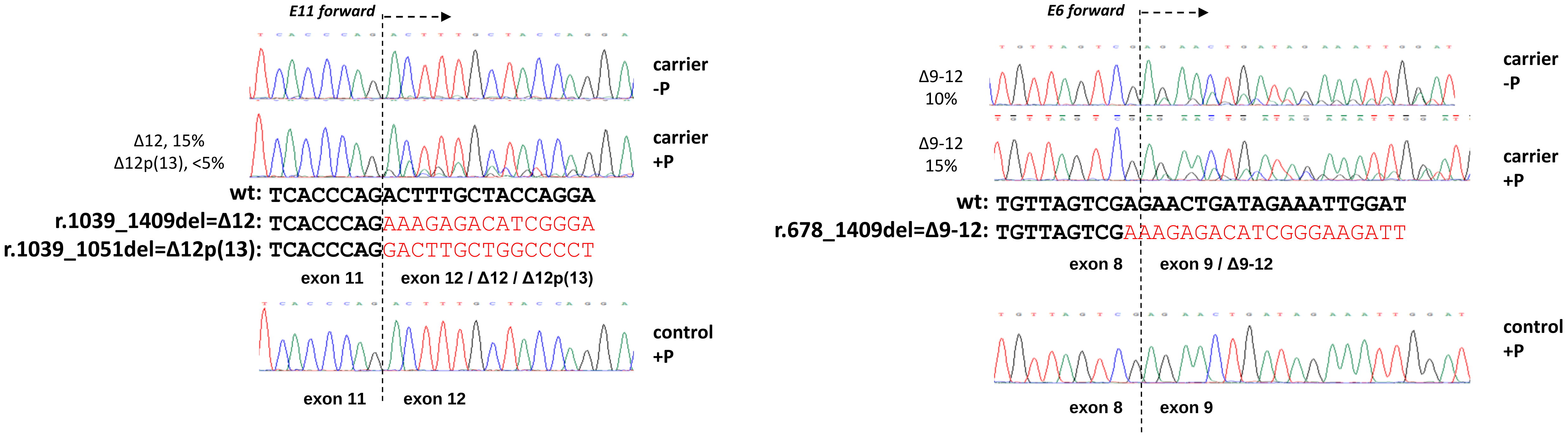

Gene and variant	*MLH1* c.1039-2A>T (IVS11)
Experimental approach	TTS
Laboratory	MUC
Design	E10-E14
Result of the splicing analysis	r.1039_1409del (p.Thr347Lysfs*8) = Δ12

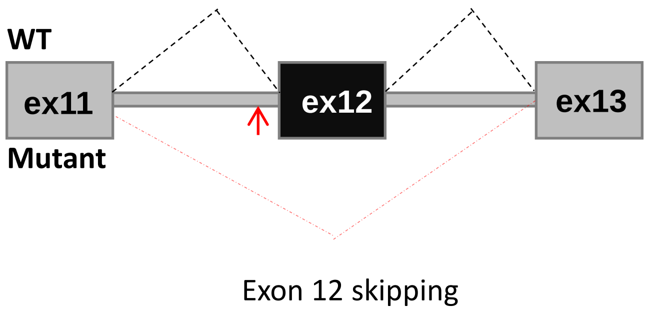

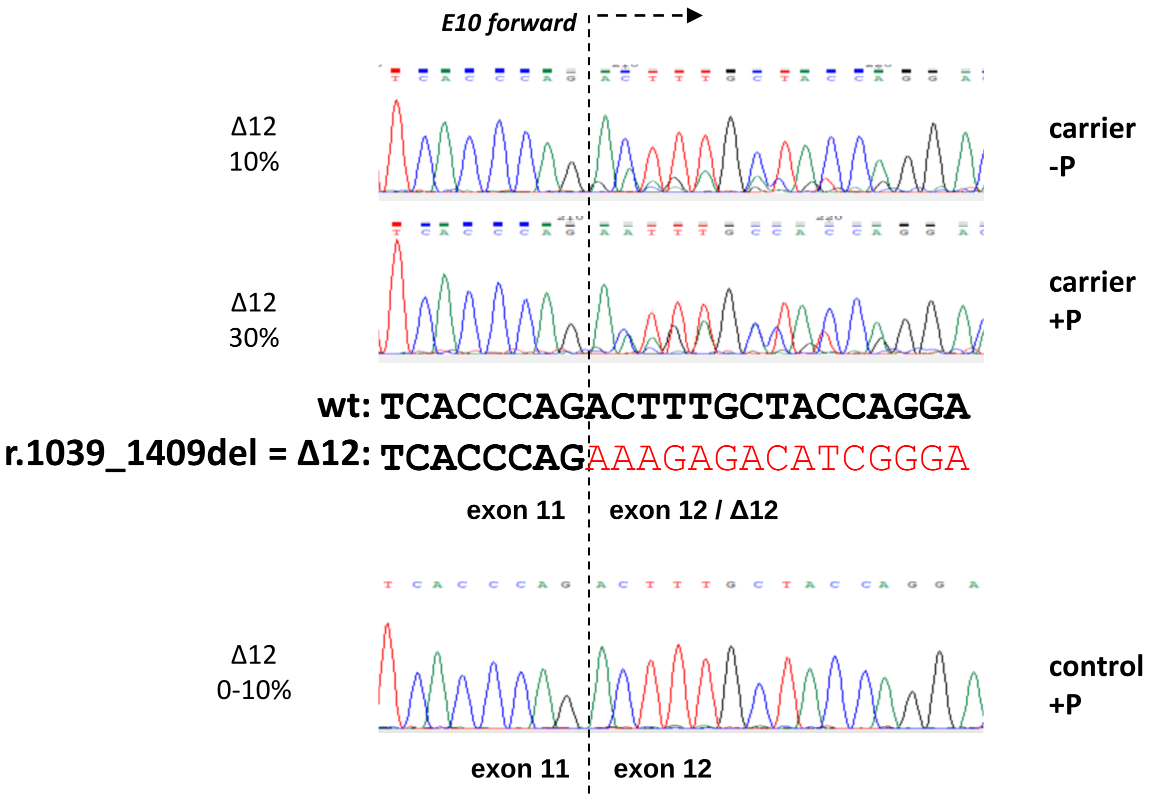

Gene and variant	*MLH1* c.1039-2A>T (IVS11)
Experimental approach	Minigene
Laboratory	URO
Result of the splicing analysis	r.1039_1051del p.(Thr347Aspfs*16) = Δ12p(13) and r.1039_1409del, p.(Thr347Lysfs*8) = Δ12

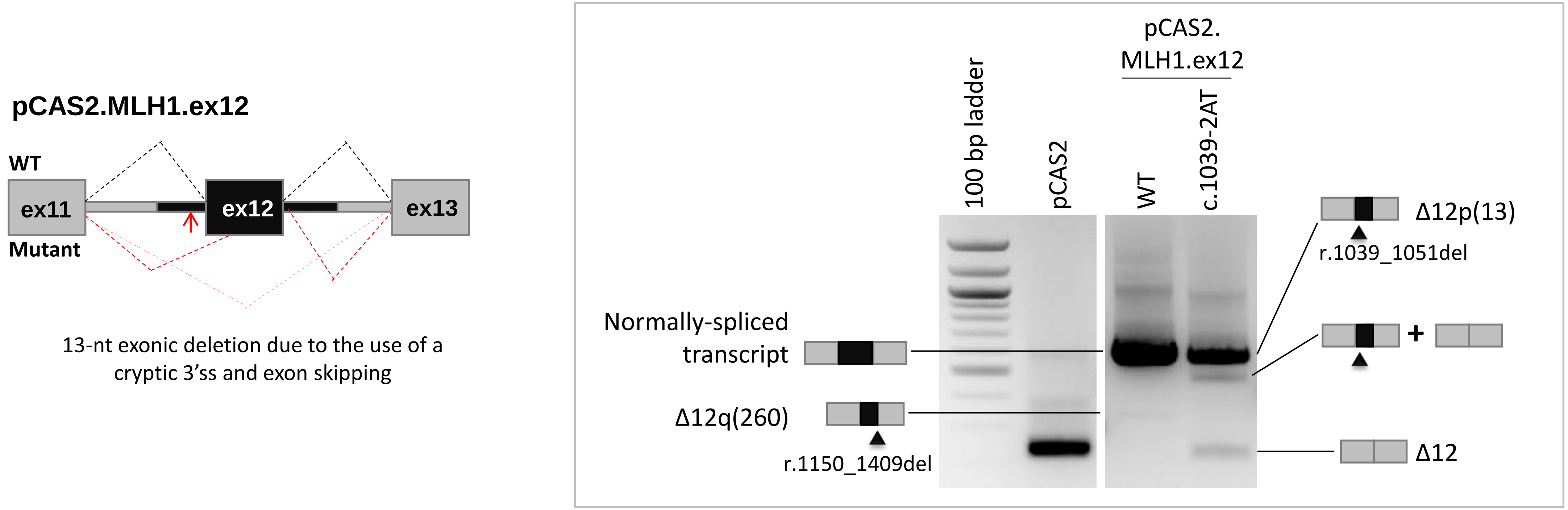

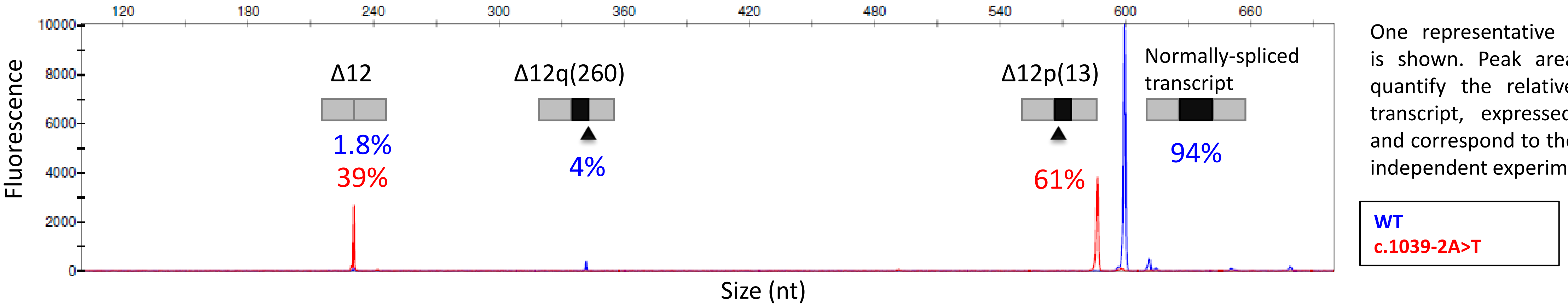

One representative electropherogram is shown. Peak areas were used to quantify the relative levels of each transcript, expressed as percentage, and correspond to the average of three independent experiments

MLH1 c.1217G>A co-occurring with c.1989+3dup

Gene and variant	*MLH1* c.1217G>A co-occurring with c.1989+3dup
Experimental approach	TTS
Laboratory	BCN
Design	E11-E15
Result of the splicing analysis	No effect on splicing (r.1217G>A); biallelic expression of the variant

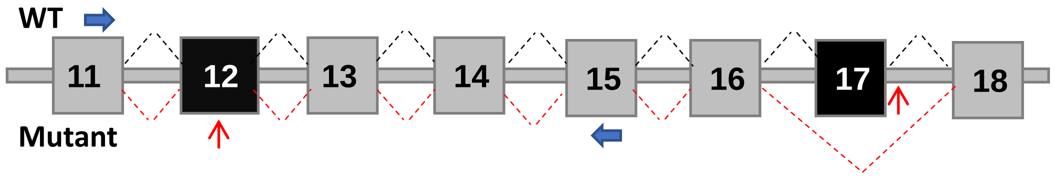

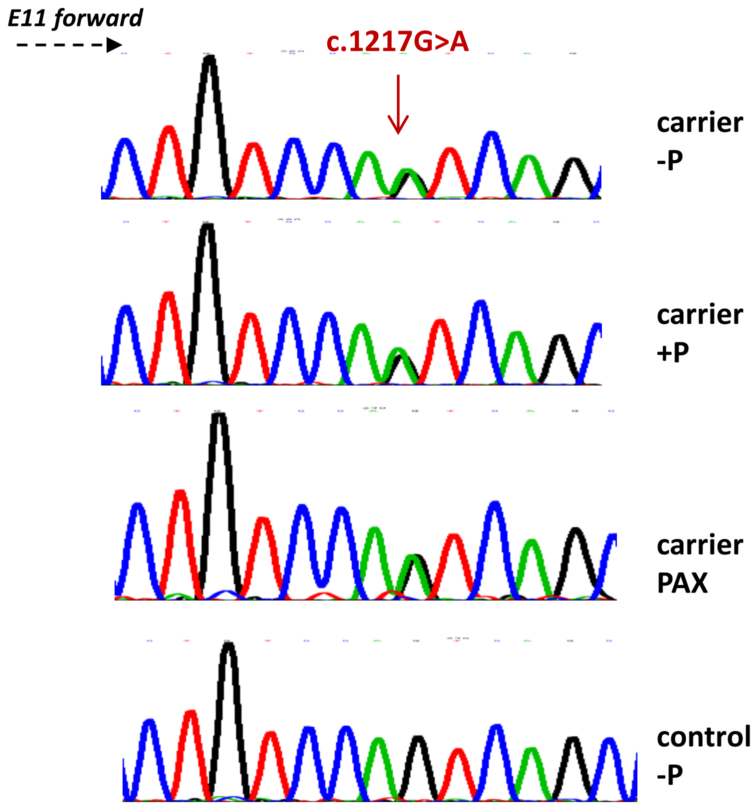

Gene and variant	*MLH1* c.1217G>A co-occurring with c.1989+3dup
Experimental approach	TTS
Laboratory	MUC
Design	E11-E17
Result of the splicing analysis	No effect on splicing; monoallelic expression of the variant in exon 12

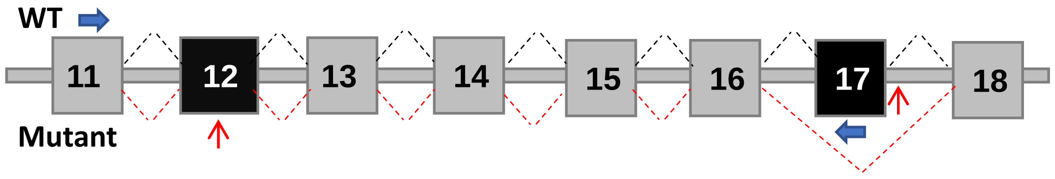

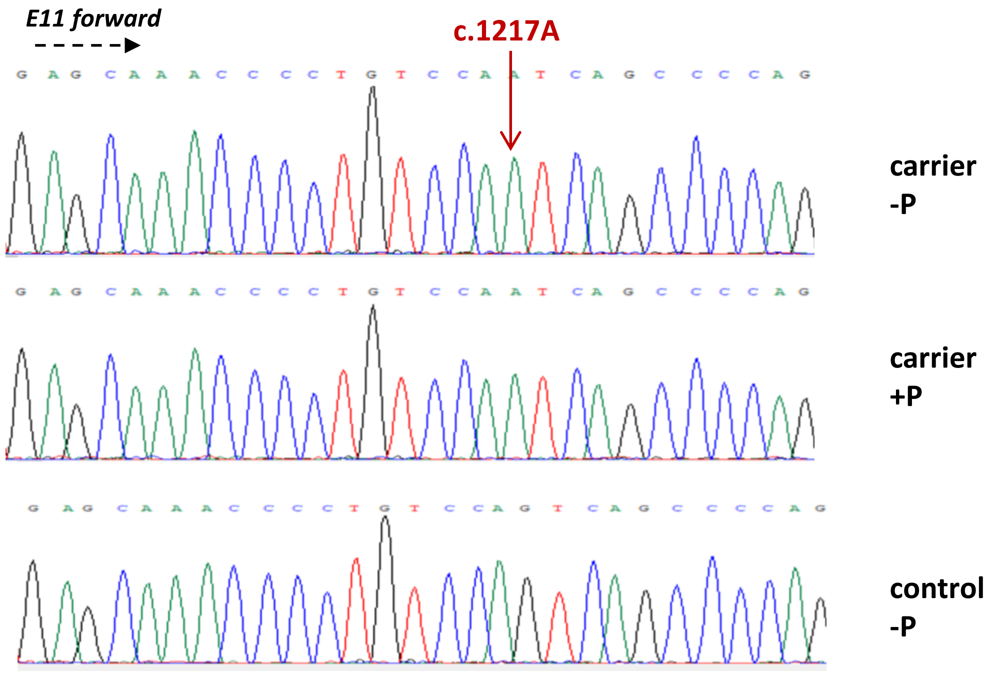

Gene and variant	*MLH1* c.1217G>A co-occurring with c.1989+3dup
Experimental approach	TTS
Laboratory	BCN
Design	E15-E19
Result of the splicing analysis	r.1897_1989del (p.Glu633_Glu663del) = Δ17

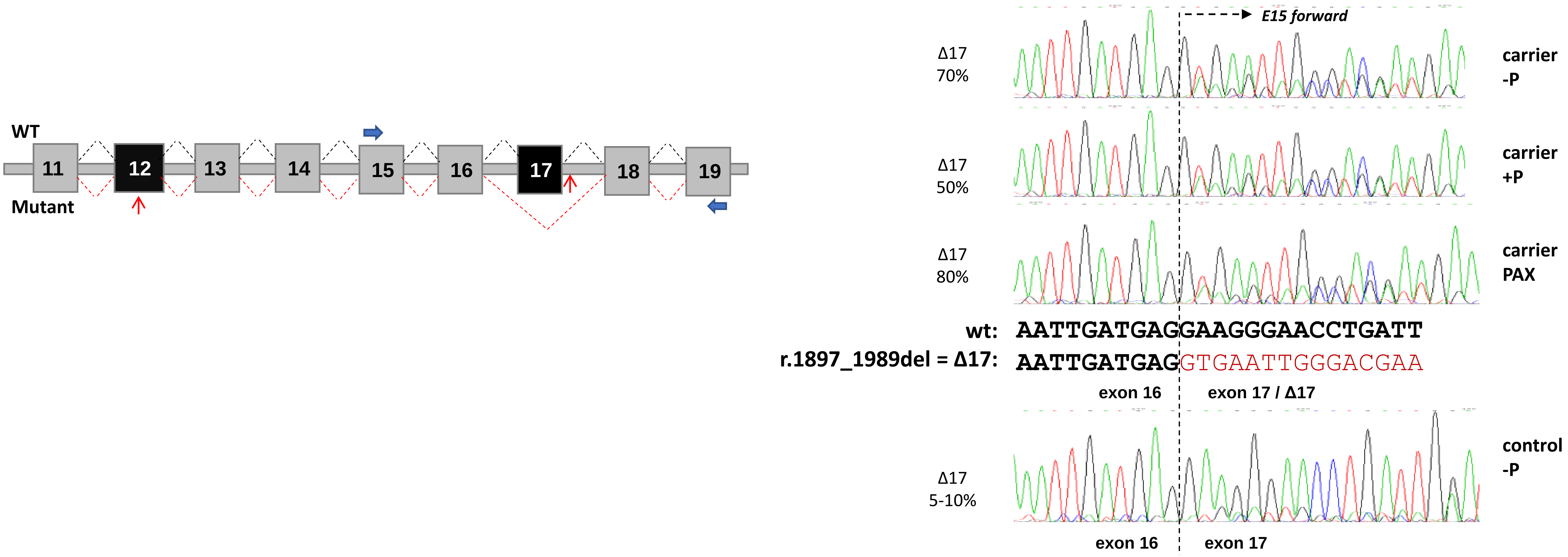

Gene and variant	*MLH1* c.1217G>A co-occurring with c.1989+3dup
Experimental approach	FLT
Laboratory	MUC
Design	E1-E19
Result of the splicing analysis	r.1897_1989del = Δ17; biallelic expression of the variant c.1217G>A

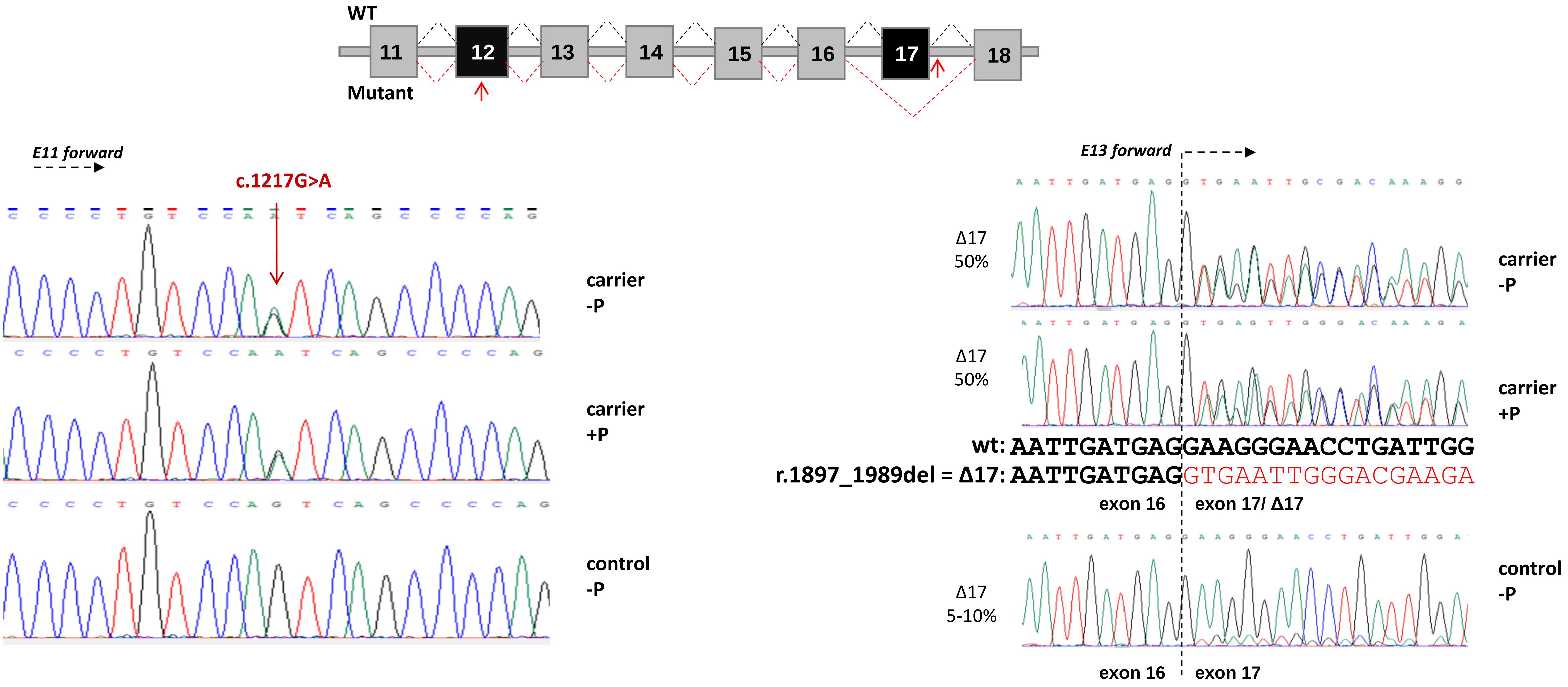

Gene and variant	*MLH1* c.1989+3dup (IVS17)
Experimental approach	Minigene
Laboratory	URO
Result of the splicing analysis	85% Δ17, r.1897_1989del (p.Glu633_Glu663del) and 15% ▼17q(32) r.1989_1990ins[1989+1_1989+31;1989+3dup] p.(Asn665Glnfs*)

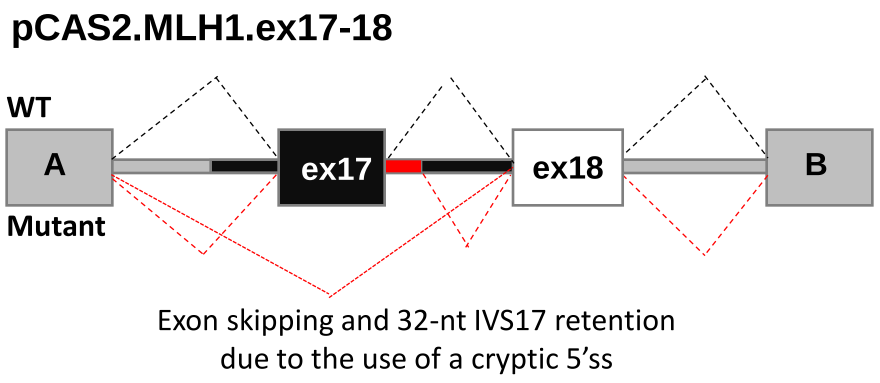

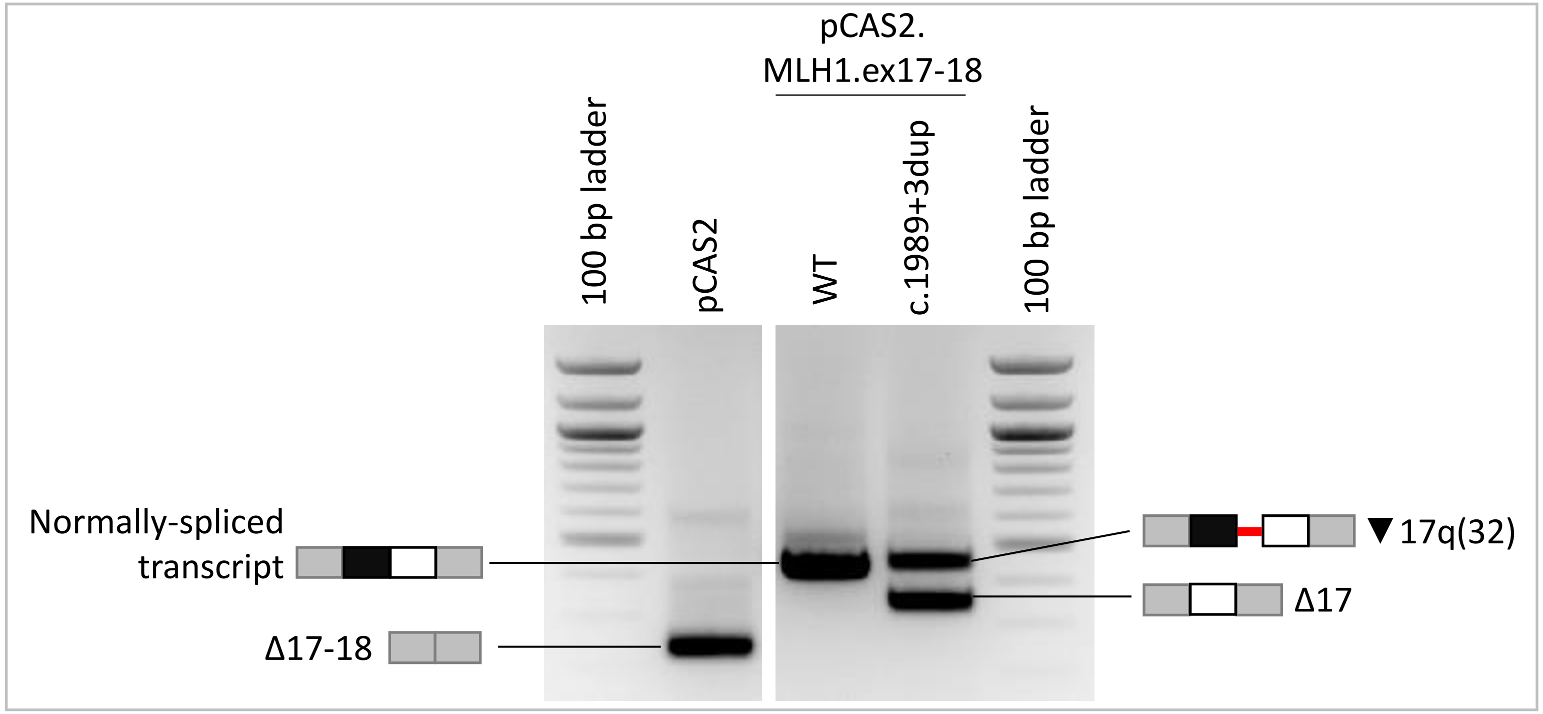

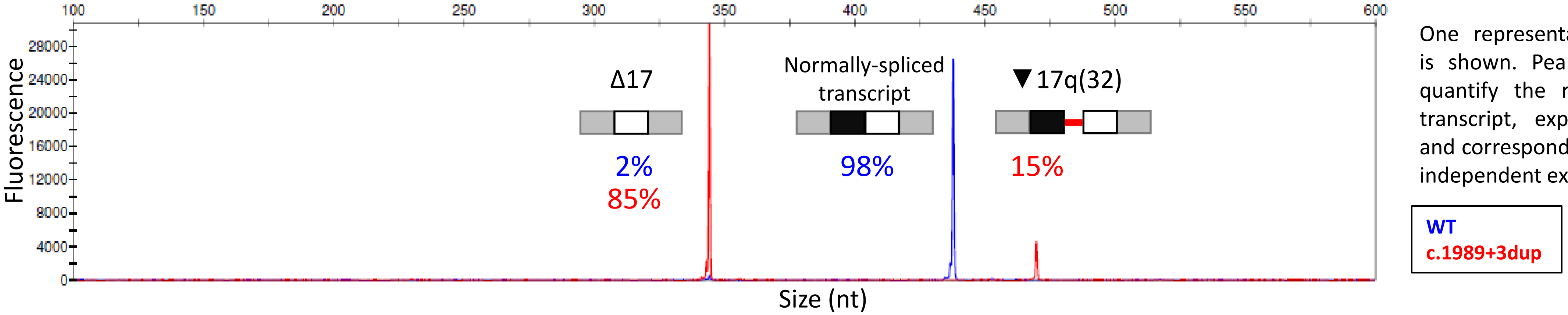

One representative electropherogram is shown. Peak areas were used to quantify the relative levels of each transcript, expressed as percentage, and correspond to the average of three independent experiments
