# Supplementary material for: Splicing analyses for variants in MMR genes: best practice recommendations from the European Mismatch Repair Working Group
Source: Eur J Hum Genet. 2022 Jun 9;30(9):1051–9. doi: 10.1038/s41431-022-01106-w (PMC9437034; doi:10.1038/s41431-022-01106-w)
Supplement: Supplementary file 5 — Supplemental Figure 3_Schematic representation of the SpliceSiteFinder-like and MaxEntScan algorithms splicing predictions [file 41431_2022_1106_MOESM5_ESM.pdf]

MSH2 c.211G>C

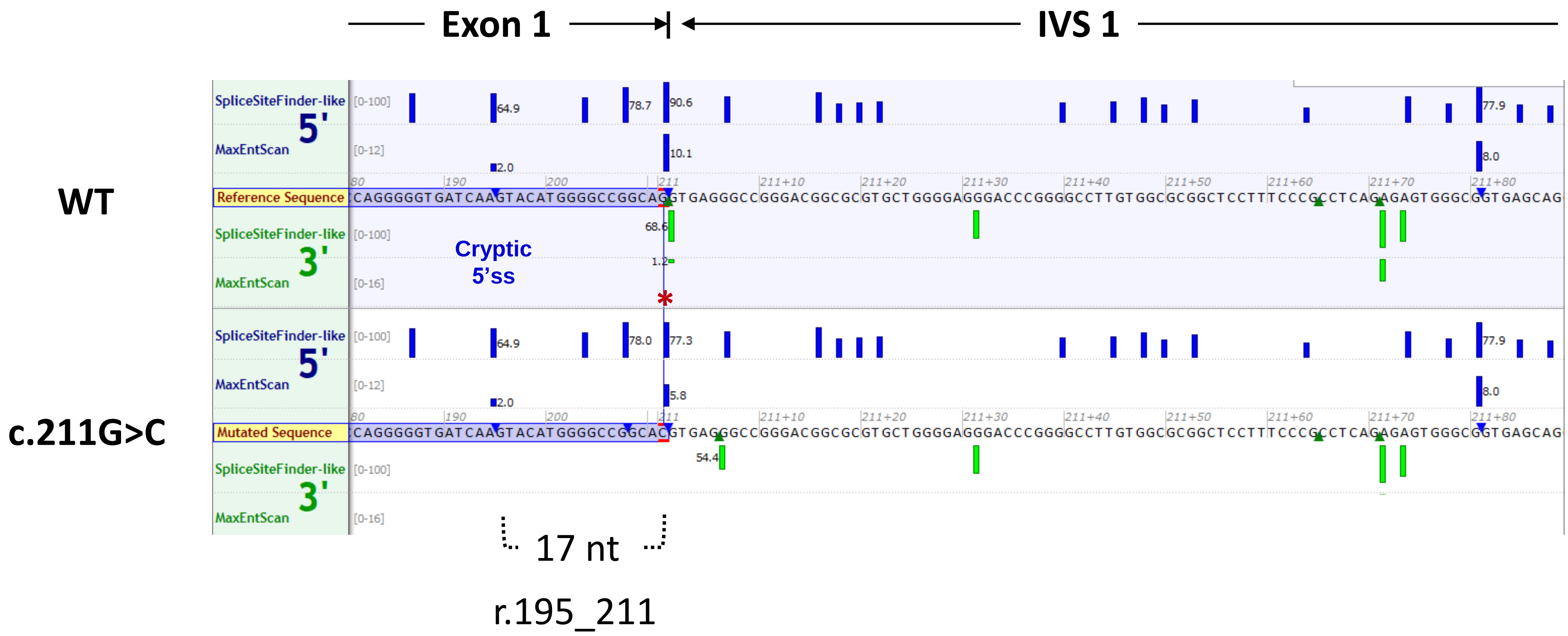

* affected reference splice site

MSH2 c.1276G>A

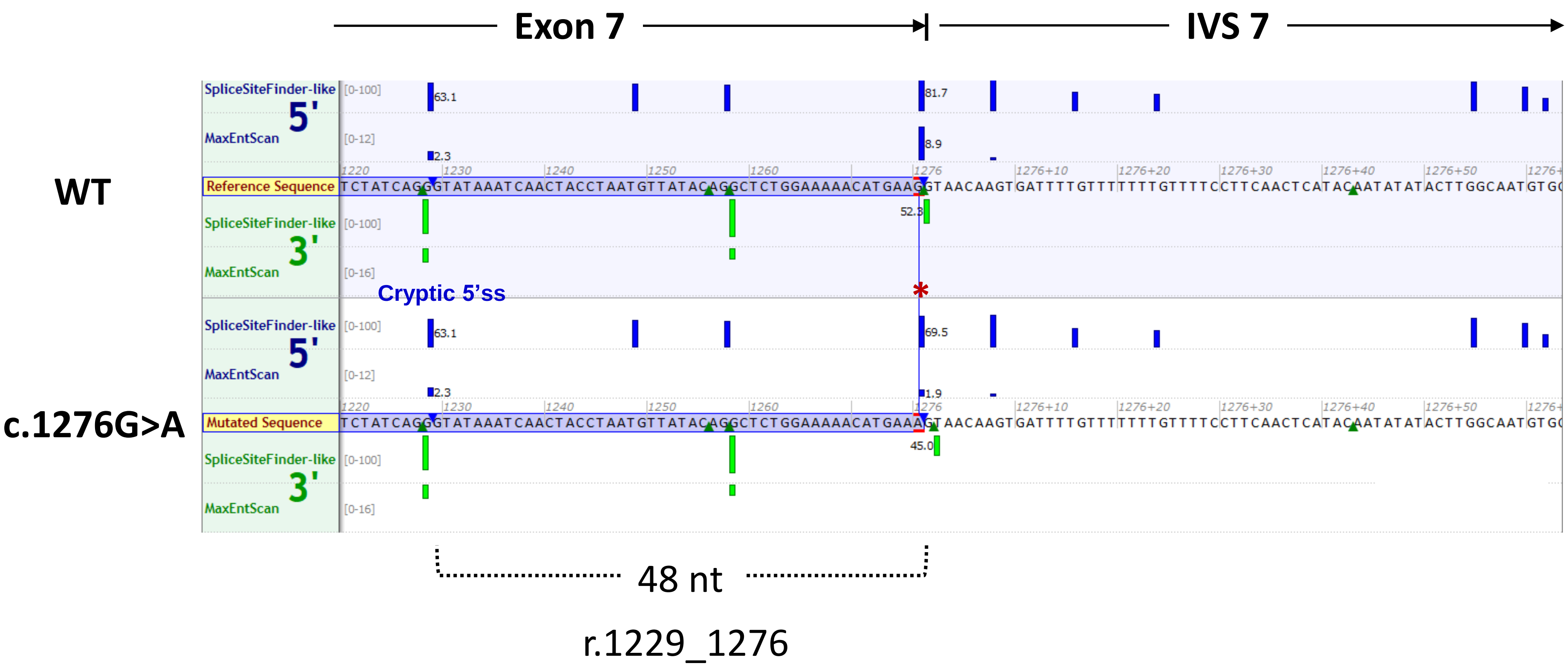

* affected reference splice site

MSH2 c.2459-12A>G

WT

c.2459-12A>G

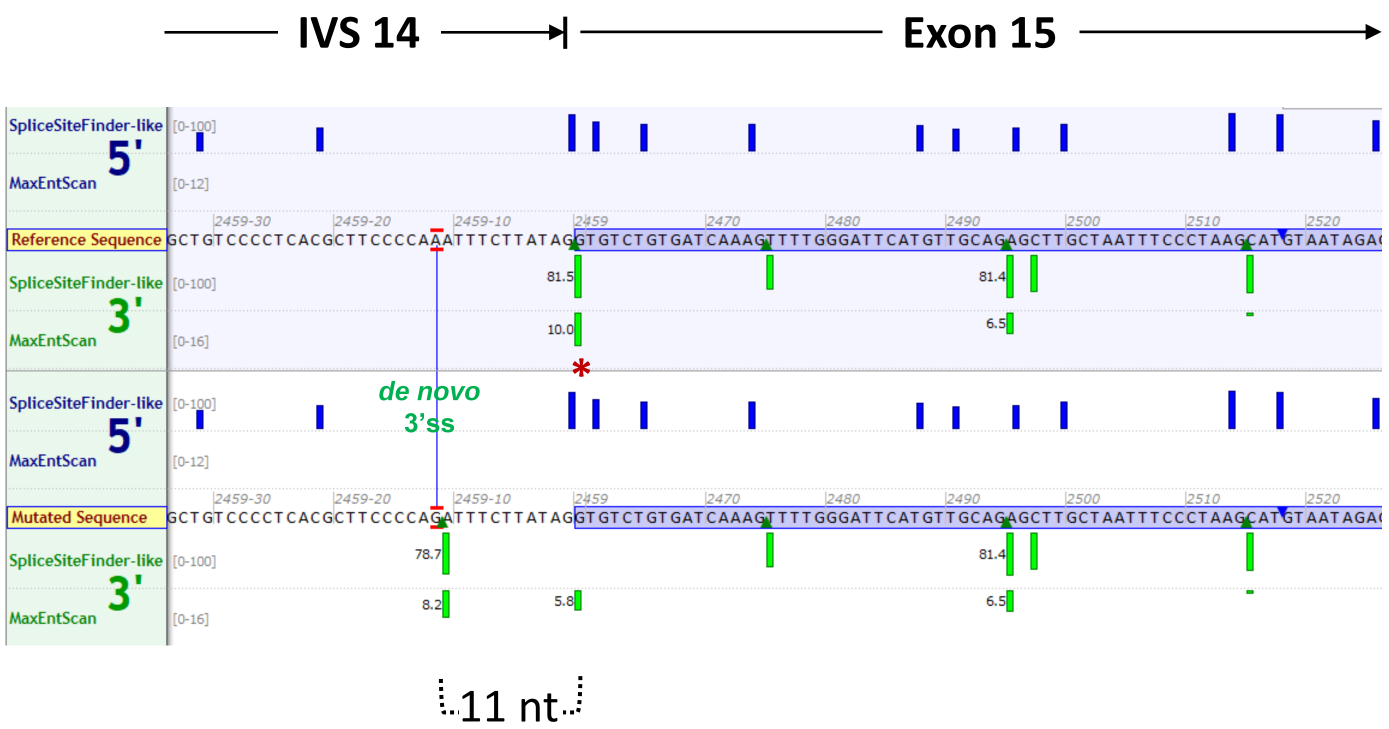

r.2459-11_r.2459-1

* affected reference splice site

MSH6 c.1894A>G

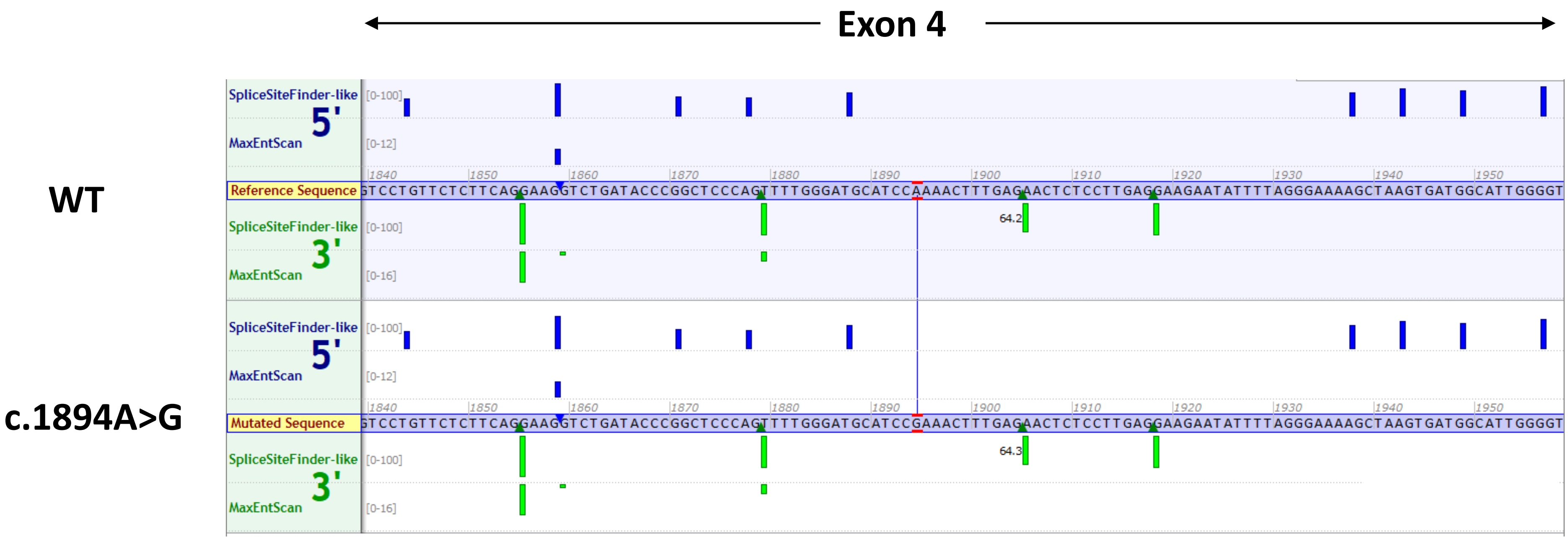

MLH1 c.1039-2A>T

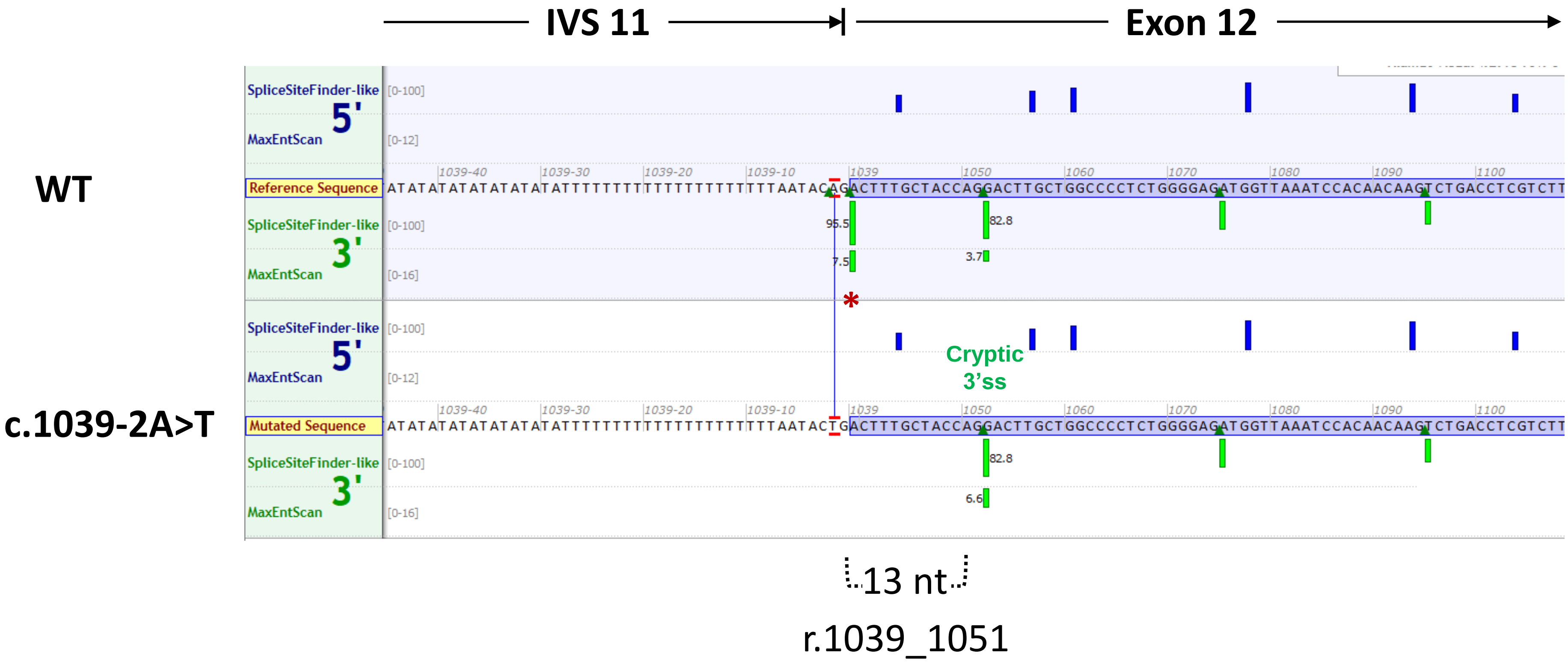

* affected reference splice site

MLH1 c.1217G>A

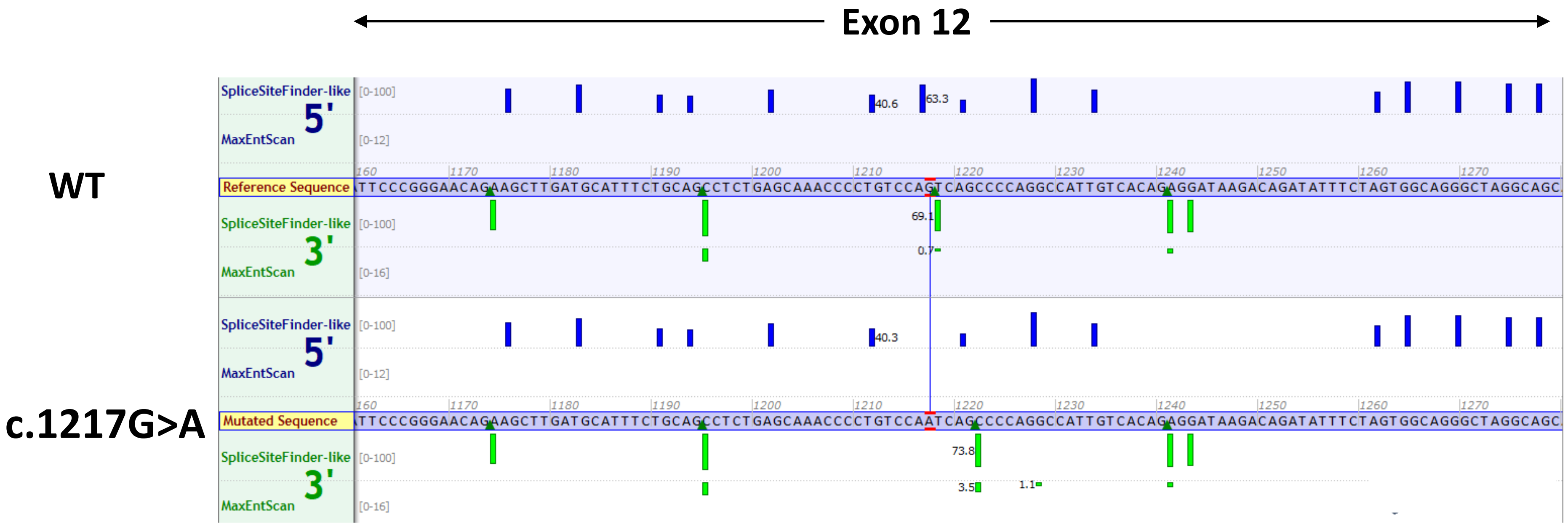

MLH1 c.1989+3dup

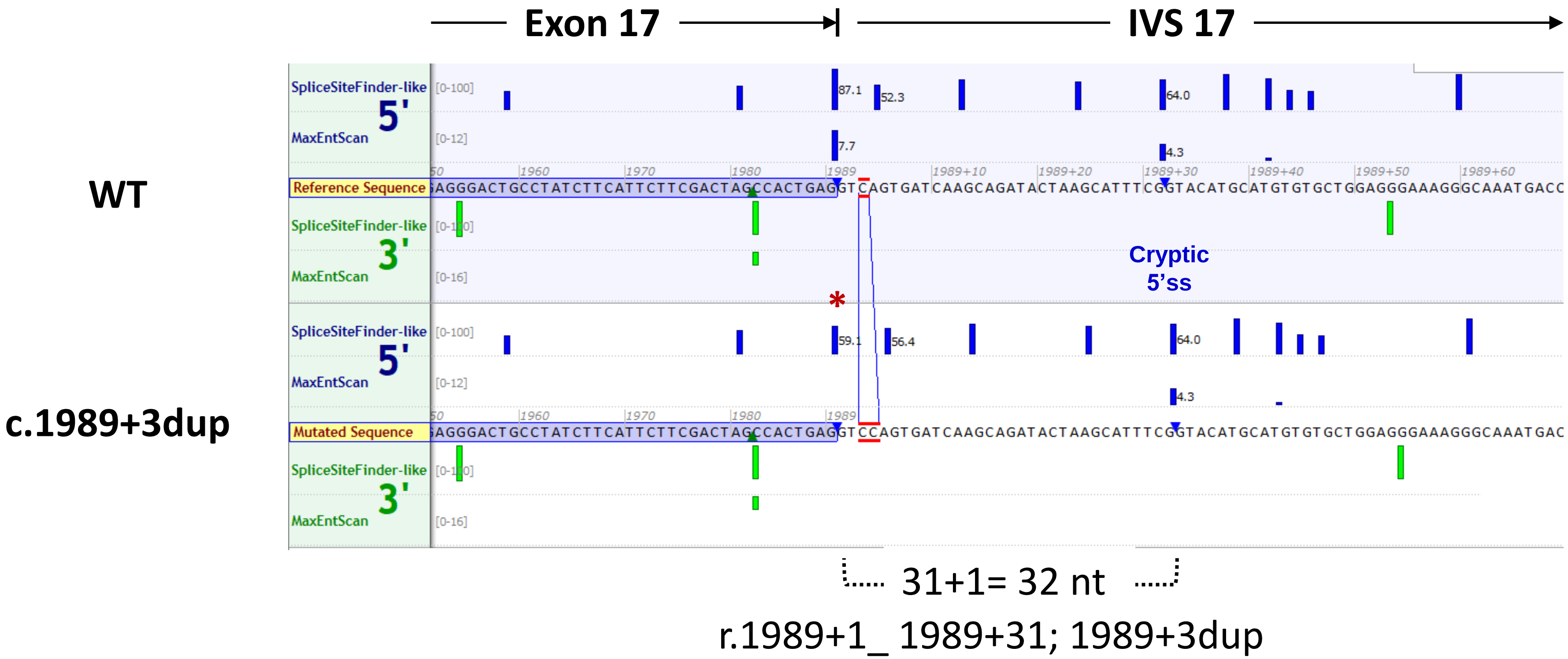

* affected reference splice site
