# Supplementary material for: Splicing analyses for variants in MMR genes: best practice recommendations from the European Mismatch Repair Working Group
Source: Eur J Hum Genet. 2022 Jun 9;30(9):1051–9. doi: 10.1038/s41431-022-01106-w (PMC9437034; doi:10.1038/s41431-022-01106-w)
Supplement: Supplementary file 6 — Supplemental Table 1_Comparative overview of the experimental approaches used in this study [file 41431_2022_1106_MOESM6_ESM.doc]

**Supplemental Table 1:** Comparative overview of the experimental approaches used in this study: targeted transcript section (TTS), full-length transcript (FLT) analyses by RT-PCR of RNA extracted from blood-derived samples. and minigene splicing assays. BCN, Barcelona; MUC, Munich; URO, Rouen.(*) in certain experimental designs the non-use of DNAase treatment might lead to non-specific DNA amplification or diminished efficiency of amplification.

| **Steps of splicing analysis** | **Item** | **Targeted transcript section analysis (BCN)** | **Full-length transcript analysis (MUC)** | **Minigene splicing assays (URO)** |
| --- | --- | --- | --- | --- |
| **1. Sample processing** | **sample tubes** | K3 EDTA blood | PAXgene and heparin blood | Not applicable |
|  | **source of RNA** | short-term culture of PBL (+/- puromycin) | short-term culture of PBL (+/- puromycin) and PAXgene | HeLa cells transfected with pCAS2-MMR minigenes |
| **2. RNA isolation** | **RNA isolation method** | Trizol/phenol-chloroform extraction | RNA blood mini kit (QIAGEN) and  PAXgene Blood RNA extraction kit (PreAnalytiX) | NucleoSpin RNA II kit (Macherey Nagel), except for the study of *MSH2* c.2459-12A>G, where RNA was extracted with the TriPure Isolation Reagent (Roche) |
|  | **DNAse treatment** | No* | yes | yes |
|  | **storage** | -80ºC | -80°C | -80°C |
|  | **minimum quality** | A_260/280_= 1.9-2.0  RIN: 6.9 for PBL, 5.7 for analysable PAXgene RNA | A_260/280_=1.7-2.1  RIN: 6.9 for PBL, 5.7 for analysable PAXgene RNA | A_260/280_= 1.9-2.1 |
|  | **RNA yield** | 5-15 µg | ~ 15 µg | Transfections in 6-well plates: ~ 12-13 µg/well  Transfections in 12-well plates: ~ 4-6 µg/well |
|  | **visualisation on agarose gel** | yes | yes | yes |
| **3. Synthesis of cDNA** | **quantity of total RNA used for cDNA synthesis** | 250 ng or 1 µg | 1 µg | 200 ng RNA for OneStep RT-PCR, except for *MSH6* c.1894A>G for which we used 100ng RNA in OneStep RT-PCR reactions*,* and for *MSH2* c.2459-12A>G, for which we used 1 µg RNA in two-step RT-PCR reactions |
|  | **cDNA synthesis kit*** | Superscript II (Invitrogen) or M-MLV Reverse Transcriptase (Invitrogen) | I-script select (Bio-Rad) | OneStep RT-PCR kit (Qiagen), except for *MSH2* c.2459-12A>G, for which we performed two- step RT-PCR reactions using Superscript II Reverse Transcriptase (Invitrogen) |
|  | **primers** | random hexamers or random hexamers + Oligo d(T) | Oligo d(T) | pCAS-2R primer for OneStep RT-PCR, except for the study of *MSH2* c.2459-12A>G, for which we performed two-step RT-PCR analyses using oligo d(T)_18_ as reverse primer in the RT reactions |
|  | **storage** | -20°C | -20°C | -20°C |
| **4. Gene-specific amplification of cDNA** | **quantity of cDNA as PCR template / total volume** | 1-1.5 µl / 15-20 µl | 1-2 µl / 20 µl | 3 µl cDNA/ 25 µl PCR reaction for the analysis of *MSH2* c.2459-12A>G |
|  | **PCR amplification method** | Megamix Double (Micorzone) or LA Taq DNA (Takara Clontech) | LR PCR with IProof High-Fidelity DNA Polymerase (BioRad) or PrimeSTAR GXL DNA Polymerase (TAKARA Clontech) | Taq Polymerase (ABgene) |
|  | **length of amplified fragment** | Variable depending on the specific design (500 bp - 3000 bp) | 2.452 bp for *MLH1*  2.965 bp for *MSH2*  4.163 bp for *MSH6* | Variable depending on the minigene identities, their WT/variant status and associated splicing outcomes |
|  | **amount of cDNA as template for PCR** | n.a. (1-1.5 µl) | equivalent to 50-100 ng RNA (1-2 µl) | Equivalent to 200 ng RNA from transfected HeLa cells |
|  | **nº of cycles** | 30-35 | 35 | 30 |
|  | **minimum nº of control#** | 2-3 (10 in previous runs) | 2 (10 in previous runs) | Two controls for each variant minigene: (i) RNA from HeLa cells transfected in parallel with the paired WT minigene and (ii) RNA from HeLa cells transfected in parallel with the empty pCAS2 vector |
|  | **visualisation on agarose gel** | yes | yes | yes |
| **5. Sequencing of cDNA fragments** | **PCR purification** | ExoSAP | ExoSAP | NucleoSpin (Macherey Nagel) |
|  | **Sanger sequencing** | BigDye Terminator v.3.1 Sequencing Kit; ABI 3730 DNA sequencer | BigDye Terminator v.3.1 Sequencing Kit; ABI PRISM 3100 or ABI 3730 | BigDye Terminator v.3.1 Sequencing kit; ABI 3130 or ABI3500 automated sequencers (Applied Biosystems) |
|  | **sequence run length** | 600-800 bp | 600-800 bp | The size of the minigene-derived RT-PCR amplicons detected in this study varied between 235 bp and 725 bp. |
