# Supplementary material for: Splicing analyses for variants in MMR genes: best practice recommendations from the European Mismatch Repair Working Group
Source: Eur J Hum Genet. 2022 Jun 9;30(9):1051–9. doi: 10.1038/s41431-022-01106-w (PMC9437034; doi:10.1038/s41431-022-01106-w)
Supplement: Supplementary file 7 — Supplemental Table 2_Specific primer sequences used in this study. [file 41431_2022_1106_MOESM7_ESM.docx]

**Supplemental Table 2**:

1. Specific primer sequences for RT-PCR TTS amplification and sequencing in BCN.

| **RT-PCR Primer name** | **Primer sequence 5`-3`** | **Size of the expected amplicon (bp)** | **Annealing temperature** |
| --- | --- | --- | --- |
| *MSH2* E1 forward | AGAAGCCGACCACCACAGT | 594 | 60 |
| *MSH2* E4 reverse | TCTGTGATCAGAATTCCTCCTCTTTGA |  |  |
| *MSH2* E6 forward | TCAGTCTCTGGCTGCCTTG | 1173 | 64 |
| *MSH2* E13 reverse | AGCCCCTACTCGGGCTAAG |  |  |
| *MSH2* E14 forward | GGGAAGAGGAACTTCTACCTACG | 878 | 59 |
| *MSH2* E16 reverse | TGCCTATGTCAATTGCAAACAG |  |  |
| *MLH1* E11 forward | GCACCCCACAAAGCATGAAG | 810 | 60 |
| *MLH1* E15 reverse | CGATAACCTGAGAACACCAAAAT |  |  |
| *MLH1* E13 forward | CCCGAAAGGAAATGACTG | 274 | 55 |
| *MLH1* E15 reverse | CGATAACCTGAGAACACCAAAAT |  |  |
| *MLH1* E15 forward | GCCAATTTTGGTGTTCTCAG | 600 | 55 |
| *MLH1* E19 reverse | AGAACACATCCCACAGTGC |  |  |
| *MSH6* E3 forward | ACTGAGAGCAATGCAACGTG | 2866 | 64 |
| *MSH6* E5 reverse | TAGGCTTTGCCATTTTCCTG |  |  |

1. Specific primer sequences for RT-PCR FLT and TTS amplification in MUC.

| **RT-PCR Primer name** | **Primer sequence 5`-3`** | **Size of the expected amplicon (bp)** | **Annealing temperature** |
| --- | --- | --- | --- |
| *MLH1* FLT forward | GGATGGCGTAAGCTACAGCT | 2452 | 61 |
| *MLH1* FLT reverse | AGAACACATCCCACAGTGC |  |  |
| *MSH2* FLT forward | GTCGCGCATTTTCTTCAACC | 2965 | 59 |
| *MSH2* FLT reverse | AAGTTGATAGCCCATGGGC |  |  |
| *MSH6* FLT forward | CGTCCGACAGAACGGTTGG | 4163 | 60 |
| *MSH6* FLT reverse | CCACCTTTGTCAGAAGTCAAC |  |  |
| *MLH1* E10 forward | TCAACTTCCTTGAGAAAAGCC | 816 | 58 |
| *MLH1* E14 reverse | CAAGGCCCACTGAGGATTC |  |  |
| *MLH1* E11 forward | GCAGCACATCGAGAGCAAG | 969 | 58 |
| *MLH1* E17 reverse | AAAGGGGGCACATAGTTGTC |  |  |
| *MSH2* E1 forward | CCAGGGGGTGATCAAGTAC | 656 | 58 |
| *MSH2* E3 reverse | TGTCTCTGGCCATCAACTG |  |  |
| *MSH2* E5 forward | CATCACTGTCTGCGGTAAT | 452 | 58 |
| *MSH2* E8 reverse | TTTCCTGAAACTTGGAGAAGTC |  |  |
| *MSH2* E13 forward | GTTCATGGCTGAAATGTTGG | 419 | 58 |
| *MSH2* E15 reverse | TCATATCCTTGCGATTCTCCA |  |  |
| *MSH6* E3 forward | ACTGAGAGCAATGCAACGT | 2737 | 60 |
| *MSH6* E5 reverse | ATCTTCCGGCAACAGAATTAC |  |  |
| *MSH6* E1 forward | CGCTGAGTGATGCCAACAA | 765 | 60 |
| *MSH6* E4 reverse | CGGCTACTTCGCCTAGATCC |  |  |
| *MSH6* E3 forward | ACTGAGAGCAATGCAACGT | 3613 | 60 |
| *MSH6* E10 reverse | CCACCTTTGTCAGAAGTCAAC |  |  |

1. Specific primer sequences for Sanger sequencing in MUC.

| **RT-PCR Primer name** | **Primer sequence 5`-3`** |
| --- | --- |
| *MLH1* E1 forward sequencing | GCGGCCAGCTAATGCTATC |
| *MLH1* E3 reverse sequencing | CACCTCGAAAGCCATAGGTA |
| *MLH1* E6 forward sequencing | TAGCCACGAGGAGAAAAGC |
| *MLH1* E10 forward sequencing | TCAACTTCCTTGAGAAAAGCC |
| *MLH1* E12 forward sequencing | GATAAGACAGATATTTCTAGTG |
| *MLH1* E13 forward sequencing | GGAGAAGGATCATTAACCTC |
| *MLH1* E16 forward sequencing | TGGTCCCAAAGAAGGACTTG |
| *MLH1* E19 reverse sequencing | GATCAGGCAGGTTAGCAAGC |
| *MSH2* E2 forward sequencing | TGGTTCGTCAGTATAGAGTTG |
| *MSH2* E3 forward sequencing | AGGACTGTGTGAATTCCCTG |
| *MSH2* E7 forward sequencing | CAAACTTACAAGATTGTTACCG |
| *MSH2* E10 forward sequencing | TAGTACTGTAGATATCCAGAAG |
| *MSH2* E11 forward sequencing | CCAGGATGCCATTGTTAAAG |
| *MSH2* E14 forward sequencing | GGCCAATCAGATACCAACTG |
| *MSH6* E2 reverse sequencing | CTGTACATGAACACGGACTG |
| *MSH6* E2 forward sequencing | GAGATTTGGTTTGGGCCAAG |
| *MSH6* E4-2 reverse sequencing | CATTCTCTTCCGCTTTCGAG |
| *MSH6* E4-3 reverse sequencing | TAGATGCATCAAAATCGGGG |
| *MSH6* E4-4 reverse sequencing | TCTACATCGTGCCTCCATCA |
| *MSH6* E4-5 reverse sequencing | TAAATCTCGAACAATGGCGA |
| *MSH6* E4-6 reverse sequencing | AGCACCTGGGGTAACATCAC |
| *MSH6* E4-7 reverse sequencing | GCACCATTCGTTGATAGGCT |
| *MSH6* E4-8 reverse sequencing | TGGTTCTGACTCTTCAGGGG |
| *MSH6* E4-9 reverse sequencing | TTTCGAGCCTTTTCATGGTC |
| *MSH6* E4-10 reverse sequencing | TCGTTTACAGCCCTTCTTGG |
| *MSH6* E5 reverse sequencing | ATCTTCCGGCAACAGAATTAC |
| *MSH6* E6 reverse sequencing | TCTGTCTGAGGCACCAAGTC |
| *MSH6* E8 forward sequencing | CTTGCTGAGACTATAAAATGTCG |
| *MSH6* E9 reverse sequencing | GGCTGGGGTCTTCACATTC |
