# Supplementary material for: Splicing analyses for variants in MMR genes: best practice recommendations from the European Mismatch Repair Working Group
Source: Eur J Hum Genet. 2022 Jun 9;30(9):1051–9. doi: 10.1038/s41431-022-01106-w (PMC9437034; doi:10.1038/s41431-022-01106-w)
Supplement: Supplementary file 8 — Supplemental Table 3_Cloning and RT-PCR primers for minigene splicing assays [file 41431_2022_1106_MOESM8_ESM.doc]

**Supplemental Table 3**: Cloning and RT-PCR primers for minigene splicing assays

1. **Cloning primers for minigene splicing assays**

| **Gene** | **Amplicon** | **Primer Name** | **Primer sequence (5’-3’)^1^** | **Annealing temperature** | **Size of the amplicon (bp)** |
| --- | --- | --- | --- | --- | --- |
| *MLH1* | Exon 12 | MLH1-Ex12-BamHI_F | GACCGGATCCTTACTAGCTTCTTTCTTAGTACTGCT | Touchdown PCR.  Annealing temperature gradually reduced from 60°C to 55°C (0.5°C/cycle) for 10 cycles, then 55°C for 25 cycles | 632 |
|  |  | MLH1-Ex12-Mlu1_R | GACCACGCGTCAGAGGGCAAGTCAGGCA |  |  |
| *MLH1* | Exon 17-18 | MLH1-Ex17-18-BamHI_F | GACCGGATCCTCAGCCCAATCAAGTAACGTG | Touchdown PCR.  Annealing temperature gradually reduced from 60°C to 55°C (0.5°C/cycle) for 10 cycles, then 55°C for 25 cycles | 793 |
|  |  | MLH1-Ex17-18-Mlu1_R | GACCACGCGTAAAGCTGACTGTTGTGTTTGAA |  |  |
| *MSH2* | Exon 7 | MSH2-Ex7-BamHI_F | GACCGGATCCTACTGCGCCCAGCAGAT | Touchdown PCR.  Annealing temperature gradually reduced from 60°C to 55°C  (0.5°C/cycle) for 10 cycles, then 55°C for 25 cycles | 508 |
|  |  | MSH2 Ex7 MluI_R | GACCACGCGTTGGCAACGGAGCAAGAC |  |  |
| *MSH2* | Exon 12 | MSH2-Ex12-BamHI_F | GACCGGATCCTCTATGTTGAGTTTTAGGTGGG | Touchdown PCR.  Annealing temperature gradually reduced from 60°C to 55°C (0.5°C/cycle) for 10 cycles, then 55°C for 25 cycles | 581 |
|  |  | MSH2-Ex12-MluI_R | GACCACGCGTTTTCTAATGTTAAGAACTGGGA |  |  |
| *MSH2* | Exon 15 | MSH2-15-BamHI-F | ACCGGATCCTGCTAATGTATTTGAAGTAATCCGA | 57°C | 479 |
|  |  | MSH2-15-MluI-R | GACCACGCGTCACAGAGATAGATTCTTTGCCAT |  |  |
| *MSH6* | Exon 4 | MSH6.Ex4 BamHI-F | GACCGGATCCTTAGCCTAAAGTTGGGCAAG | 56°C | 2978 |
|  |  | MSH6-Ex4.MluI-R | GACCACGCGTAAGGCCAAAGGGCTACTAAG |  |  |

^1^ The underlined sequences correspond to restriction sites for BamHI or MluI.

1. **RT-PCR primers for minigene splicing assays**

| **Primer Name** | **Primer sequence (5’-3’)** | **Annealing temperature** | **Size of the amplicon(s) (bp)** |
| --- | --- | --- | --- |
| pCAS-KO1-F | TGACGTCGCCGCCCATCAC | 57°C | Variable depending on the identity of the minigene constructs, their WT/variant status and associated splicing outcomes.  The size of the minigene-derived RT-PCR amplicons detected in this study varied between 235 bp and 725 bp. |
| pCAS-2R | ATTGGTTGTTGAGTTGGTTGTC |
