# Supplementary material for: Splicing analyses for variants in MMR genes: best practice recommendations from the European Mismatch Repair Working Group
Source: Eur J Hum Genet. 2022 Jun 9;30(9):1051–9. doi: 10.1038/s41431-022-01106-w (PMC9437034; doi:10.1038/s41431-022-01106-w)
Supplement: Supplementary file 9 — Supplemental Table 4_Overview and comparative analysis of the RT-PCR splicing results [file 41431_2022_1106_MOESM9_ESM.docx]

## Supplemental Table 4: Overview and comparative analysis of the RT-PCR splicing results

Results of the experimental splicing analyses of the samples from patients harboring the MMR variants under investigation in comparison to controls with wild-type MMR sequences. Differences between the experimental approaches are shown, with major differences being highlighted in **bold (**affecting the interpretation of the results leading to a divergent variant classification based on the splicing result) as compared to minor differences (not affecting interpretation). (*) See the collected evidences for pathogenicity assessment of MMR variants in **Table 3.** (^) Physiological alternative transcripts previously detected in controls according to Ref. 41. +P: cDNA from lymphocyte culture incubated with puromycin, -P: cDNA from lymphocyte culture not incubated with puromycin; BCN: Barcelona, CRC: colorectal cancer, del: deletion or exon skipping, CMMR-D: constitutional mismatch repair-deficiency, E: exon, FLT: full-length transcript analysis, IVS: intervening sequence/intron, MUC: Munich, n.a.: not analysable, n.d.: not determined, NMD: nonsense-mediated mRNA decay, ns, normally-spliced, nt: nucleotide(s), PAX: cDNA from RNA extracted from blood collected in PAXgene tubes, TTS: targeted transcript section analysis, URO: Rouen, VUS: variant of uncertain significance, WT: wild-type, y: years; “informative variant”: heterozygous exonic variant useful for allele-specific expression (ASE) analyses.

| **Gene and VUS (samples)** | **Observation** | **TTS MSH2**  **E1-4 (BCN)** | **FLT MSH2**  **E1-16 (MUC)** | **TTS MSH2**  **E1-3 (MUC)** | **pCAS2.MSH2.ex1 minigene**  **EA-B (URO)** | **Differences** |
| --- | --- | --- | --- | --- | --- | --- |
| ***MSH2***  **Exon 1**  **c.211G>C**  **p.(Gly71Arg) (-P/+P)** | Detection of variant-induced aberrant splicing | Yes | Yes | n.a.  preferential amplification of aberrant transcript | n.d. given that the pCAS2 minigene vector is not suited to test the impact of variants mapping to/near terminal exons | **minigene n.a.,**  **TTS E1-3 n.a.** |
|  | Aberrant transcript(s) and predicted protein alteration(s) | r.195_211del  (p.Tyr66Serfs*10) = Δ1q(17) | r.195_211del  (p.Tyr66Serfs*10) = Δ1q(17) and r.-16_211del p.? = Δ1q(227) | r.-16_211del p.? = Δ1q(227) | n.a. | additional isoform detected in FLT due to primer location |
|  | % of aberrant transcript observed in agarose gel in variant-carrying sample | -P/+P: 0% | -P/+P: 0% | -P/+P: 100% | n.a. |  |
|  | % of aberrant transcript observed in agarose gel in controls | 0% | 0% | 20% | n.a. |  |
|  | % of aberrant transcript(s) in Sanger sequence in variant-carrying sample | -P: 10% Δ1q(17)  +P: 20% Δ1q(17) | -P: 5% Δ1q(17)  +P: Σ30%: 25% Δ1q(17), 5% Δ1q(227) | -P/+P: 100% Δ1 | n.a. | % of aberrant transcripts differs slightly (±5) between TTS E1-4 and FLT, **overestimated in TTS E1-3** |
|  | % of aberrant transcript(s) in Sanger sequence in controls | 0% | 0% | 20-25% Δ1 | n.a. | **aberrant splicing in controls in minigene and**  **TTS E1-3** |
|  | Allelic expression of WT/VUS in Sanger sequence | r.211G wt 100%, VUS 0% | r.211G wt 100%, VUS 0% | n.a.: 100% Δ1 | n.a. |  |
|  | Level of normally-spliced transcript produced by variant allele in % | 0% | 0% | 0% | n.a. |  |
|  | Splicing effect and preliminary classification based on splicing evidences | complete splicing defect, expression analysis ruled out generation of ns transcripts from the variant allele (Class 5) | complete splicing defect, expression analysis ruled out generation of ns transcripts from the variant allele (Class 5) | n.a. | n.a. | - |
|  | **Final classification*** | **Class 5 (combining TTS with FLT results, or each with bioinformatics prediction)**  The complete splicing defect activates a cryptic 5` splice sites in exon 1. Absence of ns transcripts from the variant allele was shown. | | | | |
| **Gene and VUS (samples)** | **Method** | **TTS MSH2**  **E6-13 (BCN)** | **FLT MSH2**  **E1-16 (MUC)** | **TTS MSH2**  **E5-8 (MUC)** | **pCAS2.MSH2.ex7 minigene EA-B (URO)** | **Differences** |
| ***MSH2***  **Exon 7**  **c.1276G>A**  **p.(Gly426Arg)**  **(-P/+P)** | Detection of variant-induced aberrant splicing | Yes | n.d. in first cDNA,  Yes in second cDNA | Yes | Yes | first cDNA in FLT n.a. |
|  | Aberrant transcript(s) and predicted protein alteration(s) | r.1229_1276del (p.Ile411_Gly426del16) = Δ7q(48) | r.1229_1276del (p.Ile411_Gly426del16) = Δ7q(48) | r.1229_1276del(p.Ile411_Gly426del16) = Δ7q(48) | r.1229_1276del (p.Ile411_Gly426del16) = Δ7q(48) |  |
|  | % of aberrant transcript observed in agarose gel in variant-carrying sample | -P: 30-50%  +P: 50% | -P/+P: 0% | -P: 30%  +P: 50% | approximately >99% | agarose gel not informative for FLT |
|  | % of aberrant transcript observed in agarose gel in controls | 0% | 0% | 0% | 0% |  |
|  | % of aberrant transcript(s) in Sanger sequence in variant-carrying sample | -P: 30-50% Δ7q(48)  +P: 50% Δ7q(48) | -P: 10-20% Δ7q(48)  +P: 20-30% Δ7q(48) | -P: 30% Δ7q(48)  +P: 60% Δ7q(48) | approximately >99% Δ7q(48) | % of aberrant transcripts lower in FLT (25%) than TTS analysis |
|  | % of aberrant transcript(s) in Sanger sequence in controls | 0% | 0% | 0% | 0% |  |
|  | Allelic expression of WT/VUS in Sanger sequence | r.1276G WT100%, VUS 0% | r.1276G WT 100%, VUS 0% | r.1276G WT100%, VUS 0% | n.a. |  |
|  | Level of normally-spliced transcript produced by variant allele in % | 0% | 0% | 0% | 1% | Very residual ns transcript detected in minigene |
|  | Splicing effect and preliminary classification based on splicing evidences | complete splicing defect, expression analysis ruled out generation of ns transcripts from the variant allele (Class 5) | complete splicing defect, expression analysis ruled out generation of ns transcripts from the variant allele, aberrant splicing intensity within splicing defect threshold (Class 5) | complete splicing defect, expression analysis ruled out generation of ns transcripts from the variant allele (Class 5) | complete splicing defect, analysis ruled out generation of ns transcripts from the variant allele (Class 5) | - |
|  | **Final classification*** | **Class 5 (by each splicing assay result alone, combined with variant literature and bioinformatics prediction)**  By activating a cryptic splice site in exon 7, the complete splicing defect resulted in an in-frame deletion Δ7q(48), which is assumed to affect a functional protein domain (lever). Absence of ns transcripts from the variant allele was shown. | | | | |
| **Gene and VUS (samples)** | **Method** | **TTS MSH2 E14-16 (BCN)** | **FLT MSH2 E1-16 (MUC)** | **TTS MSH2 E13-15 (MUC)** | **pCAS2.MSH2.ex15 minigene**  **EA-B (URO)** | **Differences** |
| ***MSH2***  **Intron 14**  **c.2459-12A>G**  **p.?**  **(-P/+P, PAX1, PAX2)** | Detection of variant-induced aberrant splicing | Yes | Yes | Yes | Yes |  |
|  | Aberrant transcript and predicted protein alteration(s) | r.2458_2459insATTTCTTATAG  (p.Gly820Aspfs*4) = ▼15p(11) | r.2458_2459insATTTCTTATAG  (p.Gly820Aspfs*4) = ▼15p(11) | r.2458_2459insATTTCTTATAG  (p.Gly820Aspfs*4) = ▼15p(11) | r.2458_2459insATTTCTTATAG  (p.Gly820Aspfs*4) = ▼15p(11) |  |
|  | % of aberrant transcript in agarose gel in variant-carrying sample | -P/+P/PAX: 0%, n.a. | -P/+P/PAX: 0%, n.a. | -P/+P/PAX: 0%, n.a. | >95% ▼15p(11) |  |
|  | % of aberrant transcript in agarose gel in controls | 0% | 0% | 0% | 0% |  |
|  | % of aberrant transcript in Sanger sequence in variant-carrying sample | -P: 0-5% ▼15p(11)  +P: ~20% ▼15p(11)  PAX1: 0-5% ▼15p(11)  PAX2: 0-5% ▼15p(11) | -P: 0-5% ▼15p(11)  +P: 15-35% ▼15p(11)  PAX1: n.a.  PAX2: 0% ▼15p(11) | -P: 0-5% ▼15p(11)  +P: 15-35% ▼15p(11)  PAX1: n.a.  PAX2: 0-5% ▼15p(11) | >95%% ▼15p(11) |  |
|  | % of aberrant transcript in Sanger sequence in controls | 0% | 0% | 0% | 0% |  |
|  | Allelic expression of WT/VUS in Sanger sequence | n.a., intronic site | n.a., intronic site,  no informative variant | n.a., intronic site | n.a. |  |
|  | Level of normally-spliced transcript produced by variant allele in % | n.a., due to absence of informative variants | n.a., aberrant splicing is elevated 15-35% above threshold, but strength of splicing defect not determined in, absence of informative variants | n.a. | 0% |  |
|  | Splicing effect and preliminary classification based on splicing evidences | variant allele results in a splicing defect of unknown strength,analysis cannot rule out generation of ns transcripts from the variant allele (Class 3) | variant generates a splicing defect of unknown strength, intensity of aberrant transcripts within splicing defect threshold,  analysis cannot rule out generation of ns transcripts from the variant allele (Class 3) | no additional information gained, variant allele results in a splicing defect of unknown strength, analysis cannot rule out generation of ns transcripts from the variant allele (Class 3) | variant induces a complete splicing defect, but needs supporting evidence (Class 3) | absence of ns transcript generation from the variant allele could only be shown by minigene assay, but needs verification by cDNA analysis for classification according to InSiGHT criteria |
|  | **Final classification*** | **Class 5 (combining minigene with PT/FLT results)**  The complete splicing defect created a new 3’splice site inserting the last 11 nucleotides of intron 14 ▼15p(11), disrupting the reading frame, which leads to a premature stop codon and NMD. Absence of ns transcripts from the variant allele was shown. | | | | |
| **Gene and VUS (samples)** | **Method** | **TTS MSH6**  **E3-5 (BCN)** | **FLT MSH6 E1-10**  **or long TTS E3-10 (MUC)** | **TTS MSH6**  **E3-5 (MUC)** | **pCAS2-MSH6-ex4 minigene (URO)** | **Differences** |
| ***MSH6***  **Exon 4**  **c.1894A>G**  **p.(Lys632Glu)**  **(-P/+P)** | Detection of variant-induced aberrant splicing | n.a., due to high level of alternative splicing of E4 in controls | n.d. in FLT, not amplifiable;  no aberrant splicing of E4 in long TTS E3-10 | n.a., due to high level of alternative splicing of E4 in controls | n.d. due to incorrect splicing of wt construct | **TTS n.a.,**  **FLT n.a.,**  **minigene n.a.,** but in long TTS |
|  | Aberrant transcript(s) and predicted protein alteration(s) | - | No aberrant splicing, only alternative splicing of E4, level comparable to controls | - | n.a. |  |
|  | % of aberrant transcript observed in agarose gel in variant-carrying sample | -P/+P: 5-10% r.628_3172 (p.Val210Metfs*21) = Δ4 | -P: 5% Δ4  +P: 10% Δ4 | -P: 10% Δ4  +P: 30% Δ4 | n.a. |  |
|  | % of aberrant transcript observed in agarose gel in controls | ~0-20% Δ4 | ~10-30% Δ4 | ~10-40% Δ4 | n.a. |  |
|  | % of aberrant transcript(s) in Sanger sequence in variant-carrying sample | -P: 10-20% Δ4  +P: 40-50% Δ4 | -P: 10% Δ4  +P: 20-25% Δ4,  but not allele-specific due to biallelic expression of WT/VUS | -P: ~15% Δ4  +P: 30-60% del E4,  but no allele-specificity assumed due to biallelic expression of WT/VUS | n.a. | % of aberrant transcripts high in short TTS +P |
|  | % of aberrant transcript(s) in Sanger sequence in controls | -P: 0-20% Δ4+P: 20-50% Δ4 | -P: 5-15% Δ4  +P: 10-35% Δ4 | -P: 10-20% Δ4  +P: 20-50% Δ4 | 100% r.628_2682del (p.Val210_Gln894del) = Δ4p(2055) due to usage of a cryptic 3’ splice site at c.2683 | % of alternative splicing higher in short TTS +P, **aberrant splicing in minigene** |
|  | Allelic expression of WT/VUS in Sanger sequence | -P/+P: r.1894A/G 50% | n.a. in FLT  -P/+P in TTS E3-10: r.1894A/G 50% | -P/+P: r.1894A/G 50% | n.a. |  |
|  | Level of normally-spliced transcript produced by variant allele in % | probably 100%, biallelic expression of WT/VUS | 100%, biallelic expression of WT/VUS in TTS E3-10 | probably 100%, biallelic expression of WT/VUS | n.a. |  |
|  | Splicing effect and preliminary classification based on splicing evidences | n.d. | Splice-neutral missense variant (Class 3) | n.d. | n.d. | effect of splicing determinable in TTS analysis by excluding allele-specific splicing of exon 4 by the balanced variant expression combined with FLT threshold definition for alternative splicing |
|  | **Final classification*** | **Class 3 (splice-neutral missense variant with unknown significance on protein level)** | | | | |
| **Gene and VUS (samples)** | **Method** | **TTS MLH1 E11-15 (BCN)** | **FLT MLH1 E1-19 (MUC)** | **TTS MLH1 E10-14 (MUC)** | **pCAS2.MLH1.ex12 minigene**  **EA-B (URO)** | **Differences** |
| ***MLH1***  **Intron 11**  **c.1039-2A>T**  **p.?**  **(-P/+P, PAX1, PAX2)** | Detection of variant-induced aberrant splicing | Yes | Yes | Yes | Yes |  |
|  | Aberrant transcript(s) and predicted protein alteration(s) | r.1039_1409del  (p.Thr347Lysfs*8) = Δ12 | r.1039_1409del  (p.Thr347Lysfs*8) = Δ12,  r.678_1409del (p.Glu227_Arg470del) = Δ9-12,  r.885_1409del (p.Ser295_Pro469del) = Δ11-12r.1039_1051del p.(Thr347Aspfs*16) = Δ12p(13)  **,** -P: 0%; +P: <5% | r.1039_1409del  (p.Thr347Lysfs*8) = Δ12 | r.1039_1051del (p.Thr347Aspfs*16) = Δ12p(13)  r.1039_1409del  (p.Thr347Lysfs*8) = Δ12 | additional aberrant isoforms detectable in FLT and minigene assay |
|  | % of aberrant transcript(s) in agarose gel in variant-carrying sample | -P: 30-50% Δ12  +P: 60-70 % Δ12  PAX: 90% Δ12 | -P: 0%  +P: 10-15% Δ12/9-12  PAX: 0% | -P: 20% Δ12  +P: 30% Δ12  PAX: n.d. | 61% Δ12p(13),  39% Δ12 | agarose gel low informative value for FLT |
|  | % of aberrant transcript(s) in agarose gel in controls | 0% | 0% | 0% | 4% r.1150_1409del p.(Val384Lysfs*8)= Δ12q(260),  1.8% Δ12 |  |
|  | % of aberrant transcript(s) in Sanger sequence in variant-carrying sample | -P: 30-50% Δ12  +P: 60-70% Δ12  PAX: 90% (n.a.) Δ12 | -P: 10% Δ9-12, (5% Δ17)  +P: Σ35%: 15% Δ12, 15% Δ9-12, 5% Δ11-12, <5% Δ12p(13) (5% Δ9, 5% Δ17)  PAX: 5% Δ9-12, 5% Δ12 | -P: 10% Δ12  +P: 30% Δ12 | 61% Δ12p(13),  39% Δ12 | **over-representation of aberrant isoform in TTS** |
|  | % of aberrant transcript(s) in Sanger sequence in controls | -P: 1-2% Δ12^  +P: 3-5% Δ12^ | -P: 0% Δ12^/9-12, 5% Δ17^  +P: 5-10% Δ12^, 0% Δ9-12/11-12, 10% Δ17) | -P: 0%  +P: 0-10% Δ12^ | 4% Δ12q(260),  1.8% Δ12^  (determined by GenScan) |  |
|  | Allelic expression of WT/VUS in Sanger sequence | intronic site, n.a. | intronic site, n.a., no informative variant in transcript | intronic site, n.a. | n.a. |  |
|  | Level of normally-spliced transcript produced by variant allele in % | n.a., intronic site | n.a., intronic site, aberrant splicing of the affected exon is within splicing defect threshold | n.a., intronic site | 0% |  |
|  | Splicing effect and preliminary classification based on splicing evidences | location of variant in conserved splice site, variant allele results in a splicing defect of unknown strength,  analysis cannot rule out generation of ns transcripts from the variant allele (Class 4) | location of variant in conserved splice site, variant allele results in a splicing defect of unknown strength, intensity of aberrant transcripts within splicing defect threshold, analysis cannot rule out generation of ns transcripts from the variant allele (Class 4) | location of variant in conserved splice site, variant allele results in a splicing defect of unknown strength,  analysis cannot rule out generation of ns transcripts from the variant allele, no additional information gained (Class 4) | variant induces a complete splicing defect, but needs supporting evidence (Class 4) | absence of ns transcript from the variant allele could only be demonstrated in minigene assay |
|  | **Final classification*** | **Class 5 (combining minigene with PT/FLT results or bioinformatics prediction)**  The complete splicing defect affecting exon 12 creates isoforms, which lead to a premature stop codon and NMD, or delete functional protein domains (MutSα interaction, NLS, EXO1 interaction). Absence of ns transcripts from the variant allele was shown. | | | | |
| **Gene and VUS (samples)** | **Method** | **TTS MLH1 E11-15 (BCN)** | **FLT MLH1 E1-19 (MUC)** | **TTS MLH1 E11-17 (MUC)** | **pCAS2.MLH1.ex12 minigene EA-B (URO)** | **Differences** |
| ***MLH1***  **Exon 12**  **c.1217G>A**  **p.(Ser406Asn)**  +  *MLH1*  Intron 17  c.1989+3dup p.?  **(-P/+P, PAX1, PAX2)** | Detection of variant-induced aberrant splicing for **c.1217G>A** | No | No | No | No |  |
|  | Aberrant transcript and predicted protein alteration(s) for **c.1217G>A** | none, only variant r.1217G>A  (p.Ser406Asn) | none, only variant r.1217G>A  (p.Ser406Asn) | none, only variant r.1217G>A  (p.Ser406Asn) | None  (Ref. 40) |  |
|  | % of aberrant transcript in agarose gel in variant-carrying sample | -P/+P: 0%  PAX: n.a. | -P/+P: 0%  PAX: n.a. | -P/+P: 0%  PAX: n.d. | 0% | PAX n.a. in FLT + PT |
|  | % of aberrant transcript in agarose gel in controls | 0% | 0-5% | 0% | 0% |  |
|  | % of aberrant transcript in Sanger sequence in variant-carrying sample for **c.1217G>A** | -P/+P: 0% | -P: 0% aberrant transcripts involving E12 (50% del E17)  +P: 0% aberrant transcripts involving E12 (50% Δ17, 5% Δ9-10) | 0%  aberrant transcripts involving E12 | 0% |  |
|  | % of aberrant transcript in Sanger sequence in controls | -P: 0%  +P: 0-5% Δ12^ | -P: 0% aberrant transcripts involving E12  +P: 5-10% Δ12^ | -P: 0%  +P: 0-10% Δ12^ | 0% |  |
|  | Allelic expression of WT/VUS in Sanger sequence for **c.1217G>A** | -P/+P/PAX: r.1217G>A 50%  no allelic reduction | –P/+P: r.1217G>A 50% | –P/+P: r.1217A 100% VUS,  0% WT  -> monoallelic expression due to selection E17 |  | **different results in FLT and specific TTS analysis E11-17 due to selection on presence of E17** |
|  | Level of normally-spliced transcript produced by variant allele **c.1217G>A** in % | 100%, normal splicing of E12, biallelic expression of VUS in E12 | 100%, normal splicing of E12, biallelic expression of VUS in E12, no allelic reduction | 100%, monoallelic expression of E12 VUS r.1217A in –P/+P | 100% |  |
|  | Splicing effect of **c.1217G>A** and preliminary classification based on splicing evidence | Splice-neutral missense variant, unknown significance on protein level (Class 3) | Splice-neutral missense variant,  unknown significance on protein level, unknown allelic status to splicing defect (Class 3) | new information gained: splice-neutral missense variant in E12 is *in trans* to splicing defect IVS17 generating a 100% splicing defect Δ17 | Splice-neutral missense variant,  unknown significance on protein level (Class 3) | additional patient data (genetic and clinical) by combination of FLT and PT transcript analysis **enhanced significance of result** |
|  |  |  | By combining results of FLT and TTS analysis:  variant *in trans* to a pathogenic splicing defect, no indication for CMMR-D in patient with colon cancer at 44 y (Class 2) | |  |  |
|  | **Final classification*** | **Class 1 (combining FLT with TTS results and clinical data)** | | | | |
| **Gene and VUS (samples)** | **Method** | **TTS MLH1 E15-19 (BCN)** | **FLT MLH1 E1-19 (MUC)** | **TTS MLH1 E11-17 (MUC)** | **pCAS2.MLH1.ex17-18 minigene EA-B (URO)** | **Differences** |
| ***MLH1***  **Intron 17**  **c.1989+3dup p.?**  +  *MLH1*  Exon 12  c.1217G>A  p.(Ser406Asn)  **(-P/+P, PAX1, PAX2)** | Detection of variant-induced aberrant splicing for **c.1989+3dup** | Yes | Yes | Yes, indirectly by selecting against isoforms lacking E17 | Yes |  |
|  | Aberrant transcript(s) and predicted protein alteration(s) for for **c.1989+3dup** | r.1897_1989del  (p.Glu633_Glu663del) = Δ17 | r.1897_1989del  (p.Glu633_Glu663del) = Δ17 | None, but allelic loss indicating Δ17 | r.1897_1989del  (p.Glu633_Glu663del) = Δ17, r.1989_1990ins[1989+1_1989+31; 1989+3dup] p.(Asn665Glnfs*) = ▼17q(32) | **different results in FLT and specific TTS analysis due to primer selection on presence of E17** |
|  | % of aberrant transcript(s) in agarose gel in variant-carrying sample | -P: 70% Δ17  +P: 50% Δ17  PAX: 80% Δ17 | -P/+P: 0%, n.a.  PAX: n.a. | -P/+P: 0%, n.a.  PAX: n.d. | 85% Δ17, 15% ▼17q(32) | PAX n.a. in FLT,  agarose gel not informative for FLT |
|  | % of aberrant transcript(s) in agarose gel in controls | 5-10% Δ17 | 0% | 0% | 2% Δ17 |  |
|  | % of aberrant transcript(s) in Sanger sequence in variant-carrying sample for **c.1989+3dup** | -P: 70-80% Δ17  +P: 70-80% Δ17PAX: 70-80% Δ17 | -P: 50% Δ17  +P: 50% Δ17, (5% Δ9-10) | 0%; due to primer selection on transcripts containing E17 | 85% Δ17,  15% ▼17q(32) | **preferential amplification of aberrant isoform in TTS E15-19, different result in TTS E11-17 due to primer selection** |
|  | % of aberrant transcript in Sanger sequence in controls | -P: 5-10% Δ17^  +P: 10% Δ17^ | -P: 5-10% Δ17^  +P: ~10% Δ17^ | 0% Δ17 due to isoform selection by primer in E17 | 2% Δ17^ |  |
|  | Allelic expression of WT/VUS in Sanger sequence for **c.1989+3dup** | intronic site, n.a., | intronic site, n.a., but VUS E12 r.1217G>A 50% in –P, +P states presence of two alleles | intronic, n.a., but VUS E12 r.1217A 100% in –P, +P indicates allelic loss of wt | n.a. |  |
|  | Level of normally-spliced transcript produced by variant allele **c.1989+3dup** in % | n.a. | n.a, aberrant splicing above defined threshold for FLT,  but with unknown allele-specificity and completeness of splicing defect | 0% by conclusion:  allelic loss of E12 wt indicates complete 100% splicing defect Δ17,  VUS E12 is *in trans* to VUS IVS17 | 0% | **FLT combined with TTS transcript analysis shows completeness of splicing defect and allelic status of VUS E12 *in trans*** |
|  | Splicing effect of **c.1989+3dup** and preliminary classification based on splicing evidence | Variant allele results in a splicing defect of unknown strength,  analysis cannot rule out generation of wt transcripts from the variant allele. Cloning or allele-specific PCR including E12 variant c.1217G>A would allow classification of this variant  (Class 3) | Variant allele results in a splicing defect of unknown strength, intensity of aberrant transcripts within splicing defect threshold, analysis cannot rule out generation of ns transcripts from the variant allele (Class 3) | new information gained: complete splicing defect shown by absence of r.1217G, allelic status of two variants determined *in trans* | variant induces a complete splicing defect, but needs supporting evidence (Class 3) | **TTS combined with FLT enhanced significance of result, minigene result confirming cDNA results** |
|  |  |  | allele-specific 100% splicing defect, combined results of FLT analysis showing a strong splicing defect and specific TTS demonstrating completeness of splicing defect, in-frame deletion of E17, but functional protein domain affected (Class 5) | |  |  |
|  | **Final classification*** | **Class 5** (**combining FLT with TTS or minigene results with bioinformatics prediction and literature)**  The complete splicing defect creates an in-frame isoform, which is assumed to affect functional protein domains (EXO1 interaction, MLH3/PMS1/PMS2 interaction). Absence of ns transcripts from the variant allele was shown. | | | | |
