# Supplementary material for: Splicing analyses for variants in MMR genes: best practice recommendations from the European Mismatch Repair Working Group
Source: Eur J Hum Genet. 2022 Jun 9;30(9):1051–9. doi: 10.1038/s41431-022-01106-w (PMC9437034; doi:10.1038/s41431-022-01106-w)
Supplement: Supplementary file 10 — Supplemental Table 5_Summary of in silico splicing predictions [file 41431_2022_1106_MOESM10_ESM.docx]

**Supplemental Table 5:** Summary of *in silico* splicing predictions obtained for the seven variants investigated in this study. The table includes information on the relative position of each variant, whether they are located within reference splice sites’ consensus sequences, splicing-dedicated predictions based on the MaxEntScan (MES), SpliceSiteFinder-like (SSFL) and SpliceAI algorithms and whether a splicing defect is expected (also highlighted by the grey background, according to thresholds indicated in Supplementary Methods). AL, acceptor loss; AG, acceptor gain; DL, donor loss, DG, donor gain.; n.a., not available. (*) intronic sequence that may create an in-frame novel donor site.

| **Gene and variant** | **variant location** | **MES and SSFL predictions** | **SpliceAI prediction**  **(Scores/ Nucleotide distance**  **relative to variant of interest)** | | | | **Interpretation** | **Splicing defect is expected?** |
| --- | --- | --- | --- | --- | --- | --- | --- | --- |
|  |  |  | **AL** | **DL** | **AG** | **DG** |  |  |
| ***MSH2* c.211G>C** | last nucleotide of exon 1 | ΔMES -43%  ΔSSFL -15% | 0.00  (n.a.) | 0.40  (0) | 0.01  (466) | 0.67  (-17) | - splice donor site strength is drastically reduced  - generation of a novel splice donor site with higher scores than those of the usual splice donor site  -intron sequence following is not GTRRGT* | yes |
| ***MSH2* c.1276G>A** | last nucleotide of exon 7 | ΔMES -79%  ΔSSFL -15% | 0.04  (-119) | 0.91  (0) | 0.00  (n.a.) | 0.58  (-48) | - splice donor site strength is drastically reduced  - generation of a novel splice donor site with lower scores than those of the usual splice donor site  -intron sequence following is not GTRRGT* | yes |
| ***MSH2* c.2459-12A>G** | 12 nucleotides before exon 15 | ΔMES -42%  ΔSSFL -100% | 0.73  (12) | 0.01  (-69) | 0.98  (1) | 0.00  (n.a.) | - splice acceptor site strength is reduced  -generation of a novel splice acceptor site with higher scores than those of the usual splice acceptor site | yes |
| ***MSH6* c.1894A>G** | middle of exon 4 | reference splice sites are far away in both directions | 0.00  (n.a.) | 0.00  (n.a.) | 0.02  (-39) | 0.02  (-288) | - no splicing effect is predicted | no |
| ***MLH1* c.1039-2A>T** | penultimate nucleotide before exon 12 | ΔMES -100%  ΔSSFL -100% | 0.92  (2) | 0.39  (372) | 0.32  (15) | 0.00  (n.a.) | - splice acceptor site strength is abolished  - generation of a novel splice acceptor site with lower scores than those of the usual splice acceptor | yes |
| ***MLH1* c.1217G>A** | middle of exon 12 | no obvious effect upon splice sites in both directions | 0.00  (n.a.) | 0.01  (-68) | 0.00  (n.a.) | 0.00  (n.a.) | - no splicing effect is predicted  – cryptic acceptor site abolished and creation of a new one 4 nucleotides downstream according to MES and SSFL | no |
| ***MLH1***  **c.1989+3dup** | 5` end of intron 17 | ΔMES -100%  ΔSSFL -100% | 0.28  (-98) | 0.68  (-3) | 0.00  (n.a.) | 0.85  (28) | - splice donor site strength is abolished  - intron sequence following is not GTRRGT* | yes |
