# Supplementary material for: Splicing analyses for variants in MMR genes: best practice recommendations from the European Mismatch Repair Working Group
Source: Eur J Hum Genet. 2022 Jun 9;30(9):1051–9. doi: 10.1038/s41431-022-01106-w (PMC9437034; doi:10.1038/s41431-022-01106-w)
Supplement: Supplementary file 11 — Supplemental Table 6_List of advantages and disadvantages of the different experimental and bioinformatical splicing analyses [file 41431_2022_1106_MOESM11_ESM.docx]

**Supplemental Table 6:** List of advantages and disadvantages of the different experimental and bioinformatical splicing analyses performed in this study.

| **Method** | **TTS analysis** | **FLT analysis** | **Minigene assay** | **Bioinformatical predictions** |
| --- | --- | --- | --- | --- |
| **Advantages** | - robust analysis even for low-quality cDNA  - can be designed to select for or against specific variants/isoforms  - easy interpretation | - universal application for all coding variants in MMR genes  - pre-defined thresholds for splicing defect, alternative splicing, allelic balance and allelic loss  -allelic determination of VUS and informative variants enhances significance of result  - detection of more isoforms splicing several exons  - limited PCR-bias to shorter isoforms  - significant classification for in-trans variants  - applicable for unsolved patients  - discrimination against *PMS2* pseudogenes | - highly useful for intronic variants  - independent from patient material availability  - monoallelic character of the assay facilitates result interpretation  - no NMD inhibition needed in the case of pCAS2-derived minigene constructs  - high-sensitivity in isoforms detection | - Fast  - Reliable for consensus splice site variants |
| **Dis-advantages** | - patients’ RNA needed  - informative variant needed for a significant result  - Visualization of RT-PCR products in agarose gels provides an approximate estimation of splicing isoforms relative levels  - Sanger sequencing provides an approximate estimation of relative allelic expression  - manual analysis with hands-on-time  - splicing pattern in blood cells may vary from disease-relevant tissue  - limited significance for intronic variants (depending e.g. on informative exonic variants) | | - time-consuming establishment  - not yet validated for all exons/genes  - splicing pattern in minigene constructs may vary from patient RNA  - choice of transfected cell line and minigene structure can influence splicing defect severity  - minigene result alone not sufficient for VUS classification according to current classification guidelines | - Unable to predict the exact nature of the splicing defects  - less reliable for variants located outside the splice sites |
|  | - PCR-bias leads to over-representation of short isoforms  - may miss isoforms depending on primer location | - amplification requires high-quality RNA  - interpretation of results requires some training |  |  |
